# Aqueous outflow regulation – 21st century concepts

**DOI:** 10.1016/j.preteyeres.2020.100917

**Published:** 2020-11-17

**Authors:** Murray Johnstone, Chen Xin, James Tan, Elizabeth Martin, Joanne Wen, Ruikang K. Wang

**Affiliations:** a Department of Ophthalmology, University of Washington, USA; b Department of Bioengineering, University of Washington, USA; c Department of Ophthalmology, Beijing Anzhen Hospital, Capital Medical University, China; d Indiana University, Department of Ophthalmology, USA; e Doheny Eye Institute and UCLA Department of Ophthalmology, USA; f Duke Eye Center, Duke University, USA

**Keywords:** Aqueous outflow pump, Intraocular pressure regulation, Glaucoma, Elastance, Pulsatile aqueous outflow, Schlemm’s canal valves

## Abstract

We propose an integrated model of aqueous outflow control that employs a pump-conduit system in this article. Our model exploits accepted physiologic regulatory mechanisms such as those of the arterial, venous, and lymphatic systems. Here, we also provide a framework for developing novel diagnostic and therapeutic strategies to improve glaucoma patient care. In the model, the trabecular meshwork distends and recoils in response to continuous physiologic IOP transients like the ocular pulse, blinking, and eye movement. The elasticity of the trabecular meshwork determines cyclic volume changes in Schlemm’s canal (SC). Tube-like SC inlet valves provide aqueous entry into the canal, and outlet valve leaflets at collector channels control aqueous exit from SC. Connections between the pressure-sensing trabecular meshwork and the outlet valve leaflets dynamically control flow from SC. Normal function requires regulation of the trabecular meshwork properties that determine distention and recoil. The aqueous pump-conduit provides short-term pressure control by varying stroke volume in response to pressure changes. Modulating TM constituents that regulate stroke volume provides long-term control. The aqueous outflow pump fails in glaucoma due to the loss of trabecular tissue elastance, as well as alterations in ciliary body tension. These processes lead to SC wall apposition and loss of motion. Visible evidence of pump failure includes a lack of pulsatile aqueous discharge into aqueous veins and reduced ability to reflux blood into SC. These alterations in the functional properties are challenging to monitor clinically. Phase-sensitive OCT now permits noninvasive, quantitative measurement of pulse-dependent TM motion in humans. This proposed conceptual model and related techniques offer a novel framework for understanding mechanisms, improving management, and development of therapeutic options for glaucoma.

## 1. Introduction

Glaucoma is a leading cause of irreversible blindness resulting in optic nerve damage and visual field loss (Susanna et al., 2015). Lowering intraocular pressure (IOP) remains the only treatment strategy that slows or prevents disease progression (Heijl et al., 2009). An accurate understanding of mechanisms controlling IOP can lead to improvements in the treatment of the disease. The traditional understanding of IOP regulation has rested on indirect clinical measurements and laboratory studies, but the related hypotheses involve assumptions not easily verifiable.

Objective clues to the nature of aqueous outflow are evident in humans, in whom we can directly see the return of aqueous humor to the episcleral veins. An important observation is that flow into the episcleral veins is pulsatile, indicating the existence of a vascular pump. Pulsatile aqueous outflow diminishes and eventually stops in glaucoma patients (Ascher, 1961). These observations should not be overlooked, and it seems important that they are integrated into any theoretical framework explaining aqueous outflow.

Pulsatile aqueous outflow behavior provides a framework for integrating structural and functional evidence into our 21st-century concept of aqueous outflow. Here, we connect unifying lines of evidence supporting our proposal that the outflow system incorporates a regulatory pump akin to those present in the rest of the vasculature. We characterize this phenomenon in health and disease in the hope that it will lead to a better understanding of the disease process’s pathophysiology, diagnosis, and treatment.

### 1.1. Contrasting theories of outflow regulation – a passive filter or a pump?

#### 1.1.1. A filter

Reports of investigators in the 19th-century provide contradictory theories of IOP regulatory mechanisms. Schwalbe concluded that the outflow system is like the lymphatics with open pathways in communication with the venous system (Schwalbe, 1870). Leber identified a border region adjacent to Schlemm’s canal (SC) that he interpreted as preventing direct passage of aqueous into the canal. He hypothesized that a passive filtration process controls aqueous flow across SC endothelium (Leber, 1873).

In a current version of Leber’s theory of the location of outflow resistance, aqueous flows passively down a pressure gradient and enters a regulated extracellular matrix (ECM) filter in the juxtacanalicular space. The filter funnels aqueous to transendothelial pores in the endothelium of SC inner wall endothelium (Johnson et al., 2002). The hypothesis proposes that modulation of the ECM and SC endothelium properties maintains IOP homeostasis and forms the premise for numerous studies. Thorough discussions of proposed homeostatic mechanisms and relevant supporting references for the traditional view are available in the following articles (Stamer et al., 2015; Acott et al., 2020).

Grant’s early work (Grant, 1958; Grant, 1963) lent support to Leber’s theory because he found experimental removal of the trabecular meshwork (TM) could eliminate 75% of the resistance in enucleated eyes. However, a reexamination of the earlier study’s experimental conditions and later studies by Grant and colleagues led to a different conclusion. Removal of the distal wall of SC was as effective as removing the TM in reducing resistance (Section (§) 3.8.1). The findings led them to conclude that the measured resistance results from the TM coming into contact with the SC external wall instead of resistance being localized within the TM (Ellingsen and Grant, 1972). Also, clinically, removing SC external wall improves aqueous outflow and IOP as much as removing the TM (Nesterov, 1970; Grieshaber et al., 2015). Evidence that the TM and distal system play synergistic roles in controlling resistance points to the benefit of developing a unifying model of outflow regulation.

#### 1.1.2. A pump

Evolving evidence supports Schwalbe’s conclusion that the outflow system functions like lymphatic pumps. Lymphatic vessels have valves, with sections between valves termed lymphangions that function as miniventricles or chambers. Extrinsic forces such as pulsations of adjacent arteries and tissue motion cause intermittent lymphangion compression.

**Fig. 1.**
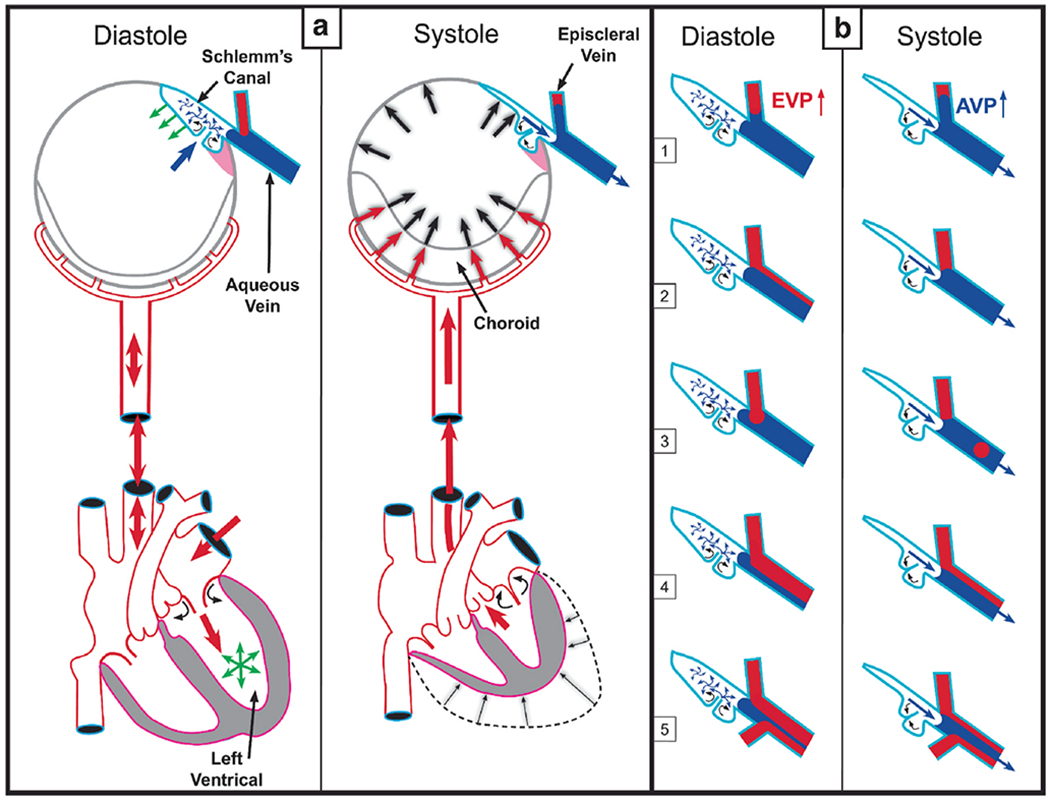
Cardiac-induced pulsatile aqueous outflow mechanisms. Cardiac source of **(a)** pulsatile aqueous outflow and **(b)** resultant pulsatile flow into the aqueous veins. During systole, the left ventricle contracts, which initiates a pulse wave that causes the choroidal volume to expand, thus increasing intraocular pressure (IOP). Increased IOP causes the trabecular meshwork (TM) to move into the lumen of Schlemm’s canal (SC), narrowing it. TM movement that narrows the SC lumen increases pressure in SC. Reduced space and increased pressure in SC favor the pulsatile flow of aqueous from SC into aqueous and episcleral veins (ESV). Movement creates an aqueous pulse wave and increases aqueous vein pressure (AVP) during systole. During diastole, choroidal volume decreases, and IOP falls. The TM recoils, releasing the potential energy stored during systole. TM recoil reduces pressure in SC, favoring aqueous flow from the AC into SC through SC inlet valves, **(b)** Various manifestations of oscillatory pulsatile flow into the episcleral veins are synchronous with the ocular pulse. Pressure in the aqueous veins falls as SC pressure decreases during diastole. Episcleral venous pressure (EVP) is then transiently higher than aqueous vein pressure (AVP) resulting in oscillatory blood entry into aqueous veins. The next systolic wave causes the AVP to be higher than EVP. From: Johnstone M, Aqueous Veins, The Glaucoma Book. New York: Springer, 2010:65–78. Video - Pulsatile Aqueous Vein Flow 1-s2.0-S1350946220300896-mmc1.mp4).

The compression adds energy to the lymph propelling it through the unidirectional valves to the adjacent lymphangion. Subsequent recoil of the chamber walls permits the lymphangions to act as pumps (Quick et al., 2007, 2009; Levick, 2010). Intrinsic control of the properties of the lymphatic pumps provides highly regulated interstitial pressure homeostasis. Like in the lymphatics, the cardiac cycle’s systole and diastole cause continuous oscillations. In the eye, choroidal volume changes result in the generation and transmission of the ocular pulse to the entire eye. The ocular pulse results in oscillatory cyclic pulse waves impinging on the TM (Fig. 1a). We find evidence of pulse-dependent flow into SC, into collector channels, and into the aqueous veins, where we observe multiple manifestations of aqueous pulse waves entering and displacing blood in the episcleral veins (Fig. 1b).

**Fig. 2.**
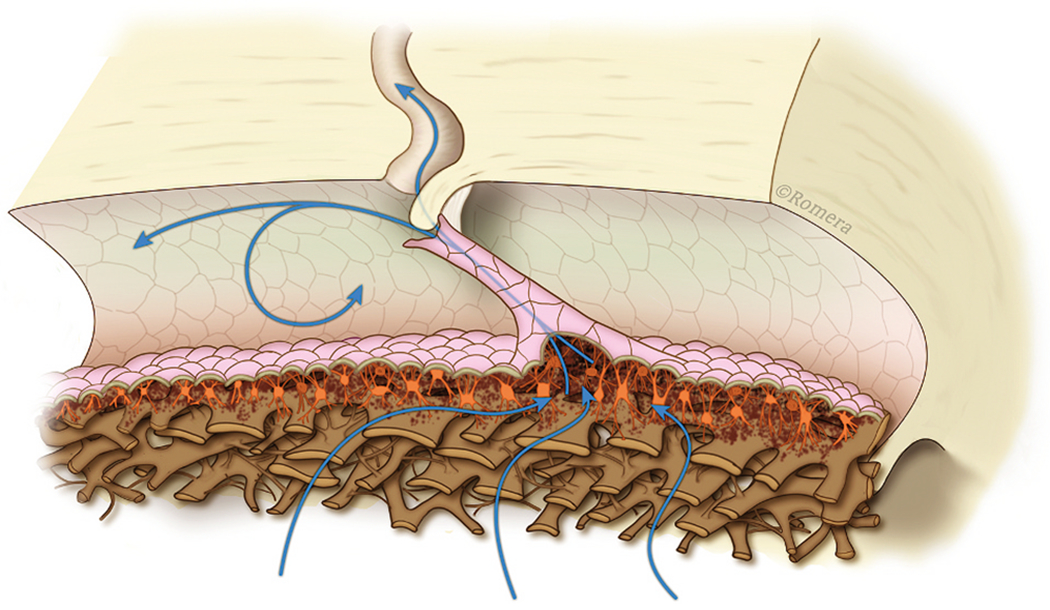
Path of aqueous flow from the anterior chamber to collector channels. The blue arrows depict the passage of aqueous from the anterior chamber through passages within the trabecular lamellae that lead into the juxtacanalicular space. From the juxtacanalicular space, aqueous passes through the lumen of the Schlemm’s canal (SC) inlet valves, where it flows into SC. From SC, aqueous enters a collector channel (CC) entrance through an open SC inlet valve. SC endothelium (SCE) is continuous with the SC inlet valve. The SC inlet valve connections link the TM to the hinged, mobile SC outlet valve. Illustration conceived and developed by Antonio Moreno-Valladares (University Hospital of Albacete, Spain) and Manuel Romera (www.ilustracionmedica.es). Video – SC Inlet Valve Aqueous Flow 1-s2.0-S1350946220300896-mmc2.mp4).

We also find compressible chambers and valves with synchronous pulse-dependent movement that can predict and explain pulsatile aqueous flow from SC (Fig. 2). This constellation of findings leads us to propose that a highly dynamic lymphatic-like pump controls aqueous outflow and IOP. The current report further describes mechanisms of pump failure in glaucoma as well as resultant diagnostic and therapeutic implications. Finally, we explore pump-dependent mechanisms like those in the systemic vasculature known to maintain short and long-term pressure homeostasis.

This article limits exploration to the outflow pump’s intrinsic features that become abnormal in glaucoma in contrast to specific extrinsic glaucoma causes such as angle-closure or inflammation. Here, the term “glaucoma” refers to primary open-angle glaucoma (POAG), which embodies the intrinsic glaucoma abnormality. Some aqueous outflow occurs by a uveoscleral pathway, which is not thought to be actively regulated (Gabelt and Kaufman, 2005), and is outside the scope of this report.

### 1.2. Structural and functional properties of the aqueous outflow pump

Briefly, transient increases in IOP provide power for the aqueous outflow pump (Johnstone, 2004). These cyclic oscillations cause the TM’s elastic structural elements to deform, including the TM lamellae, the juxtacanalicular cells, and SC inner wall endothelium. The TM tissues move outward into SC during systole, opening external wall valves that permit aqueous to flow into collector channels (CC), intrascleral channels, and finally, the aqueous veins. Simultaneously, the IOP increase of systole forces aqueous into the entrances of SC inlet valves.

As the pressure spike decays, the elastic elements return to their original configuration with a resultant reduction in SC pressure. The reduced SC pressure causes aqueous to flow into the canal through the SC inlet valves, and the cycle repeats. Short-term IOP homeostasis results from an increase in pump efficiency as IOP increases. Long-term homeostasis results from regulating the ability of the cells and tissues to distend and recoil. We present clinical and laboratory evidence for this aqueous outflow model and draw parallels to similar tissue-based pumps in other body systems.

## 2. Clinical science: an aqueous outflow pump that fails in glaucoma

### 2.1. Clinical physiology – Aqueous outflow pump function in normal subjects

#### 2.1.1. Pulsatile aqueous flow– A fundamental discovery of the 20th century

**Fig. 3.**
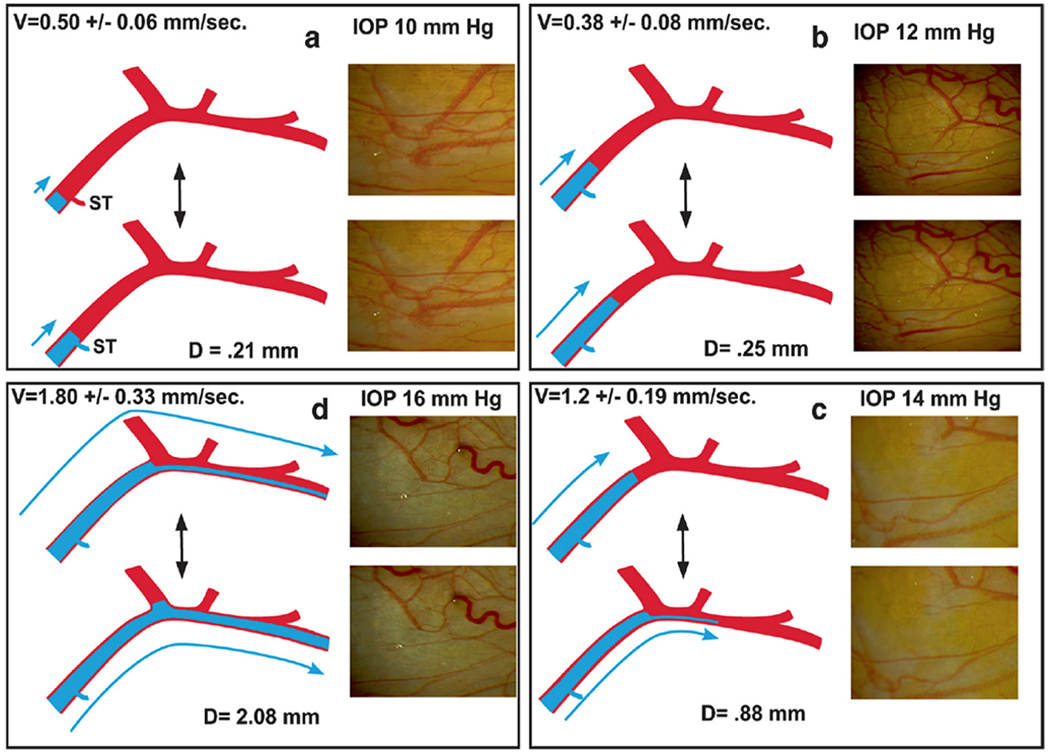
Aqueous pulse wave distance and velocity profile vs. intraocular pressure. Fig. 1 demonstrates the source of the oscillatory pulse waves found in the aqueous veins. Aqueous veins show oscillatory aqueous discharge into episcleral veins. Increased stroke volume increases aqueous outflow, **(a, b, c, d)** Stroke volume responses in a 59-year-old Caucasian male after an increase in intraocular pressure (IOP) following a water-drinking test. Outflow medications that reduce IOP have a similar initial increase in the pulsatile flow until IOP falls to a new lower setpoint, **(a)** Baseline IOP: velocity (V) is low. The aqueous pulse wave travels a short distance (D) with each stroke. Oscillation of a standing transverse wave results in a systolic discharge of aqueous fluid into a small venous tributary (ST), **(b)** Increased distance traveled by the oscillatory aqueous pulse wave, **(c)** A further increase in velocity and travel of the aqueous pulse wave. At each systole, a lamina of clear aqueous discharges into an episcleral vein, **(d)** Additional velocity increase and increased travel of the pulse wave. Continuous oscillating laminar flow now occurs in a more distal episcleral vein. Two hours after drinking water, IOP was again 10 mm Hg, and stroke volume returned to the appearance in **(a)**. Illustrations from: Johnstone M, The Glaucoma Book. New York: Springer, 2010:65–78. Vessel Images from Johnstone M, Aqueous Veins, J Glaucoma 13, 421 438, 2004. Video – Stroke Volume Control of IOP 1-s2.0-S1350946220300896-mmc3.mp4.

We have unparalleled evidence of physiologic outflow mechanisms provided through in vivo, direct noninvasive, observations of aqueous flow into episcleral veins (Goldmann, 1946a) Pulsatile Aqueous Vein Flow Video (Vid 1) 1-s2.0-S1350946220300896-mmc1.mp4). A large body of literature documents directly observable evidence of abnormal pulsatile flow in glaucoma (Ascher, 1942a, 1944, 1949a; Goldmann, 1948; Ascher and Spurgeon, 1949b; Kleinert, 1951c; Johnstone et al., 2010) (Fig. 3). This ability to see pulsatile aqueous outflow and how it becomes abnormal in glaucoma provides an extraordinary opportunity to characterize outflow tissues involved in pulsatile motion and how they lose their ability to induce pulsatile flow.

Direct observation of outflow is free of assumptions and therefore provides a foundational body of knowledge that does not require conjecture. The objective findings determine requirements and constraints that the intrinsic flow pathways must meet to be incorporated into a model of normal outflow mechanisms and related abnormalities in glaucoma. Aqueous Outflow Pump Video (Vid 2) (1-s2.0-S1350946220300896-mmc2.mp4). William Harvey’s studies exemplify the benefits of starting with an in vivo functional approach through his ability to solve the multi-millennial mystery of the pulsatile circulation of blood (Harvey, 1970).

#### 2.1.2. Pulsatile flow into aqueous and episcleral veins: A salient outflow property

Aqueous flows from SC through collector channel entrances into scleral channels, then into aqueous-filled veins visible on the surface of the eye that join blood-filled episcleral veins. At the interface between aqueous and episcleral veins, mixing veins are present. The mixing veins can experience oscillatory aqueous and blood mixing or be filled by only aqueous or blood depending on pressure conditions (Fig. 3). Ascher and Goldmann, the first clinician-scientists to identify flow into aqueous veins, reported pulsatile flow as a salient feature (Ascher, 1942a; Goldmann, 1946a). Pulsatile outflow originates in SC and is synchronous with the ocular pulse (Ascher, 1961; Johnstone et al., 2010). The ocular pulse arises from changes in choroidal volume that occur with the cardiac cycle (Phillips et al., 1992). Many reports document pulse-dependent patterns of aqueous flow shown in Fig. 3. Stepanik’s technique quantifies aqueous vein contributions to aqueous outflow by measuring the stroke volume of pulse waves of aqueous entering aqueous veins (Stepanik, 1954). His work also provides a means of considering how the aqueous outflow pump’s stroke volume can control IOP. IOP Regulation Video (Vid 3) 1-s2.0-S1350946220300896-mmc3.mp4.

Only two aqueous veins are necessary to account for the total volume of outflow. (Stepanik, 1954). Under physiologic, noninvasive conditions, two to three, and at most five aqueous vein complexes carry aqueous (Ascher, 1961). The detailed studies of De Vries under noninvasive conditions in humans document that aqueous outflow is highly asymmetric and concentrated in the inferior hemisphere (87%), particularly the inferior nasal quadrant (56%) (De Vries, 1947). The patterns are likely stable for a lifetime (Ascher, 1961). The recent development of noninvasive angiography using hemoglobin video imaging (Khatib et al., 2019) and invasive operating room angiography (Huang et al., 2017a) provide new tools to aid in our understanding of aqueous outflow physiology and glaucoma.

#### 2.1.3. Pulsatile flow into CC and the distal pathways

**Fig. 4.**
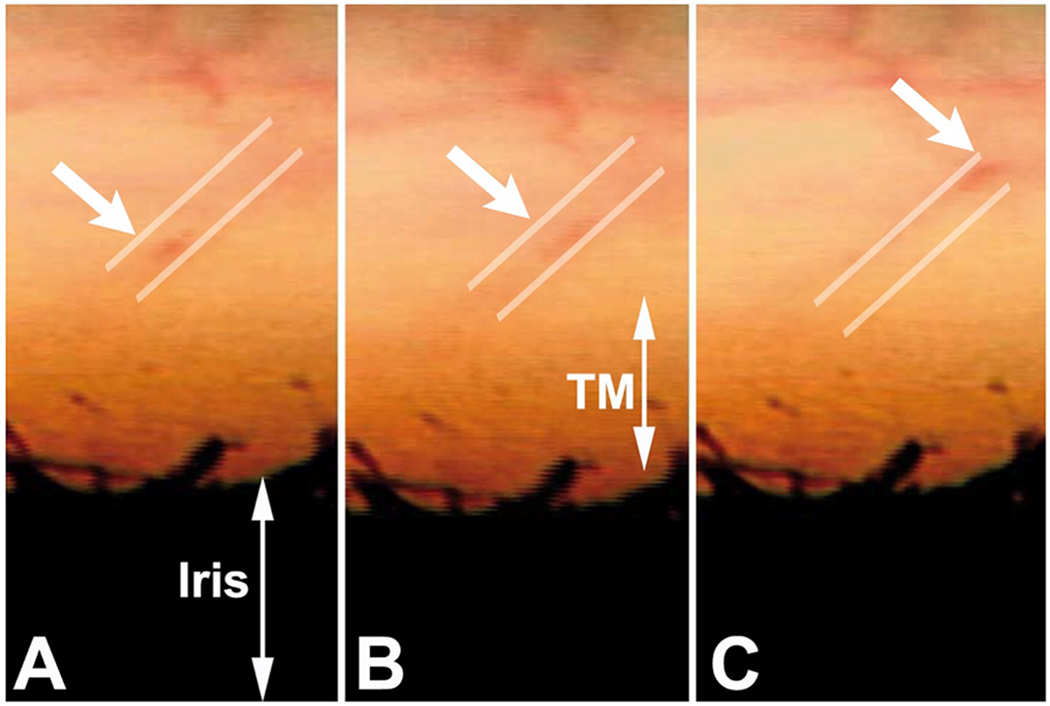
Pulsatile aqueous flow from Schiemm’s canal into collector channels. Pulsatile aqueous flow from SC into collector channels and more distal intrascleral channels in a human subject. Parallel white lines above the trabecular meshwork depict the course of the flow of blood-tinged aqueous (white arrows). **A-C** are sequential video frames encompassing one systolic pulse wave. (Gonioscopic video courtesy of R. Stegmann) From Grehn, H., Stamper, R., Essentials in Ophthalmology: Glaucoma II. Springer, Heidelberg, 2006.

Stegmann’s technology (Sec. 4.1.2) captures an oscillating column of blood-tinged aqueous moving from SC into a distinct distal CC pathway with each systolic pulse wave (Johnstone, 2004) (Fig. 4). Synchrony with the ocular pulse is evidence that flow rates in the distal system are like those of the systemic vasculature that are known to induce shear stress. The clinical research findings implicate shear stress as well as both nitric oxide and endothelin in SC and distal outflow regulation (Johnstone, 2004).) (See §5.0 detailed shear stress discussion.)

#### 2.1.4. Pulsatile flow into Schiemm’s canal through SC inlet valves

**Fig. 5.**
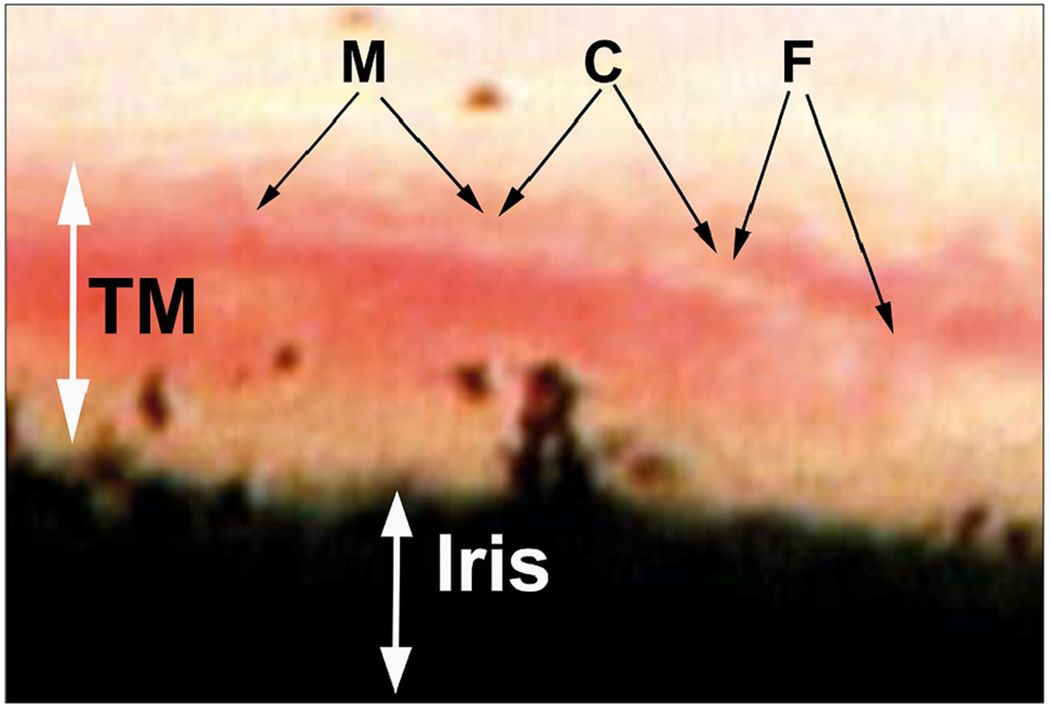
Pulsatile aqueous flow from the anterior chamber into Schiemm’s canal. Pulsatile aqueous flow through a Schiemm’s canal (SC) inlet valve in synchrony with the ocular pulse. Blood reflux into SC creates a red background that is visible through the transparent trabecular meshwork (TM). The stable background of blood contrasts with the propagating wave of clear aqueous moving through the SC inlet valve with each cardiac pulse wave. The aqueous always moves along a constrained path, indicating that the surrounding tissues determine the course of flow. Dimensions of the aqueous-defined pathway correspond to those of the aqueous inlet valves seen in laboratory studies. Cyclic enlargement of the aqueous filled funnel (F) then proceeds to collapse of the funnel as the propagating aqueous wave moves into the enlarging cylindrical region (C). The propagation of the pulse wave into the tube-area continues, followed by the closure of the tube-like area as a stream of clear aqueous is propelled into SC. Ejection of the propagating aqueous stream into SC results in swirling eddies of aqueous and blood mixing (M) in SC. (Gonioscopic video courtesy of R. Stegmann) From Johnstone M, An Aqueous Outflow Pump and its failure in glaucoma, Essentials in Ophthalmology: Glaucoma II. Springer, Heidelberg, 2006. Video Flow to SC Through SC Inlet Valves 1-s2.0-S1350946220300896-mmc2.mp4).

Troncoso’s report (Troncoso, 1925) is the first to recognize stratification “due to a small amount of blood circulating in Schiemm’s canal” surrounding a clear region and “the analogy to stratification in aqueous veins is striking” (Ascher, 1961). Stegmann’s technology (Sec. 4.1.2) demonstrates comparable stratification, and his imaging shows the origin of the stratification. Pulsatile waves of clear aqueous propagate from the base of a funnel-shaped region to a cylindrical region, then enters SC, where swilling eddies of blood and aqueous mix (Johnstone, 2004) (Fig. 5) (Vid 2). The cylindrical region of clear aqueous and the more distal aqueous column surrounded by blood are like Trocoso’s observations.

Evidence of the oscillatory appearance of aqueous against a background of blood is present in this and other Stegmann videos. However, the video referenced above is the only one that captures thirty oscillatory sequences involving the full length of the propagating aqueous wave from its funnel base to final discharge into SC.

Stegmann’s demonstration of propagating pulsatile aqueous flow into SC through a pathway constrained to the configuration of an aqueous inlet valve marks a significant milestone in our understanding of the outflow system function. His videomicroscopy provides directly observable, phenomenological evidence that aqueous flow into SC can occur by a cyclic, pulsatile, propagating aqueous wave (Johnstone, 2004; Johnstone et al., 2009)abs.

Laboratory studies independently identify an SC inlet valve that constrains flow to the relevant configuration we see in vivo (Johnstone, 1974, 2004) (§3.5). Pulsatile aqueous movement through an inlet valve enters SC in a propagating wave that remains synchronous with the ocular pulse. The pulsatile behavior exposes the endothelial cells of the walls of SC to the rapidly moving fluid wave. We thus have evidence of pulsatile aqueous flow in SC that occurs in synchrony with the flow speeds that induce shear stress responses in endothelial cells throughout the systemic vasculature (Johnstone, 2004, 2006, 2009).

#### 2.1.5. Imaging of pulsatile TM motion by phase-based OCT (PhS-OCT)

**Fig. 6.**
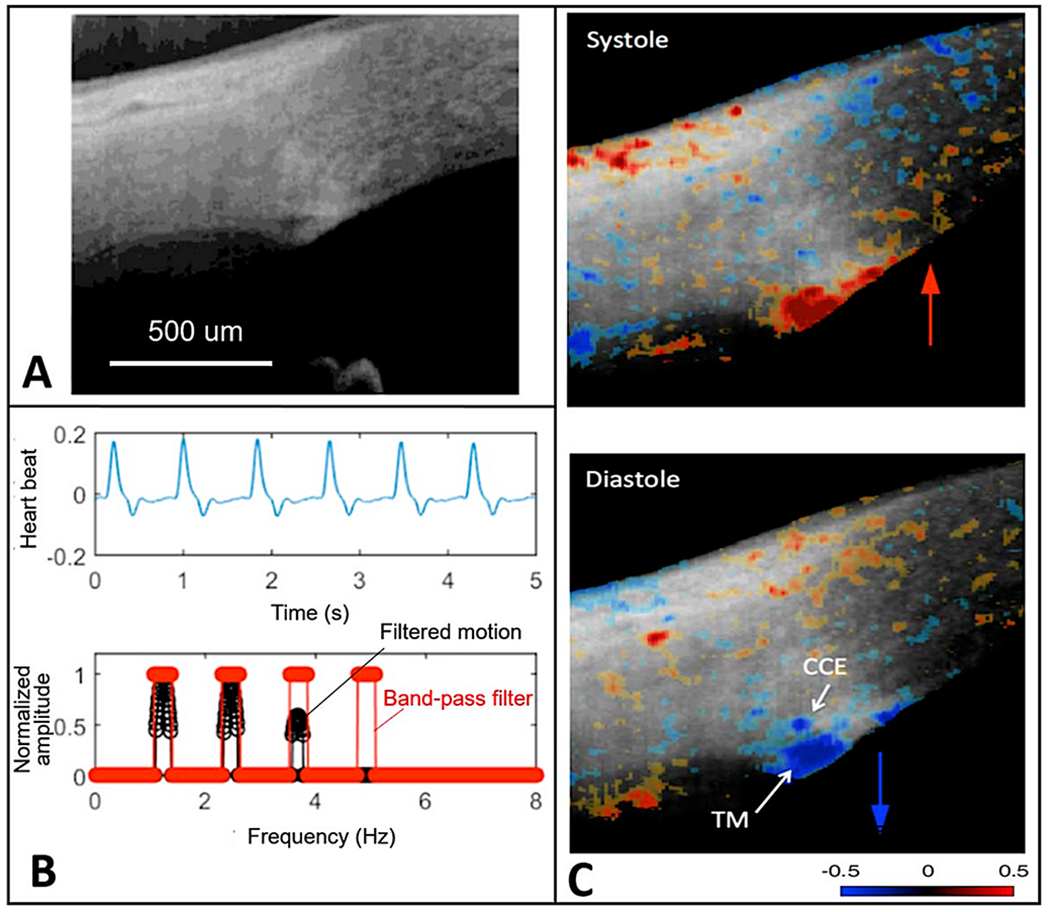
Synchrony of color-encoded trabecular meshwork and cardiac pulse. **(A)** Representative structural image captured by OCT. **(B)** Heartbeat signals obtained from a digital pulsimeter shows the frequency domain positions (red markings). The trabecular meshwork (TM) motion signal is the filtered frequency domain TM motion (black trace, synchronized with the heartbeat signal. **(C)** Color-encoded instantaneous velocity is overlaid on the structural image. Red indicates anterior tissue movement toward the probe above the scleral surface. Blue indicates posterior tissue movement toward the anterior chamber. Tissue source: Human subject. From Xin C, Pulse-dependent TM motion in normal humans using phase-sensitive OCT. Invest Ophthalmol Vis Sci 59, 3675–3681, 2018.

We use a PhS-OCT system, relevant algorithms, and a digital pulsimeter to characterize pulse-dependent motion in living human subjects. A recent study by Xin demonstrates excellent reliability and repeatability (Fig. 6) (Xin et al., 2018). The resolution of motion is sufficiently sensitive to identify movement differences in the inner and outer locations of the TM, as well as motion changes resulting from accommodation efforts.

### 2.2. Clinical pathophysiology – aqueous outflow pump failure in glaucoma

#### 2.2.1. Pulsatile flow stops as glaucoma progresses

Pulsatile flow becomes less vigorous, slows, and then stops as glaucoma progresses. For example, Kleinert found 196 pulsating aqueous veins in 111 healthy eyes corresponding to 27.7% of all the veins in the group. In subjects with aortic regurgitation, eighteen eyes of nine subjects all had both high pulse pressures and aqueous vein pulsations. In contrast, Klienert found no pulsatile flow in glaucoma patients with pressures over 28 mm Hg (Kleinert, 1951a, 1951b).

#### 2.2.2. TM motion loss as glaucoma progresses

Clinical compression of the episcleral veins during gonioscopy causes a reversal of the usual pressure gradient between the anterior chamber (AC) and SC. Pressure in SC is then higher than in the AC, and the TM moves toward the AC as blood refluxes into the canal (Smith, 1956; Schirmer, 1969, 1971; Suson and Schultz, 1969; Phelps et al., 1972). SC filling begins in 5–10 s and finishes in 15–30 s (Schirmer, 1971). Equally rapid elimination of blood results from the restoration of physiologic pressure gradients.

In eyes with ocular hypertension, the rapidity of SC filling decreases, but the canal still fills, and the outflow facility remains near normal. “Blood-filling defects of the canal gradually appear with increasing frequency and severity, closely paralleled by deteriorating outflow facility. It is reasonable to believe … the initial reduction of the outflow facility … was due to compression of the inner wall against the outer wall of SC with restriction of the effective filtration area. Subsequent aggravation of the impaired facility most likely resulted from damage to SC inner wall, …and its adhesion to the outer wall demonstrated gonioscopically as blood filling defects.” (Suson and Schultz, 1969).

In advanced glaucoma, blood fails to reflux into SC, even with very aggressive measures to reverse pressure gradients (Kronfeld, 1949). The lack of ability to reflux blood into SC as the glaucoma process advances provides a means to separate normal subjects from ocular hypertensives and those with glaucoma (Schirmer, 1969, 1971). Investigators consider evidence of the inability to reflux blood in advanced glaucoma to result from irreversible trabecular tissue stiffening or adhesion to the SC external wall of SC (Suson and Schultz, 1969). Persistent IOP elevation may result in the chronic compression of trabecular tissue against the external wall and an undesirable cycle of further IOP elevation. The investigators suggest that earlier detection and treatment may reduce the progression and severity of the glaucomatous process (Schirmer, 1969, 1971).

#### 2.2.3. Clinical tests of pulsatile flow failure in glaucoma

Gentle pressure on the eye through the lid results in a transient, more vigorous pulsatile aqueous flow into the episcleral veins. IOP then falls to slightly lower than the baseline homeostatic pressure (Goldmann, 1946a, 1946b; Johnstone et al., 2011c). Pressure below the homeostatic level results in blood reflux into areas of veins that previously had a stable oscillating column of aqueous humor. Over the next minute or two, the veins refill with an advancing aqueous interface. The advancing oscillating aqueous column finally stabilizes at the same vessel location present before manipulation, thus returning the aqueous column to the previous homeostatic setpoint (De Vries, 1947; Thomassen, 1947a).

The “compensation maximum” test uses a pressure-induced increase in pulsations to identify glaucoma abnormalities. The test increases IOP with pressure from an ophthalmodynamometer while imaging aqueous veins. Pulsatile outflow increases as IOP increases in normal subjects, but in glaucoma patients, pulsatile outflow slows or stops (Kleinert, 1951c; Stambaugh et al., 1954).

The “water-drinking” test rapidly increases IOP and results in a corresponding increase in the stroke volume of aqueous (Johnstone, 2004; Johnstone et al., 2011c) (Fig. 3). The stroke volume increase induces increased aqueous outflow, thus reducing IOP. As IOP decreases, stroke volume also gradually decreases until IOP returns to its resting state and stroke volume also returns to prior status. The responses demonstrate the linkage between the amplitude of stroke volume and IOP. An increased volume of aqueous in the aqueous veins precedes diurnal reductions in IOP while reducing aqueous volume precedes diurnal elevations in IOP (Ascher, 1944; Thomassen, 1947a, 1947b; Goldmann, 1948; Thomassen et al., 1950).

#### 2.2.4. Asymmetric flow into the aqueous veins - clue to circumferential SC flow?

Noninvasive aqueous angiography techniques of Ascher explore the normal circumferential distribution of aqueous flow into the episcleral veins on the eye’s surface in healthy humans (Ascher, 1942a). Typically, only two or three aqueous veins are visible on the eye’s surface, and a quantitative study indicates that only two such veins can carry all the aqueous flow (Stepanik, 1954a, 1957; Stepanik and Kemper, 1954b; Ascher, 1961).

Noninterventional studies using blood as the aqueous angiography tracer demonstrate that aqueous discharge from SC to the episcleral veins on the surface of the limbal circumference is not uniform but rather is highly asymmetric. The classic study of De Vries reports that eighty-seven percent of the aqueous flow into the episcleral veins is in the inferior quadrants, with fifty-six percent of the total flow in the inferior nasal quadrant (De Vries, 1947). The asymmetry of aqueous flow from SC into episcleral veins is likely unchanging over a lifetime (Ascher, 1961). A nasal signal in the episcleral veins is also the pre-dominant finding in ex vivo and live eyes with invasive angiography (Huang et al., 2016b, 2017a, 2017b; Saraswathy et al., 2016).

Aqueous veins responding to the cyclic ocular pulse are also the ones that respond to eye movements and blinking (Ascher, 1961) (Martin et al., 2010)abs. The highly asymmetric aqueous flow constrained to a localized area of the circumference is puzzling. The circumference of the TM and SC exhibits a high level of organization and similarity. Why, then, is aqueous outflow confined to a few vessels under normal conditions?

Our study found that pulse amplitudes in the physiologic range can induce four μm excursions of the TM in ex vivo eyes. The excursion amplitude provides enough pulse-dependent stroke volume to account for aqueous flow (Li et al., 2012). However, the calculations incorporate the assumption that the motion of the entire circumference of SC contributes to the pulsatile discharge of aqueous.

#### 2.2.5. Circumferential flow – A normal phenomenon in vivo?

Conclusions that circumferential flow is negligible originated with observations in ex vivo eyes (Grant, 1958; Grant, 1963; Johnstone and Grant, 1973a) where ciliary muscle tension, blinking, and eye movements were absent. However, gonioscopy studies by Stegman in humans definitively demonstrate that aqueous moves circumferentially in SC for a considerable distance in synchrony with the oscillatory transients of the ocular pulse (Fig. 5) (Section (§) 4.1.2) (Vid 2). The absence of the in vivo factors in the original studies may resolve the apparent paradox.

We suggest that cyclic TM motion may induce circumferential flow, especially in young normal subjects. Corneal indentation occurs with blinking forcing aqueous into the AC angle. We can reason that corneal recoil introduces a relative vacuum effect on the TM, pulling it toward the AC, but the capture of such behavior is beyond the capabilities of current technologies.

Continuously recurring forces induced by both blinking and eye movement may increase the dynamic circumferential movement of aqueous within SC lumen, especially during waking hours. Rapid eye movement at night (REM sleep) may provide a means to partially counter the absence of blinking or eye movement during sleep. Although the constellation of clinical evidence appears consistent with the premise of transients inducing circumferential flow, the hypothesis requires further study.

#### 2.2.6. Clinical microsurgery insights into glaucoma causes

Results of minimally invasive glaucoma surgery (MIGS) in the 21st century necessitate questioning the idea that the TM is a primary source of resistance. MIGS procedures that either remove portions of the TM or bypass it typically achieve pressures in the mid-teens. However, if the TM were the primary resistance site, pressures near episcleral venous pressure would be achieved (Parikh et al., 2016), requiring an explanation that involves more distal pathways.

Krasnov developed a partial thickness non-penetrating procedure called sinusotomy in 1964 (Krasnov, 1964). The procedure removed the entire external wall of SC, leaving the internal TM wall of the canal intact. His published reports, including over 1000 surgeries, demonstrated excellent results in terms of clinical outcomes. Pre and postoperative tonography measurements provided credible evidence that the removal of SC external wall causes a marked improvement in aqueous outflow (Krasnov, 1969; Krasnov, 1969; Nesterov, 1970). Stegmann’s technique of viscocanalostomy and canaloplasty leave the TM in place with results as good as those with procedures removing the TM (Grieshaber et al., 2010, 2015).

Predictive operating room studies introduce a fluid wave into the AC after the Trabectome procedure. They then observe the resultant fluid wave in the aqueous and episcleral veins that is indicative of the functional properties of the distal pathways. Those with a vigorous fluid wave had lower postoperative IOPs implicating the distal pathways as a crucial factor in determining the ability to achieve low IOP after MIGS procedures (Fellman and Grover, 2014; Grover and Fellman, 2016). The findings point to the distal outflow pathways as an essential component of resistance.

### 2.3. Management of glaucoma – PhS-OCT

#### 2.3.1. Assessment of pump function – diagnosis, monitoring, management decisions

IOP measurements are inherently suboptimal in a clinical environment because of their random nature, inability to capture the range of ocular transients or diurnal variations, unknown levels of compliance, and brief duration (about sixteen seconds of thirty-one million seconds in a year). These limitations leave us with remarkably limited knowledge of the IOP profile in individual patients. In contrast, PhS-OCT captures the motion of the TM, the underlying biomechanical properties directly responsible for maintaining homeostasis.

Clinical aqueous vein and TM motion studies (via SC blood reflux) find evidence that pulsatile flow and TM motion progressively slow and then stop as glaucoma progresses. However, the examination techniques are difficult and time consuming for clinicians. PhS-OCT provides a noninvasive non-contact means of imaging and quantitating TM motion in humans (Fig. 6). The motion of the TM reflects TM tissue stiffness or elastance. Reduced elastance can play a vital role in explaining pathologic changes that lead to reduced TM motion, reduced pulsatile flow, and increased IOP in glaucoma (§5.2).

Knowledge of the TM biomechanical properties in individual patients may provide a means to predict who is having difficulty maintaining homeostasis and may provide an opportunity for earlier initiation or escalation of treatment. A recent report demonstrates the ability of PhS-OCT to distinguish normal from glaucomatous patients. Moreover, receiver-operator curves had high sensitivity and specificity in contrast to IOP and facility measurements that had an absence of predictive capability (Gao et al., 2019)abs.

#### 2.3.2. PhS-OCT – potential value in assessment of medication effectiveness

Patients not uncommonly have a limited or absent response to medications. Physicians must schedule a return visit soon after prescribing new medications to assess effectiveness, and IOP is the assessment tool (Allingham et al., 2012). Confounding variables hamper the assessment of medication effectiveness at the return visit. We often initiate or increase medical therapy on a visit when we identify an IOP elevation creating the likelihood of regression to the mean on the next office visit. Regression to the mean may lead to the erroneous conclusion that the medication is effective. Diurnal, day-to-day changes or poor adherence may result in presenting with elevated pressure even though the medication is effective (Allingham et al., 2012). Such confounding factors may result in patients remaining on an ineffective medication for months or years; alternatively, doctors may inadvertently abandon a medication even though it is effective in reducing IOP.

Adrenergics increase pulsatile flow markedly within 5 min, miotics in 5–20 min, and prostaglandins within 20–30 min, each medication resulting in a pressure drop within an hour (Ascher, 1961; Johnstone et al., 2011c). The ability to monitor TM motion with PhS-OCT provides the opportunity to measure baseline TM motion, instill drops, and then quantitatively assess whether a change in motion has occurred within an hour. A same-day office assessment with PhS-OCT may offer the opportunity to reduce medication response uncertainties. More importantly, it may reduce the number of people taking ineffective medication or not taking effective medication. Although knowledge of pulsatile flow responses to medications suggests PhS-OCT offers promise in improving the ability to manage medical therapy, more clinical studies are necessary to determine how it can fit into management decisions.

### 2.4. Therapeutics –restoration of pump function, aqueous flow, and IOP

#### 2.4.1. Outflow medications restore pump function and reduce IOP

Miotics increase pulsatile flow, a response which represents an increase in aqueous stroke volume that precedes a decrease in IOP. By temporarily restoring physiologic pulsatile flow in glaucoma patients, the drug’s effects on the outflow system offer insights into the outflow abnormality in glaucoma. The increased stroke volume results in a new lower equilibrium IOP setpoint that persists within the time frame of the drug’s duration. A full discussion of stroke volume determinates and concepts is in §3.8.4. The stroke volume increase precedes the pressure reduction to the new lower equilibrium setpoint (Ascher, 1942b; De Vries, 1947; Hodgson and Macdonald, 1954).

Multiple reports document the character of the pulse-dependent increase in stroke volume after introducing pilocarpine; for references, see (Johnstone et al., 2011c). Cambiaggi’s description typifies the increase in stroke volume found in reports. Pilocarpine induces a widening of the aqueous veins, an increase in current velocity, and a clearing of their contents that precede an IOP reduction. After an additional two hours, the clear aqueous current slows, and blood enters the vessel again from the episcleral vein’s direction, progressively moving toward the site of aqueous vein exit from the sclera (Cambiaggi, 1958).

Pilocarpine illustrates how medications may improve stroke volume. The underlying problem may be abnormal elastance resulting from loss of the ciliary body ability to maintain optimal tensile loading of the TM lamellae. Pilocarpine causes the ciliary muscle to contract, placing an increased load on the TM tendons. The increased tensile load may restore the TM lamellae’s normal elastance, and through an improved stroke volume, transiently restore normal IOP. Another aspect of the restoration of elastance may be the result of the ciliary muscle pulling the TM away from an abnormal apposition to SC, which allows normal elastance-dependent TM distention and recoil to occur.

The water-drinking test that increases IOP mirrors the effects of pilocarpine. Water drinking causes a temporary increase in IOP, leading to an increase in pulse amplitude and aqueous outflow velocity. The increased pulsatile aqueous outflow then restores pressure to the baseline IOP homeostatic setpoint (Johnstone, 2004) (Fig. 3), at which time the increased pulsation amplitudes also return to baseline. Adrenergic agents also produce a stroke volume increase, followed by an IOP reduction to a new homeostatic setpoint (Goldmann, 1946b; De Vries, 1947; Ascher, 1961; Johnstone, 2006). Prostaglandins provide a similar increase in pulsatile flow, followed by a reduction in IOP to a new lower setpoint (Johnstone et al., 2007, 2008)abs. Outflow medications increase the stroke volume of aqueous, which precedes the reduction of IOP.

#### 2.4.2. Cataracts change ciliary muscle tension and vector force

**Fig. 7.**
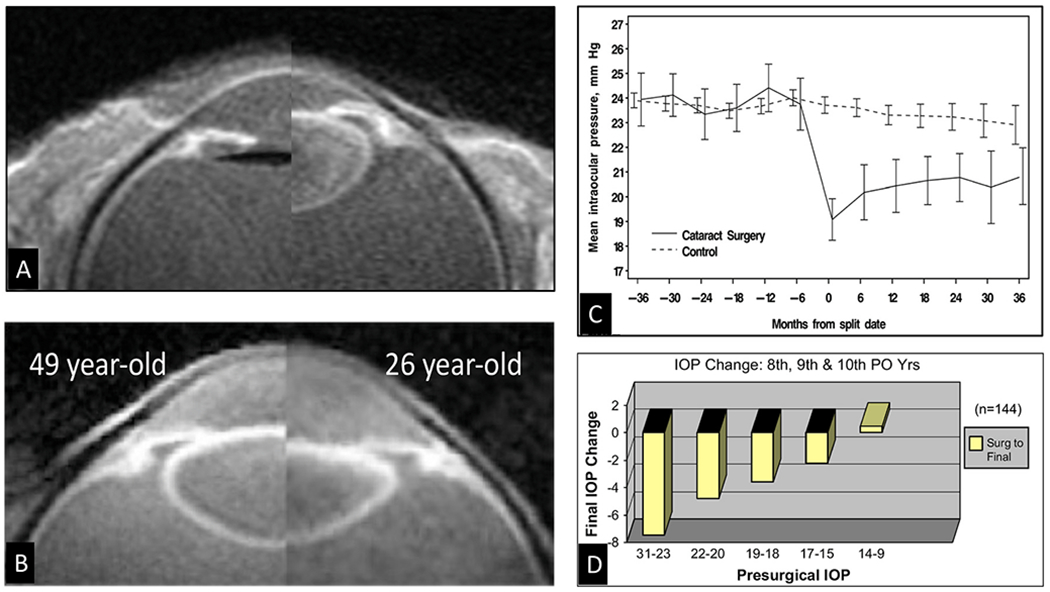
Cataract surgery changes outflow system vector forces, favoring improved outflow. **(A)** High-resolution MRI of a 74-year-old. The crystalline lens is present in the right image, but in the fellow eye of the left image, an artificial lens replaces the crystalline lens. Note the posterior shift of the ciliary body after cataract surgery. The haptic is perpendicular to the image and appears black. **(B)** In vivo composite image showing that life-long lens growth displaces the uvea anteriorly. Backward movement of the ciliary body induces vector forces pulling the scleral spur both posteriorly and inward. **(C)** Intraocular pressure (IOP) before and after cataract surgery in the Ocular Hypertension Treatment Study. Month 0 is the study visit that the participant reported cataract surgery, or a randomly selected, corresponding date in the control group. Error bars are ± two standard errors of the mean. **(D)** Final IOP changes in combined postoperative years following cataract surgery grouped by presurgical IOP (PO is postoperative) (The 8th, 9th, and 10th year pooled data represents the 144 patients.) **(A)**, **(B)** and **(D)** from Poley B, IOP after cataract surgery in open-angle glaucoma. J Cataract Refract Surg 35,1946–1955, 2009, **(C)** from Mansberger S, IOP after cataract surgery, Ophthalmology 119, 1826–1831, 2012.

Continuous age-dependent lens growth shallows the AC (Fig. 7). The anterior lens surface moves forward, and the zonules attached to it move forward as well. The resulting series of age-related events results in chronic traction on the ciliary muscle, causing it to move anteriorly. The combined effects cause repositioning of the anterior lens capsule and zonules to be in front of SC. The high-resolution MRI images of Strenk and Strenk illustrate the age-dependent anterior movement of the ciliary muscle (Fig. 7) (Poley et al., 2009; Strenk et al., 2010, 2018).

The resulting anterior ciliary muscle repositioning decreases traction on the attached trabecular tendons resulting in 1) a decreased TM interspaces area, 2) a decreased separation between the TM and SC external wall, 3) a decreased range of distension necessary to fulfill the sensory baroreceptor function, 4) a decreased tension on SC outlet valves, 5) a decreased end-diastolic volume, 6) a decrease in TM pulse-dependent motion, 7) a decrease in circumferential flow (Van Buskirk and Grant, 1973; Van Buskirk, 1976, 1982; Li et al., 2012) (§3.2).

After cataract surgeiy, the AC deepens, and the lens capsule position is behind SC so that the zonules no longer pull the ciliary muscle forward. The resulting posteriorly directed vector forces now allow the ciliary muscle tension to rotate TM and scleral spur attachments backward, increasing the tension on the TM tendons. The configuration change allows both the TM interspaces and SC to enlarge, as a recent OCT study demonstrates (Zhao et al., 2016). Moreover, the procedure does not result in the invasive structural damage to the outflow apparatus associated with other MIGS procedures.

Pilocarpine is a drug that causes ciliary muscle contraction and temporarily restores normal pulsatile flow with associated reduction of IOP. The effects of cataract surgery on the ciliary muscle are like those of pilocarpine (§2.4.1). The OHTS study reports that removal of the crystalline lens in cataract surgery decreases postoperative IOP by > 4.0 mm Hg (16%) (Mansberger et al., 2012). Clinical evidence indicates that cataract surgery reduces IOP (Masis Solano and Lin, 2018) and that the IOP reduction can persist long-term (Fig. 7).

#### 2.4.3. Micropulse laser – ciliary muscle tightens – effects on TM, SC, CC

A recent study explored the hypothesis that the reduced energy of the micropulse transscleral laser can cause modest heat-induced ciliary muscle shrinkage that mimics the pilocarpine effect (See Video in §6.0). Radial sections of the limbus 2–3 mm in thickness were prepared from primate eyes obtained immediately after death. Transscleral laser application to the ciliary muscle used a probe placed on the sclera as is done clinically. Real-time video during the laser application demonstrated a localized shrinkage of the longitudinal portion of the ciliary muscle. There was an associated statistically significant backward movement of the scleral spur and enlargement of SC (Johnstone et al., 2019)abs. Damage to the secretory epithelium of the pars plicata was absent. The ability of micropulse transscleral laser to alter the configuration of the outflow channels like pilocarpine suggests that with further optimization of parameters, technological efforts might lead to a procedure that provides persistent long-term IOP-reducing benefit without the troublesome side effects of pilocarpine.

## 3. Laboratory science: outflow system structure, function, failure in glaucoma

Clinical studies establish the presence of an outflow apparatus that acts as an organ system inducing pulsatile aqueous outflow behavior like that in the lymphatics and veins. The pulsatile behavior indicates the need to identify the outflow system structures that act as the pump-conduit system’s moving components. In the laboratory science section, we consider the outflow pathway structures from the perspective of their ability to explain and predict pulsatile aqueous outflow. The perspective imbues each of the individual outflow system components with new roles and importance.

### 3.1. The trabecular meshwork – mobile wall of a compressible chamber

#### 3.1.1. Aqueous flows into a vessel (SC lumen) – outflow system modifications

**Fig. 8.**
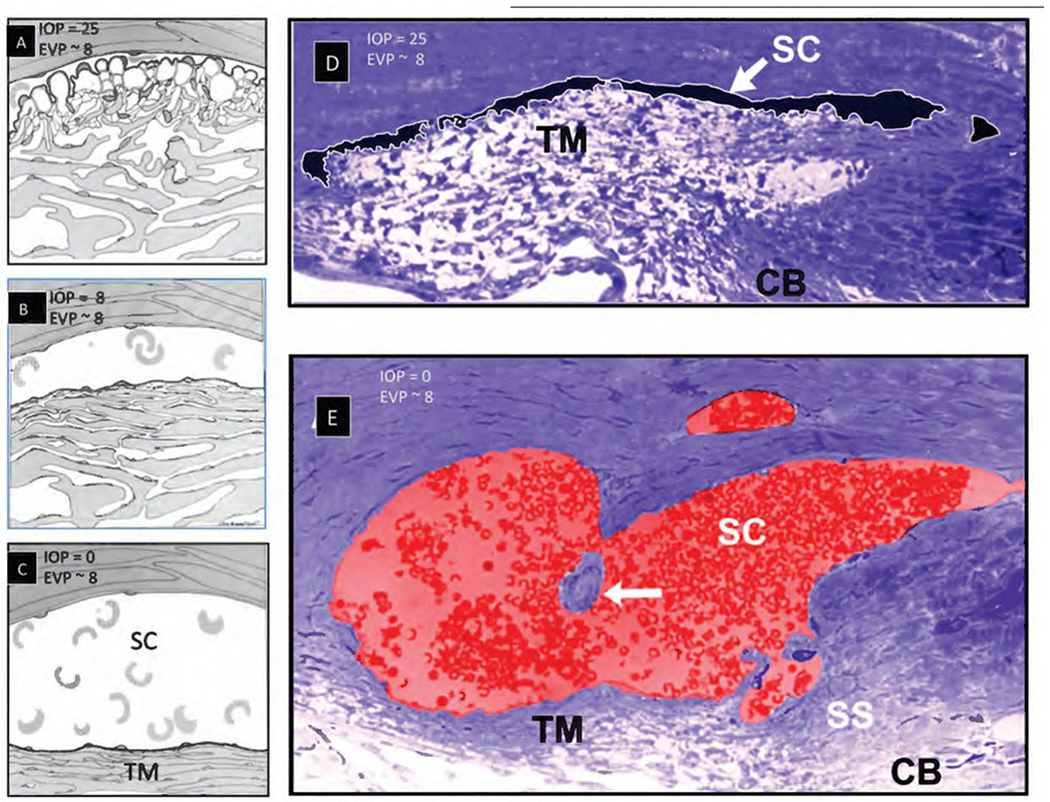
Pressure-dependent configuration of the trabecular meshwork, and scleral spur. Images **(A)**, **(B)**, **(C)** depict the tissue configuration with intraocular pressure (IOP) & episcleral venous pressure (EVP) as indicated. **(D)** Primate eye fixed in vivo at IOP of 25 mm Hg and normal EVP. **(E)** Fellow eye of **(D)** fixed in vivo with an IOP of 0 mm Hg and normal EVP. White arrow in **(E)** indicates the SC inlet valve suspended in the canal with the valve connections to the TM and external wall identified in serial sections. These experiments were the first to recognize IOP as the cause of “giant vacuoles” that deform not only SC inner wall endothelial cells but also the trabecular meshwork (TM) lamellae. The scleral spur (SS) and ciliary body (CB) undergo marked changes in configuration, moving outward as IOP increases. Cellular connections, depicted in **(A)** between the TM, juxtaeanalieular cells, and SC endothelium, provide tethering of SC inner wall to prevent it from collapsing into SC. Tissue source of (D) and (E): Primate, *Macaca mulatto.* Adapted from Johnstone M and Grant M, Pressure-dependent changes in structure of the aqueous outflow system. Am. J. Ophthalmol 75, 365–383, 1973.

**Fig. 9.**
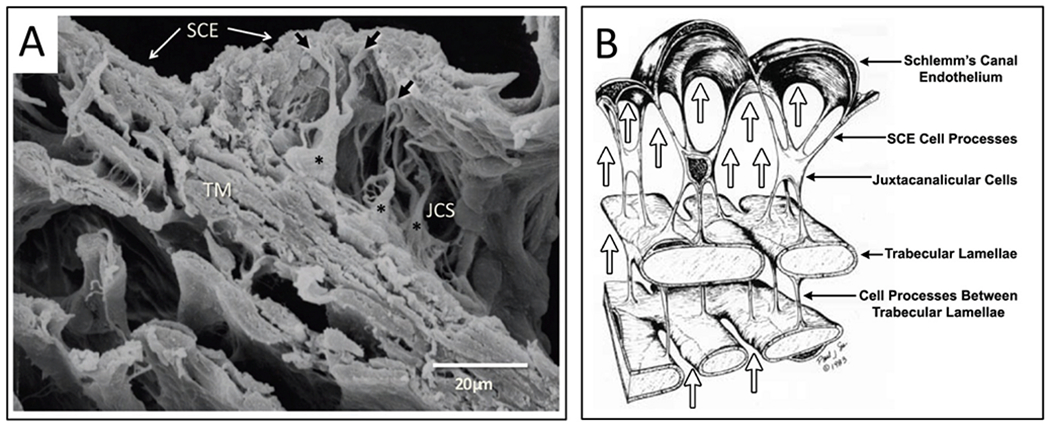
Connections attach the Schlemm’s inner wall to the trabecular lamellae. **(A)** Cytoplasmic processes (black arrows) of juxtaeanalieular cells (asterisks) in the juxtaeanalieular space (JCS) link Schlemm’s canal endothelium (SCE) to the underlying trabecular beams of the trabecular meshwork (TM). **(B)** Arrows depict the source of the pressure gradient originating from the anterior chamber (AC). SCE cell cytoplasmic processes attach to juxtaeanalieular cell processes. Juxtaeanalieular cell processes also attach to cytoplasmic processes arising from the endothelium covering the trabecular lamellae. This arrangement provides a mechanism anchoring the inner wall endothelium of Schlemm’s canal to the lamellae. Intertrabecular cytoplasmic processes also maintain contact between adjacent lamellae. The continuous pressure gradient between the AC and SC permits the cytoplasmic attachments to tensionally integrate the structural elements of the trabecular meshwork, providing tissue and cellular prestress. **(A)** Tissue source: Human. From Johnstone M. The aqueous outflow system as a mechanical pump: evidence from examination of tissue and aqueous movement in human and non-human primates. From: **(A)** J Glaucoma 13, 421–438.

In the systemic vasculature, pressures inside the lumen are higher than those outside, favoring lumen enlargement. In contrast, the lumen of SC experiences pressure gradients outside its inner TM wall favoring lumen closure. The outflow system requires unique adaptations to ensure that the SC lumen remains patent despite the constant IOP force pushing SC inner wall outward into the canal lumen. The adaptation to prevent SC lumen closure involves numerous endothelial cell cytoplasmic processes that tether the TM lamellae, juxtacanalicular cells, SC endothelium, and SC inner wall together to provide a tensionally integrated structure. The tethering system is central to the aqueous outflow pump model and its ability to sense and control IOP (Figs. 8 and 9) (§5.1).

Evidence from biomechanics studies demonstrates that cytoplasmic processes connect all the structural elements of the TM, permitting the cytoplasmic processes to provide spring-loaded cellular connections (§5.2) that control TM excursions into SC (Fig. 9) (Johnstone and Grant, 1973a). SC endothelium moves outward in response to mean pressure, oscillatory forces, and the frequent transient pressure spikes that narrow the canal lumen. Wet lab studies in fresh tissue (§3.13) indicate that a pulse wave, like that occurring during systole, will induce an outward movement of the tissues and fluid containing spaces. Together, the elastance properties of the TM lamellae, juxtacanalicular, and SC endothelial cells can modulate the motion.

The cytoplasmic processes of adjacent TM lamellae join one another in the intertrabecular spaces. The surfaces of the TM lamellae facing the JCT space also have abundant processes that attach to juxtacanalicular cell processes (Fig. 9) (Holmberg, 1965; Hogan et al., 1971; Inomata et al., 1972; Johnstone and Grant, 1973a; Grierson and Lee, 1974b, 1975a; Grierson et al., 1978; Johnstone, 1979). Robust desmosomes join cytoplasmic processes together and confer the ability to withstand the forces associated with pressure changes (Grierson and Lee, 1974b; Grierson et al., 1978; Desai et al., 2009). The cytoskeletal network of the TM, juxtacanalicular, and SC endothelial cells and their respective cytoplasmic processes bear the IOP load and provide the ability to distend and recoil.

The tethering of SC endothelium to TM lamellae via cytoplasmic processes ensures that when the TM moves outward into the SC lumen, the lamellae can limit the sheet of SC endothelial cells distention into the canal. Similarly, when pressure decreases, the same tethering mechanism engages the TM lamellae to induce a recoil response that enlarges SC. The concept of cellular connections proposed here contrasts with the hypothesis that ECM material primarily determines TM relationships and responses to pressure changes (Vahabikashi et al., 2019).

A well-defined basement membrane anchors TM lamellae endothelial cells to the ECM of the lamellae (Tervo et al., 1995; Zhou et al., 1996, 1999). In contrast, SC inner wall endothelium has a sparse or absent basement membrane described as “poorly defined, inconstant, frequently interrupted, and of variable thickness” (Hogan et al., 1971). Although the basement membrane has discontinuous regions, the membrane is complex, and its composition may provide it with a role in outflow regulation (Acott et al., 2020).

The connections between the SC endothelium and TM lamellae serve to anchor SC inner wall endothelium to the basement membrane of TM lamellae. The tethering attachments limit distention into SC. In contrast, the pressure inside the lumen of other vessels forces the cells outward against a basement membrane. The SC inner wall connections permit communication of SC inner wall endothelial stresses directly to the basement membranes of the endothelial cells investing the TM lamellae (Fig. 9). The anchoring mechanism provides one means to explain why SC inner wall endothelium can have a minimal or absent basement membrane.

#### 3.1.2. The TM lamellae - mobile determinates of SC volume

**Fig. 10.**
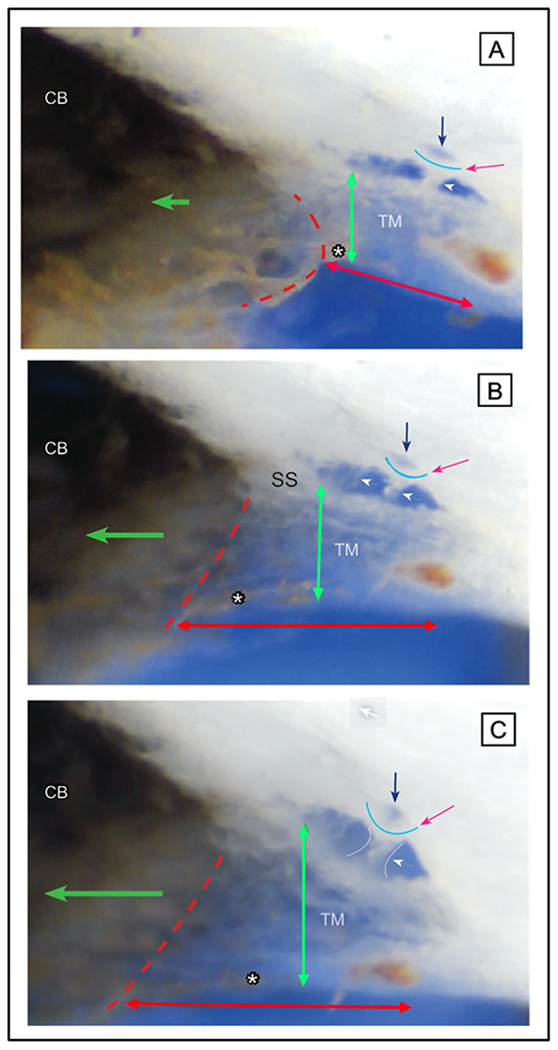
Outflow system response to ciliary muscle tension: Motion observations. Ciliary body tension on outflow pathways in a radial limbal segment (~2 mm thickness). Forceps tension moves the ciliary body (CB) posteriorly to simulate ciliary muscle contraction on the scleral spur (SS), and trabecular meshwork (TM). Green arrows indicate A) no CB tension, B) moderate CB tension, and C) increased CB tension. Concurrent phenomena demonstrate a highly interconnected system with structures moving in synchrony. As tension increases, the CB-TM tendon interface moves posteriorly, (dotted red outline). The scleral spur also rotates posteriorly and inward toward the AC. TM lamellae attached to both the scleral spur and the ciliary body move posteriorly and elongate (double-headed red arrows). The TM lamellae are anchored anteriorly to Schwalbe’s line. The lamellae move inward with a fan-like motion enlarging the more posterior intertrabecular spaces with minimal movement near SL (double-headed green arrows). Intertrabecular spaces between the parallel layers of the TM lamellae communicate directly with the face of the ciliary muscle and the TM-CB tendons. Oscillatory TM lamellar excursions, more substantial in the posterior than anterior spaces of the lamellae, will ensure constant oscillatory aqueous movement toward and away from the ciliary tendons and muscle. Translucent cylindrical structures, the SC inlet valves, arise from SC inner wall and cross SC (white arrowheads) to a septum at the SC external wall (red arrow). As CB tension increases, the septum moves toward SC, becoming progressively more curved (green arrow). The septum movement enlarges the lumen of a circumferentially oriented deep scleral plexus (CDSP) (black arrow). The release of forceps tension results in a rapid recoil from the configuration in C to that of A. Ciliary body tendinous attachments to the trabecular lamellae cause them to elongate and remain in a constant state of tensional prestress in vivo. Tension on the SC inlet valves also places tension on the septa and flaps at CDSP. The ciliary body tension maintains prestress of trabecular lamellae, SC inlet valves and septa. The prestress determines the lumen size of SC, CC, and CDSP. The optimized stresses and lumen size permit the TM to distend and recoil, enabling changes in stroke volume in response to ocular transients. Normal ciliary muscle tone and loading forces are absent in ex vivo preparations. – Tissue source: primate, *Macaca nemestina.* From Johnstone M, Intraocular pressure control through linked trabecular meshwork and collector channel motion. Glaucoma Research and Clinical Advances: 2016 to 2018. Kugler Publications, Amsterdam 2016. Ciliary Muscle Effect on Outflow Pathways 1-s2.0-S1350946220300896-mmc6.mp4.

**Fig. 11.**
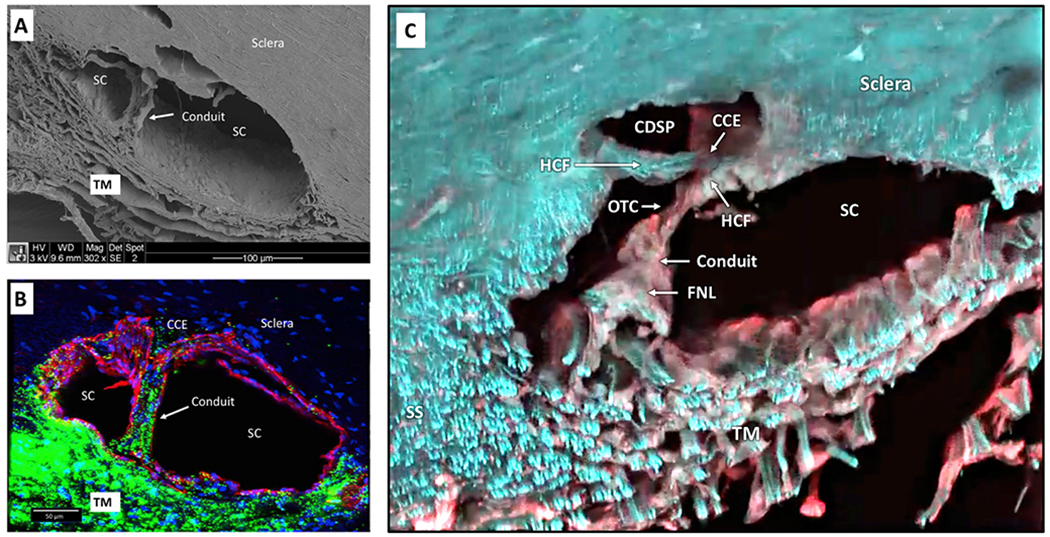
Conduits cross Schlemm’s canal to hinged collector channel flaps. **(A)** Scanning electron microscopy image after Schlemm’s canal (SC) dilation with viscoelastic and fixation. The inner wall of SC rests against several layers of adjacent collapsed trabecular meshwork (TM) lamellae. Nuclear bulges of endothelial cells are visible along the SC inner wall. A Schlemm’s canal inlet valve arises from SC inner wall endothelium and crosses SC to the external wall. The lumen of the inlet valve is continuous with the juxtacanalicular space of the TM. The SC inlet valve lumen thus provides a conduit for aqueous passage from the juxtacanalicular space to SC. **(B)** Anterior segment of the eye perfused with 500 nm green fluorescent microspheres followed by Schlemm’s canal dilation with viscoelastic. Confocal microscopy labeling is with DAPI (blue) for nuclei and CD31 (red) for vascular endothelium. The CD31 label identifies continuity of SC inner wall and SC inlet valve endothelial surfaces. Microspheres fill the intertrabecular spaces. A Schlemm’s canal inlet valve labeled with CD31 originates from SC inner wall endothelium and crosses SC to a collector channel entrance (CCE). Microspheres fill the SC inlet valve lumen from its origin at SC inner wall to its attachment at a CCE at SC external wall. Passage of microspheres provides evidence that the SC inlet valves can function as a conduit for aqueous. **(C)** Confocal microscopy - native fluorescence, SC viscoelastic dilation. An SC inlet valve initially forms a funnel (FNL) arising from the TM, develops a cylindrical conduit area, then attaches to the tip of a hinged collagen flap (HCF) at SC external wall. A Schlemm’ canal inlet valve has a direct opening to SC (OTC). The OTC is adjacent to a CCE at the entrance to a circumferential deep scleral plexus (CDSP). Tissue source: Primates, *Macaca nemestrina.* From Johnstone Glaucoma Lab, University of Washington.

**Fig. 12.**
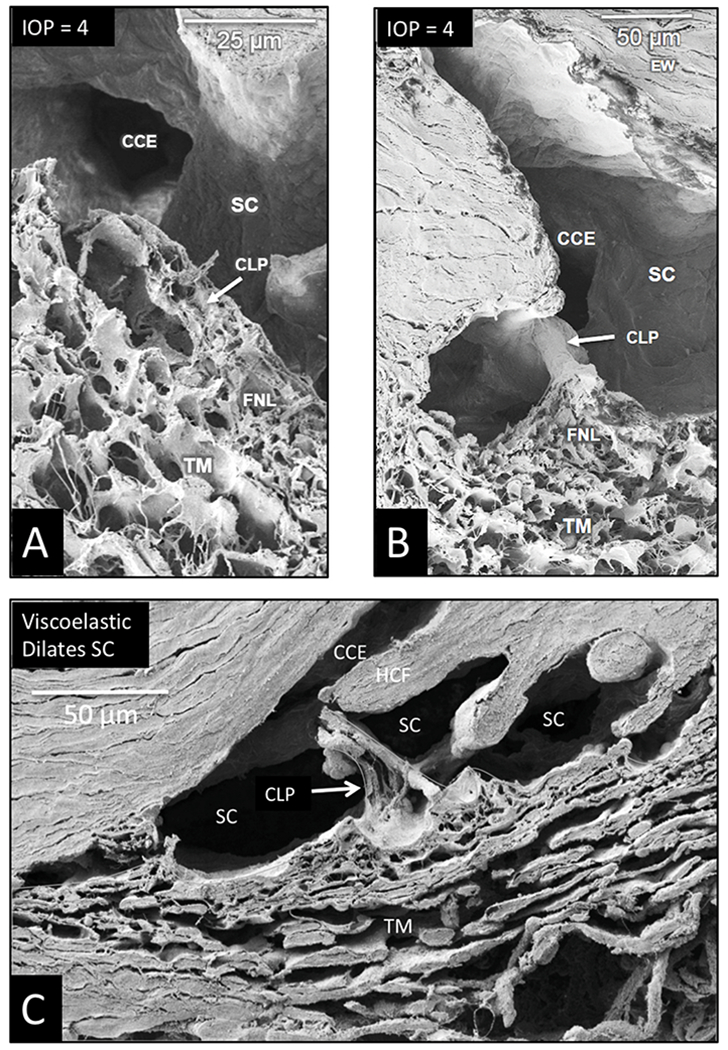
Schlemm’s canal inlet valve attachments to SC external wall at collector channel entrances. Scanning electron microscopy images **(A, B)** are from an eye with an IOP of 4 mm Hg during fixation. An SC inlet valve has a funnel shaped (FNL) region as it joins the trabecular meshwork (TM). The funnel region leads to a cylindrical area that crosses SC to attach at collector channel entrances (CCE). The section in **(A)** bisects the SC inlet valve lumen of the conduit-like pathway (CLP), revealing the lumen connection between the funnel-shaped juxtaeanalieular space and collector channel entrance. Internal structural features are like those of the juxtaeanalieular space. **(B)** A view of the funnel region of a Schlemm’s inlet valve with a plane of section revealing its surface features as it courses across the canal from the TM to SC external wall at the entrance of a collector channel. **(C)** Viscoelastic dilated Schlemm’s canal (SC) before fixation. The image reveals the conduit-like pathway (CLP) of the Schlemm’s canal inlet valve as it arises from the TM and attaches to a hinged collagen flap (HCF) at SC external wall. The inlet valve is bisected, revealing an open distal end in communication with a collector channel entrance (CCE). Aqueous can flow freely into SC and CCE through the lumen of the conduit-like pathways. Tissue source: Primate, *Macaca nemestrina.* From the Johnstone Glaucoma Lab, University of Washington.

The corneoscleral meshwork consists of 5–12 μm thick parallel collagen lamellae or beams, 8–14 in number (Flocks, 1957). The ciliary muscle’s geometry and contractile properties have a marked effect on the TM lamellae configuration (Fig. 10). Tendons join the TM lamellae and ciliary muscle. TM lamellae closer to the AC are considerably thicker than those close to SC (Fig. 8) (Flocks, 1957). Schwalbe’s line anchors the anterior border of these larger lamellae sheets. The lamellae extend posteriorly in a meridional fashion to attach to the scleral spur and ciliary muscle (Hogan et al., 1971). The TM lamellae sheets’ arrangement is generally circumferential with the sheets parallel to one another and the limbal circumference (Flocks, 1957) (Figs. 10–12). Only two or three layers of TM lamellae are present anteriorly because the sheets fuse at their anterior attachment. Because the sheets separate in an anterior-posterior plane, 12–20 layers are detectable posteriorly. Openings or perforations in the TM lamellae have an elliptical orientation and are 12–30 μm in diameter (Flocks, 1957). Aqueous humor must follow a circuitous route to reach SC because neither space between lamellae nor perforations through them align in views seen in radially oriented histologic sections (Flocks, 1957). Essential to understanding outflow dynamics is the fan-shaped meridional configuration of the TM lamellae. The resultant accordion-like geometric relationships govern and constrain pressure-dependent excursions of the TM to an asymmetric pressure-dependent motion (Fig. 10).

##### 3.1.2.1. TM Motion Video.

(Vid 4) 1-s2.0-S1350946220300896-mmc4.mp4). The asymmetry of TM excursions necessarily forces aqueous not only between the TM lamellae toward SC but because of vector forces, must also induce cyclic aqueous movement toward and away from the ciliary muscle in response to oscillatory and other transients. Moving from the layers of the corneoscleral meshwork nearest the AC to those closer to SC, the TM lamellae’s perforations become progressively smaller, but the smaller lamellae have large spaces between each other (Johnstone and Grant, 1973a) (Fig. 8).

#### 3.1.3. Dissecting microscope: real-time imaging of meshwork distention and recoil

We can directly observe pressure-dependent TM distension and spontaneous recoil using real-time videomicroscopy in unfixed ex vivo eyes with calibration provided by a micrometer scale (Johnstone et al., 2014a)abs (Vid 4). The simple technology permits observation and quantitative analysis of a same-sample time series in fresh tissue. Technique details are in §4.2.

As an example, a single pulse wave of balanced salt solution introduced from a cannula can cause the TM lamellae to move outward, causing SC height to decrease rapidly, resulting in regions of SC appositional closure. When infusion stops, the TM tissues rapidly recoil to their prior dimensions. TM lamellae distension and recoil are the primary components of the TM motion. The real-time video provides a simple, directly observable biologic behavior that demonstrates the TM lamellae’s role in pressure-dependent dimension changes of SC.

#### 3.1.4. Trabecular lamellae composition explains the ability to distend and recoil

The organization and distribution of collagen and elastin in the TM lamellae is like that of a tendon (Hernandez and Gong, 1996); such tissue properties explain reversible deformation in response to pressure-dependent tissue loading of the TM (Johnstone and Grant, 1973a; Grierson and Lee, 1975a; Hariri et al., 2014; Xin et al., 2017). Collagenous components provide structural support in tension, while elastin ensures a recoverable response over a wide range of excursions (Humphrey, 2002). Composition of the corneoscleral lamellae compares to other organ systems with marked elasticity and compliance, such as the lung and blood vessel walls (Gattinoni et al., 2004; Suki et al., 2005). The composition of the TM lamellae determines their elastance that provides the ability to store and release energy, a property that allows them to distend and recoil in response to pulse transients. OCT TM Motion Video (Vid 5) 1-s2.0-S1350946220300896-mmc5.mp4.

#### 3.1.5. Trabecular lamellae elastance/stiffness – key to pump function and failure

The trabecular lamellae undergo large excursions in response to pressure changes and are the primary determinates of bulk TM motion. The lamellae distend into SC when IOP increases, reducing SC lumen size and recoil when pressure falls. The TM lamellae are the structures of the outflow system that undergo most of the motion that drives pulsatile aqueous outflow, so their stiffness is of unique relevance to the issue of glaucoma.

#### 3.1.6. Endothelial cells regulate trabecular lamellae elastance

With high-resolution OCT, we track regional changes in the SC dimensions in response to pressure changes, from which we develop trabecular meshwork elastance curves. Such curves reflect bulk TM stiffness (Xin et al., 2016). A finite element approach that used data from spectral-domain OCT (SD-OCT) studies of TM motion determined bulk TM stiffness and found that the outflow facility was associated with TM stiffness in both healthy and glaucomatous eyes (Wang et al., 2017). The elastic modulus of normal human TM estimated by inverse FEM was 70 ± 20 kPa (mean ± SD), whereas glaucomatous human TM was stiffer (98 ± 19 kPa).

The endothelial cells investing the lamellae are crucial to their function because they regulate the highly organized composition of the lamellae ECM that determines their biomechanical properties. As with vessel walls in the systemic circulation, the tissue composition of SC’s trabecular wall determines responses to mean pressure and oscillatory tissue loading (Fung and Liu, 1993; Fung, 1996; Humphrey, 2002). The constituent composition that determines elastance includes type 1 collagen that provides tensile strength, type III collagen that imparts resilience, and elastin that permits a recoverable response after deformation.

Appropriate motion responses of the lamellae must remain in a narrow range to regulate short-term and long-term homeostasis (§5). The TM lamellae cells must continually transduce pressures and flow-dependent sensory inputs. Such sensory signals are necessary to maintain the TM lamellae ECM’s highly organized geometric relationships, structural composition, and biomechanical properties. As a result of pulse waves impinging on the TM lamellae and their tethering cytoplasmic attachments, the cells covering the TM lamellae experience continuous oscillatory and transient wall stresses (Fig. 9) (See also §5.2.4).

One might conclude that flow-dependent shear stress within the TM lamellae is unlikely if the aqueous flowed passively through the TM lamellae, and the lamellae did not move. However, the TM lamellae experience large excursions and move at speeds measured in milliseconds in response to normal oscillatory pressures and transients (§2.3). The TM lamellae consist of sheets of meridionally-oriented beams with an asymmetric fan-shaped configuration. Because of offset openings in adjacent sheets, aqueous must wend its way within the interspaces between sheets to the offset openings as well as moving anteriorly and posteriorly within the fan-shaped lamellae. The tissue geometry and recent evidence of rapidity of motion lead us to hypothesize that the TM lamellae experience flow-induced shear stress with each pulse wave.

#### 3.1.7. Juxtacanalicular space – tensional integration and sensory functions

The juxtacanalicular space is the area between the outer layers of the corneoscleral meshwork and the inner wall of SC (Figs. 2, 8 and 9). Terms for the space include juxtacanalicular tissue and cribriform region favored by those describing the passive flow model. The term cribriform is defined as “pierced with small holes like a sieve.” Names to describe the region include juxtacanalicular space, subendothelial space, pericanalicular space, juxtacanalicular area, and juxtacanalicular region. Aqueous forced through a tissue sieve by pressure is likely to be conceptualized quite differently from aqueous flow through spaces. In the aqueous outflow pump model, we prefer to use terms denoting space through which aqueous can flow freely because such terms do not have the concept of a passive filter embedded in them. Cells in the space have been called cribriform cells, subendothelial cells, and juxtacanalicular cells.

Juxtacanalicular cells and their cytoplasmic processes provide an anchoring mechanism for SC endothelial cells to attach to the TM lamellae. The anchoring provides the SC endothelium with a mechanical role in maintaining a tensionally integrated system and a sensory capability (§5). An elastic tendon system connected to the ciliary muscle also plays an essential synergistic role in maintaining tensional integration. When eyes are pressurized to normal levels, three-dimensional views provided by scanning electron microscopy (SEM) (Fig. 9) and transmission electron microscopy (TEM) reveal that juxtacanalicular cells and their cytoplasmic processes are the principal features of the region (Johnstone and Grant, 1973a; Grierson and Lee, 1975a). Cytoplasmic connections between SC endothelium, juxtacanalicular cells, and TM lamellae link all the cells of the respective structures together (Holmberg, 1965; Hogan et al., 1971; Inomata et al., 1972; Johnstone and Grant, 1973a; Grierson and Lee, 1974b, 1975a; Grierson et al., 1978; Johnstone, 1979).

As IOP increases, multiple signs of cellular stresses indicate a cellular-based system of tensional integration. In response to an IOP increase, the juxtacanalicular space enlarges, cytoplasmic processes undergo rearrangement from a parallel to a perpendicular orientation, cytoplasmic processes both elongate and thin. The cytoplasm and nuclei of juxtacanalicular cells and SC cells deform at cytoplasmic process origins giving the juxtacanalicular cells a stellate appearance and causing elongation of the cytoplasm and nucleus of SC endothelial cells (§5) (Johnstone and Grant, 1973a; Grierson and Lee, 1974b, 1975a; Grierson et al., 1978; Johnstone, 1979, 1984, 2004).

**Fig. 13.**
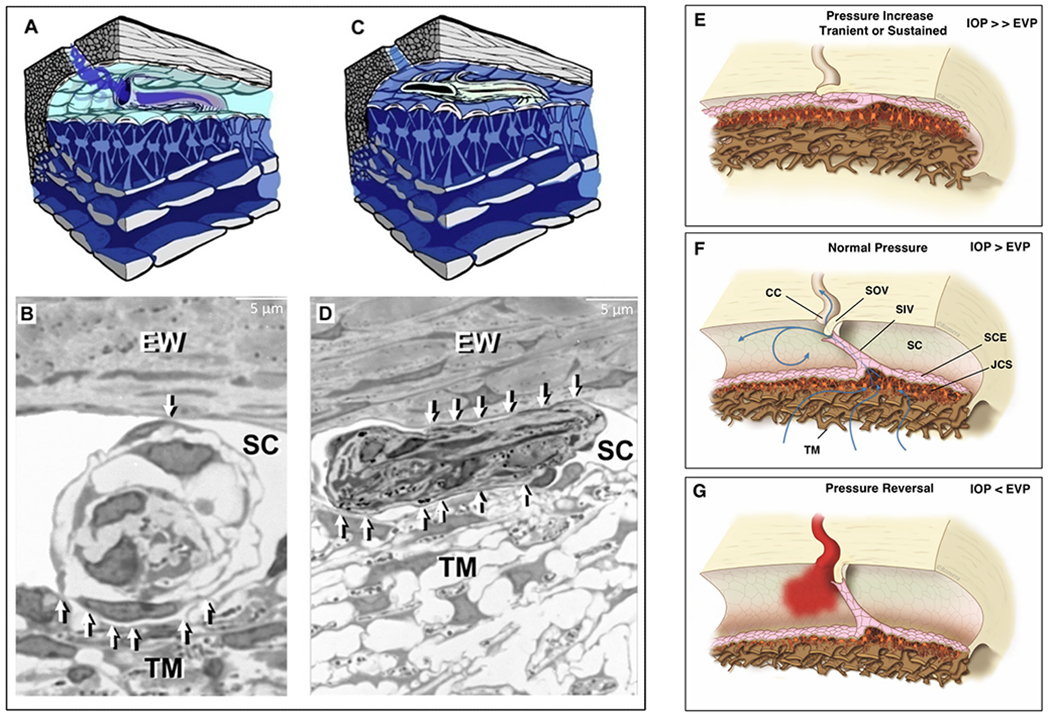
Schlemm’s canal inlet valves – transient compression between Schlemm’s canal walls. Left panel. Following in vivo fixation at 25 mm Hg, radial serial sections track a Schlemm’s (SC) inlet valve as it courses circumferentially in SC over a distance of 70 μm. (A) Illustration of Schlemm’s canal inlet valve discharging aqueous into the canal adjacent to a collector channel. **(B)** Compression of the same valve in regions of its course along SC. The pressure-dependent trabecular meshwork (TM) comes into proximity with the SC external wall causing varying degrees of compression and valve lumen closure throughout the valve length. The lumen of the inlet valve is at times open as in (**A-B)**, and other times closed as in **(C–D)**. White arrows identify areas of compression. Frequent closure of SC lumen is an expected finding in vivo as a result of pressures caused by normal ocular transients (Section 3.8.2) Tissue source: Primate, *Macaca mulatta*. Right Panel **(1)** Transient IOP increase from blinking, eye movement, lid squeezing, or experimental in vivo steady-state IOP of 25 mm Hg results in the configuration of Panel A **(C–D)**. A TM elastance abnormality may result in persistent SC closure and elevated IOP. **(2)** Pump-conduit configuration at homeostatic IOP. **(3)** Pressure reversal results in SC dilation and blood reflux. IOP: Intraocular Pressure, EVP: Episcleral Venous Pressure, SC: Schlemm’s Canal, SCE: SC Endothelium, TM: Trabecular Meshwork, CC: Collector Channel, JCS: Juxtacanalicular Space, SIV: SC Inlet Valve, SOV: SC Outlet Valve. Left Panel from Poley B J Cataract Refract Surg 35, 1946–1955, 2009. Right Panel by Antonio Valladares and Manuel Romera. Video – TM Motion Closes SC 1-s2.0-S1350946220300896-mmc4.mp4.

When the sheet of SC inner wall endothelium distends in response to a pressure increase, the extensive array of tethering cytoplasmic process attachments necessitates the TM lamellae’s synchronous distention into the canal (Figs. 1, 13, 15 and 16). and §5. Tethering to TM lamellae prevents SC inner wall endothelium from being forced against SC external wall.

Moreover, the tethering mechanism permits SC inner wall endothelial cell nuclei and cytoplasm to deform. Since there is little resistance to flow in the interspaces of the lamellae, IOP should not cause the lamellae to distend unless tethered to SC inner wall endothelium. Cytoplasmic processes tethering of the TM lamellae to SC endothelium explains the steady-state TM lamellae distention in response to pressure. The elastic fiber system can play a synergistic role in the tethering relationship.

#### 3.1.8. Mean IOP vs. oscillatory responses

Maintenance of optimized outflow pump function requires the regulation of two TM mobility-dependent properties. The first property is the regulation of the mean steady-state pressure-dependent relationship between the TM and SC external wall. The second property is the ability to respond to cyclic and transient pressures that cause the TM to oscillate around its mean steady-state configuration; the latter property permits the TM to change SC dimensions allowing the canal to act as a dynamically compressible chamber.

How does the TM respond to an increase in IOP? In response to an IOP increase, the intertrabecular space enlarges, the juxtacanalicular space enlarges, and SC inner wall endothelium distends into SC (Johnstone and Grant, 1973a; Grierson and Lee, 1975a; Van Buskirk, 1982). TM distension into SC is highly dependent on ciliary muscle tone that determines TM loading forces and TM stiffness, as noted in §3.2.

How does the TM respond to IOP oscillations and transients? Wet lab studies in fresh tissue provide some clues §3.1.3, Vid4. The TM can cause SC lumen height reduction from 85 to 16 μm in 586 ms with spontaneous recoil to the original configuration in 526 ms. The movement results from the bulk motion of the TM lamellae. OCT imaging demonstrates ~3.5 μm oscillations in response to 3 mm pulse amplitudes in ex vivo primate eyes. TM responses to both cyclic oscillations and transients are crucial elements of function and warrant further study.

### 3.2. Ciliary Muscle-TM Lamellae – an inseparable regulatory unit

Studies point out that the TM and ciliary muscle are an inseparable regulatory unit (Wiederholt et al., 2000; Kaufman, 2020). Genes implicated in glaucoma, including MYOC, FOXC1, PITX2, CYP1B, are expressed at high levels within the ciliary muscle. Although these genes are present in the TM, their strong expression in the ciliary muscle suggests that TM expression alone may not provide a full explanation for their role in the glaucoma process (Tamm, 2002; Van Zyl et al., 2020). In both the ciliary muscle and the TM, ECM material increases with age (Gabelt and Kaufman, 2005).

The ciliary muscle is the muscular wall of the vessel we call SC. It is analogous to muscle in vessel walls elsewhere because it is a primary determinate of the dimensions of not only the vessel lumen but also the tension present in the vessel wall. Studies substantiate that physiologic ciliary muscle tension is necessary for regulated control of aqueous outflow (Rohen et al., 1967).

Ciliary muscle receptor-systems can sense and coordinate responses to pressure changes (Tamm and Lütjen-Drecoll, 1997; Gabelt and Kaufman, 2005; Flügel-Koch et al., 2009). In the aqueous outflow pump model, the TM-ciliary muscle unit is inseparable because the muscle provides sensory and motor functions that control both the extent of TM distention into SC (Van Buskirk, 1976, 1982) and the elastance/stiffness necessary for pressure-dependent TM responses (Xin et al., 2016). Ciliary Muscle Motion Video (Vid 6) 1-s2.0-S1350946220300896-mmc6.mp4.

#### 3.2.1. Ciliary muscle connections, vector forces

The ciliary muscle has dual properties with the longitudinal ciliary portion having features of fast-acting type II striated muscle; the longitudinal muscle tips attached to the TM tendons stain particularly intensely for such markers. In contrast, the radial and circular regions have markers more characteristic of slow type-I fibers of smooth muscle; (Flügel et al., 1990a, 1990b). The longitudinal portion of the ciliary muscle connections divide about equally between the direct connections to the TM lamellae tendons and those that connect to the scleral spur (Hogan et al., 1971) (Fig. 10).

While the longitudinal portion of the muscle pulls the base of the TM closest to the sclera posteriorly, the radial and circular portion of the ciliary muscle pull the more interior connections of the TM inward. The resulting vector forces cause the scleral spur and the TM tendons to rotate both posteriorly and inward (Van Buskirk, 1982; Lütjen-Drecoll and Rohen, 1996). We can observe ciliary muscle tension in real-time. Placing tension on the ciliary muscle in fresh tissue causes striking elongation of the TM lamellae. With the release of tension, the lamellae’s elastance properties cause immediate recoil anteriorly toward Schwalbe’s line and outward toward SC external wall, thus reducing the SC lumen size (Johnstone, 2016) (Fig. 10) (Vid 6).

#### 3.2.2. Ciliary muscle tendons, elastic fiber system, synergistic tensional integration

Elastic-like properties of ciliary muscle tendons provide a synergistic mechanism that can work in concert with cellular mechanisms to ensure the tensional integration of the TM tissues. The muscle tendons extend to an elastic fiber system that appears to have two principal functions, maintenance of their connections to juxtacanalicular cells and SC endothelium and as a support structure in the anchorage, expansion, and control of distension of the SC inner wall (Hann and Fautsch, 2011). There are three types of ciliary muscle tendons. Type A tendons run into the scleral spur, Type B form broad bands that penetrate the cornea, while Type C are brush-like and extend to the elastic-like fiber system of the TM.

The type C tendons form a network of elastic-like fibers (cribriform plexus) that provide connections extending from the ciliary muscle tendons to the trabecular lamellae, juxtacanalicular cells, and SC inner wall endothelium. Ciliary muscle tone can thereby directly influence the fiber system of the cribriform plexus and its connections to SC inner wall endothelium (Rohen et al., 1981). The direct connection of the juxtacanalicular cells to the elastic tendon network suggests the cells experience elastic fiber system-related tension.

With elevated pressure, the elastin/elaunin struts in the JCT straighten. (Fuchshofer et al., 2006; Hann and Fautsch, 2011). The associations have led investigators to propose that glaucoma may, in part, be a result of degeneration or alteration of the elastin-like fibers. Glaucoma-related proteins of the elastic tendon network include fibrillin-1, fibrillin-2, versican, latent TGFβ-binding proteins, and microfibrillar-associated proteins. (Hann and Fautsch, 2011; Gonzalez et al., 2012; Kuchtey et al., 2013; Vranka et al., 2015; Filla et al., 2017; Papadopoulou et al., 2017).

Under collector channels, the JCT can expand by up to 200%; correspondingly, both elastin and FBN-1 microfibrils can stretch up to two times their original length (Hann and Fautsch, 2009). The elastic fiber system’s properties suggest that it functions along with the juxtacanalicular cells as a biomechanical unit that acts synergistically to maintain the juxtacanalicular region’s tensional integration. The elastic fiber network also serves as a mediator of integrin based signaling between TM lamellae, juxtacanalicular, and SC endothelial cells due to their role in IOP-dependent tensional integration.

Three types of plaques develop in the cribriform plexus of eyes with open-angle glaucoma. Elastic fibers can appear as “plaque-like deposits and later as sheath derived plaques. Two of the three plaque types can develop aberrant changes in the elastic-like fibers that can become enormously thickened, while the third type is a separate entity (Rohen et al., 1981). A significant increase in plaque-like material is present between ciliary muscle tips in glaucomatous compared with normal eyes (Lütjen-Drecoll et al., 1986).

On the other hand, six eyes with elevated IOP had no increase in deposits suggesting that plaque formation may not be the initiating event in the pathogenesis of elevated IOP in glaucoma (Lütjen-Drecoll and Eichhorn, 1988). The sheath-derived plaques may cause stiffening, reducing the elastic fibers’ ability to participate in distention and recoil of the TM tissues. The reduced motion may reduce responses to both mean and pulsatile pressures leading to aberrant sensory and motor behavior in glaucoma.

#### 3.2.3. The ciliary muscle controls tissue motion from the TM to distal pathways

Two parameters defined by ciliary muscle tension affect the geometry and biomechanics of all the outflow system structures. First, the ciliary muscle tension determines the boundaries of pressure-dependent distention of the TM into SC (Ellingsen and Grant, 1971b; Van Buskirk, 1976, 1982). Second, the stresses of ciliary muscle tension regulate the anterior-posterior length-tension relationships of the trabecular tendons and their attached TM lamellae (Johnstone, 2016) (Fig. 10). Within the trabecular tissues, the elastic fiber system also acts synergistically to maintain IOP-dependent relationships. IOP loading forces move the TM tissues outward toward SC, but the ciliary muscle contractile state provides a counterbalance. The continuous interplay of stresses unites the entire ciliary muscle-TM lamellae-elastic fiber complex into a tensionally integrated system (Kaufman and Barany, 1976; Hann and Fautsch, 2011).

Ciliary muscle tension also induces vector forces that pull the scleral spur and TM lamellae away from SC external wall, increasing the intertrabecular spaces. Tension on the tethered juxtacanalicular cell cytoplasmic processes and SC endothelium, as well as the elastic fiber system, enlarges the juxtacanalicular region, at the same time, pulling SC endothelium away from SC external wall (Johnstone and Grant, 1973a; Grierson and Lee, 1975a; Van Buskirk, 1982). The SC inlet valve entrances, an integral part of the SC inner wall, are placed under increased tension with increased ciliary muscle tension, causing them to elongate and stretch (Fig. 10) (Van Buskirk, 1982; Johnstone, 2016) (Vid6). The SC inlet valve tension pulls the leaflets of the SC outlet valves open, thus providing the ciliary muscle with a central role in relationships throughout the aqueous outflow system (Hariri et al., 2014).

#### 3.2.4. Ciliary muscle contractility – primary determinate of TM lamellae elastance/stiffness

The establishment of physiologic loading forces is essential before assessing the elastance/stiffness of tissues (Fung, 1996; Humphrey, 2002). Loading forces determine the stiffness of muscle-tendon complexes. Elastance/stiffness curves are not linear in muscle. Instead, muscle stretches relatively easily under low loading forces but rapidly becomes stiffer as loading forces increase (Levick, 2010).

The resting or steady-state length determines the steady-state loading tension or prestress in muscle and tissues with tendon-like properties such as the TM lamellae. Thus, the loading force that the ciliary muscle contractile state provides is a primary determinate of the TM lamellae’s elastance/stiffness. The combined ciliary muscle-TM lamellae stiffness is relevant to assessing the pressure-dependent TM motion that controls IOP. Elastic tissue anchors the ciliary muscle’s posterior attachment to Bruch’s membrane, forming a continuum that extends to the optic nerve. With age, elastic fibrillar material increases, likely causing decreased compliance (Tamm et al., 1991).

Pulsatile behavior is associated with pressure-volume loops of the relevant chambers, such as those of the heart and the lymphangions, which are the functional units of the muscular lymphatics (Quick et al., 2008, 2009). The larger the stretch in diastole, the larger the stroke work achieved in systole, the behavior known as the Frank-Starling mechanism. “The energy of contraction of … muscle fiber is proportional to its length at rest” (Patterson et al., 1914).

The contractile state of the ciliary muscle is a principal component of the ciliary muscle-TM lamellae complex responses to pressure and dimensions of the chamber we call SC. Therefore, the ciliary muscle contractile state is a primary determinate of the systolic and diastolic dimensions of SC. The contractile state of the ciliary muscle is a property consistent with the Frank-Starling mechanism present at the whole organ and cellular level in other muscle systems (Sequeira and Van Der Velden, 2015).

The efficiency of muscle contraction depends on the steady-state length of the muscle maintained within a narrow range. An IOP change causes an alteration in the load presented to the ciliary muscle, and as occurs in other muscles, sensing of a load change will result in concomitant changes in myogenic tone, an intrinsic feedback mechanism. With age, the position of the ciliary muscle relative to the TM lamellae shortens (Strenk et al., 2010, 2018), thereby altering the steady-state length, a factor essential to regulating muscle sensory and motor properties.

#### 3.2.5. Ciliary Muscle-TM tension, prestress, cyclic stress, preconditioning

The aqueous outflow pump model permits introducing the concept of a highly mobile TM that continually changes its bulk configuration in response to mean pressure, oscillatory pressures, and transients. Its tensionally integrated responses set up the conditions for prestress. Prestress is the introduction of internal stresses into tissue and cells achieved through tension or compression.

IOP provides a loading force on the TM, forcing it outward, favoring distention into SC. The ciliary muscle contractile force continuously counterbalances the TM distention, providing the prestress required for the ciliary muscle involvement in pump-conduit regulation of IOP. The opposing IOP and ciliary muscle contractility also provide the loading force that determines the TM-ciliary muscle unit’s elastance/stiffness. Optimized elastance is crucial to aqueous outflow pump function, and OCT provides a means of studying TM-ciliary muscle elastance (Xin et al., 2017) and the clinical surrogate, TM motion (Xin et al., 2018).

Preconditioning is the process of applying oscillatory loading cycles until stress-strain results become repeatable. The process provides stable ex vivo conditions in muscle preparations comparable to those present in vivo (Levick, 2010). Under physiologic conditions, continuous TM oscillations precondition the ciliary muscle bundles in vivo. Mean and oscillatory prestress, along with preconditioning, establish a loading and unloading environment remarkably different from that experienced ex vivo (Humphrey, 2002). To summarize, IOP and ciliary muscle contraction’s opposing forces provide stable prestress while oscillations and transients ensure preconditioning, primary determinates of elastance/stiffness of tissues in vivo. Elastance/stiffness of the ciliary muscle-TM lamellae complex is the primary property of interest in assessing the aqueous outflow pump’s pressure-dependent functional behavior.

### 3.3. SC inner wall endothelium – a deforming, load bearing structure

#### 3.3.1. Schlemm’s canal inner wall endothelium

SC walls are part of an endothelial-lined vascular continuum that extends to the veins and the heart. Endothelial cells lining SC have a different origin than juxtacanalicular cells or endothelial cells lining the TM lamellae (Tamura et al., 1991; Coupland et al., 1993; Hamanaka et al., 1997; Hamanaka et al., 1997, 1997). SC endothelial cells have properties of vascular endothelium (Hamanaka et al., 1992, 1997) as shown by the presence of Von Willebrand factor (factor VIII-related antigen) (Krohn, 1999) and cellular inclusions (Freddo et al., 1980), including Weibel-Palade bodies. The SC cells also have features of a modified lymphatic channel (Aspelund et al., 2014; Kizhatil et al., 2014; Park et al., 2014). SC endothelial cell cytoplasmic processes connect through robust desmosomes (Grierson and Lee, 1974b), a feature of lymphatics, and arachnoid villi, but not vascular endothelium (Desai et al., 2009). Moreover, a lymphatic defect causes ocular hypertension and glaucoma in mice (Thomson et al., 2014). Differences in properties from TM lamellae cells and similarities to lymphatics emphasize the unique features and the specialized role of the SC cells and their lymphatic-like properties.

#### 3.3.2. Cytoskeletal elements of SC endothelium — motor and sensory functions

Components of the trabecular endothelial cell cytoskeleton include microfilaments (F-actin) (Ringvold, 1978; De Kater et al., 1992) and intermediate filaments (vimentin) (Weinreb and Ryder, 1990; Tomarev et al., 2003). Actin provides active tissue motion and tension, while intermediate filaments composed of vimentin undergo marked deformation without disruption (Alberts, 2002).

The cytoskeletal elements determine constraints placed on pressure-induced SC cell deformation, thus defining the cell’s ability to store elastic energy necessary for the maintenance of a homeostatic wall stress setpoint (§5). The cytoskeleton also serves a crucial sensory role by maintaining optimized cell surface topology, which determines the ability to sense and respond to shear stress (Rodbard, 1975). Shear stress modulates cytoskeletal behavior and induces multimerization of cochlin found in glaucoma patients, which may play a role in IOP control (Picciani et al., 2007).

#### 3.3.3. “Giant vacuole” misnomer - pressure-dependent cell deformation, not vacuoles

Reports from the 1950s describe “giant vacuoles” in the distending cytoplasm of SC endothelium (Garron et al., 1958; Holmberg, 1959). Vacuoles are “membrane-bound fluid-filled cavities within the cytoplasm of a cell.” (Phillips, 2004). The term suggests a dynamic metabolic process. The cause of the “giant vacuoles eluded investigators for many years. By 1971, giant vacuoles had been the subject of numerous studies, but the mechanism of their formation was unknown (Hogan et al., 1971).

Fifteen years after the initial description of “giant vacuoles,” a report demonstrated the ability to systematically and reproducibly cause or eliminate the vacuoles by changing pressure gradients across SC endothelium (Johnstone and Grant, 1973a). Once recognized, confirmatory reports quickly followed (Lee and Grierson, 1974). The initial report describing their pressure-dependent formation concluded that the “giant vacuoles” rather than being intracellular, metabolically-induced structures, were instead distending cells with both the cytoplasm and nucleus deforming in response to pressure gradient changes (Johnstone and Grant, 1973a; Johnstone, 1979).

Rather than forming intracytoplasmic vacuoles, individual cells of the SC endothelial sheet develop progressively larger hemispherical outpouchings of not only the cytoplasm but also the nucleus (Johnstone and Grant, 1973a; Grierson and Lee, 1974a, 1974b, 1975a, 1975b; Johnstone, 1974) (Fig. 9). Histologic sections selected to show only cytoplasmic distension will lead to the erroneous impression that vacuoles are developing in the cell cytoplasm rather than the complete picture of cytoplasmic and nuclear deformation. The size and number of pseudovacuoles in the cytoplasm and nucleus increase in response to a progressive IOP elevation, and the pressure effect is reversible (Fig. 8) (Johnstone and Grant, 1973a).

The ability to induce and eliminate the pressure-dependent formation of the pseudovacuoles persists in enucleated eyes long after death (Johnstone and Grant, 1973a). Hypothermia or metabolic poisons do not inhibit the ability to induce pressure-dependent cell deformation that induces the pseudovacuoles; such findings are consistent with rearrangement of the cell configuration by mechanical instead of active metabolic mechanisms (Van Buskirk and Grant, 1974). Since “giant vacuoles” are a passive cellular response resulting from reversible pressure-dependent deformation, the term detracts from the entire cell’s physiologic properties that result in distension and recoil of both the cytoplasm and nucleus. Although the pseudovacuoles form passively in response to pressure, metabolic activity to control the ciliary muscle’s functional behavior and appropriate elastance of the TM lamellae, juxtacanalicular, and SC endothelial cells is necessary for the pump to function appropriately in vivo.

#### 3.3.4. Transendothelial pores

Transcellular pores are a topic of continuing intense study to identify the abnormality in open-angle glaucoma. Recent reports review the evidence supporting the role of pores in outflow regulation (Stamer et al., 2015; Vahabikashi et al., 2019). Investigators first observed opening in SC endothelium using TEM that involved standard fixation and dehydration techniques (Garron et al., 1958). The SC endothelium openings were interpreted as pores, a finding that finally appeared to solve a 19th-century puzzle; how does aqueous enter SC from the juxtacanalicular space?

SC endothelial cells have tight junctions (zonule occludentes) and desmosomes that join cells to one another and form a continuous belt-like region of contact that encircles each cell’s edges. Tight junctions, unlike those of sinusoidal and fenestrated endothelium, prevent active transendothelial fluid transfer across their walls (Hogan et al., 1971; Levick, 2010). Knowledge of the SC endothelium’s surface area, and the total fluid volume crossing that area in a defined time, permits calculating its hydraulic conductivity. If aqueous passed across SC endothelium, it would require hydraulic conductivity one-hundred times greater than any known non-fenestrated endothelium (Johnson et al., 2002).

Bill and Svedberg found a pore density of 1840 pores/mm, concluding that pores could account for only 10% of aqueous outflow resistance (Bill and Svedbergh, 1972). The low SC endothelial lining resistance of such studies does not provide an IOP loading mechanism for the SC endothelial sheet and tethered TM lamellae to distend or the SC endothelial cells to deform. SC endothelium deformation and elastance that maintains the TM relationships to SC external wall are not achievable, nor is the TM tissue’s ability to sense and regulate volume and pressure. An interposed ECM filter preventing SC endothelial cells’ exposure to resistance would further prevent SC endothelium from experiencing the resistance necessary for deformation.

Preparation artifacts may explain the initial reports and other later fixation-based reports using TEM, SEM, and block-face SEM. SC inner wall endothelial pores occur artifactually due to fixation (Sit et al., 1997), and the global view provided by SEM imaging makes the widespread presence of artifactual pores in fixed tissue evident (Lee and Grierson, 1975). SEM, TEM, and blockface SEM techniques all use the tissue fixation and dehydration techniques that induce reproducible, titratable, artifactual pores (Johnson et al., 2002). Large round openings with the absence of the entire anterior surface of the distending cell intersperse with markedly angular, slightly angular, or small structures with a uniformly round appearance in the same specimen (Bill, 1970; Bill and Svedbergh, 1972; Lee and Grierson, 1975). Limiting identification to round structures does not resolve the issue because round transcellular pores are also known to be a reproducible artifact of preparation (Lee and Grierson, 1975).

Marked initial tissue swelling, followed by shrinkage, results from the required alcohol dehydration protocols used to prepare fixed tissue. The dehydration can reduce the final tissue volumes to as little as 70% (Bloom and Friberg, 1956). Variation in divalent ions used in solutions prior to the dehydration steps affects the amount of swelling and shrinkage, another confounding variable. Cell membranes are composed primarily of lipids; even with the use of heavy metals to stabilize membranes, lipid losses can be substantial (Morgan and Huber, 1967; Saunders et al., 1967).

In an investigation by noted authorities, their calculations indicate that fixation causes pores, fixation time can titrate pore frequency, and pores may be absent without fixation. “If true, this implies that all (or nearly all) inner wall pores observed by SEM are fixation artifacts” (Johnson et al., 2002). Similarly, “Ideas about inner wall pores could be qualitatively and quantitatively wrong. If what are seen as pores are in whole or in part a fixation artifact, then a fundamental rethinking of the inner wall’s physiology is needed. In particular, the inner wall’s influence on the outflow facility becomes a wide-open question” (Ethier, 2002). The evidence and their conclusions have stood the test of time, neither refuted nor even challenged.

Supporting the above conclusions, a freeze-fracture study that avoids the fixation and dehydration steps found the SC endothelium to be a continuous lining with no evidence of transendothelial pores (Raviola and Raviola, 1981). Laboratory studies can induce transendothelial pores, but the above studies suggest their presence in living humans remains an open question.

### 3.4. Schlemm’s canal – a compressible chamber

#### 3.4.1. SC dimension changes –the concept of a compressible chamber

SC is a continuous torus circumscribing the limbus with boundaries defined by the sclera, the TM, and the scleral spur. The torus has a lumen with a flattened elliptical cross-section, 190–370 μm in length in the anterior-posterior plane (Hoffmann and Dumitrescu, 1971) while the total circumference is ~36 mm (Hogan et al., 1971). The shape of SC varies; the height of SC lumen typically measures ~50 μm at its posterior base but narrows to about 5–10 μm at its apex in ex vivo eyes.

Intraocular pressure, TM lamellae elastance, and ciliary muscle tension are primary determinates of SC dimensions in vivo. The SC lumen area can be absent at high pressures or very sizable at low pressures. (Fig. 8). The size of SC changes with ciliary muscle tension (Fig. 10) and with changes in pressure, as is demonstrated by systematically controlling IOP during both in vivo and ex vivo fixation (Fig. 8) (Johnstone and Grant, 1973a; Van Buskirk, 1982).

#### 3.4.2. Direct observation of SC dimension changes

Real-time videomicroscopy in unfixed ex vivo eyes, as discussed in S3.1.3 (Vid4), demonstrates TM motion (Johnstone et al., 2014a). This section explores the findings from the perspective of SC lumen dimension changes allowing it to act as a compressible chamber. Pulse waves caused SC lumen closure within 586 ± 219 ms. SC height decreased from 85 to 16 μm, and the length of the region of SC closure increased from 0 to 166 μm. Spontaneous TM tissue recoil causing restoration of initial SC dimensions occurs within 526 ± 132 ms. The time frame demonstrates that the TM tissues can distend and then spontaneously recoil, causing large SC lumen dimensions within the normal cardiac cycle. Rapid SC dimension changes demonstrable in fresh tissue illustrate the usefulness of direct observation as a prelude to the development of theories related to tissue mechanics.

### 3.5. Schlemm’s canal inlet valves – structures crucial to outflow regulation

We propose that all aqueous outflow enters SC by passage through the SC inlet valves because of the multiplicity of anatomic and functional properties that we have demonstrated. In addition, tracer studies in living primates that maintain a physiologic TM configuration demonstrate the absence of pores (Johnstone, 2004). The discovery of SC inlet valves resulted from techniques developed in the 20th Century (§4.1.4). The SC inlet valves’ characterization requires a reassessment of Leber’s 19th-century conclusion that flow requires aqueous movement through a passive filter along the SC inner wall. The presence of open conduits able to carry aqueous into SC permits developing a very different framework, that of an aqueous outflow pump functioning like other vascular circulatory loops that return blood and lymph to the heart.

SC dilation is the technique that opens a previously hidden world of structures, structural relationships, and pressure-dependent behaviors unobtainable by traditional means. The 20th-century technique of in vivo reduction of IOP below EVP caused blood reflux into SC. After blood dilated SC, eyes were fixed while maintaining in vivo conditions. Techniques developed in the 21st Century employ either viscoelastic or the hydrostatic pressure of aqueous to dilate SC (§4.2).

**Fig. 14.**
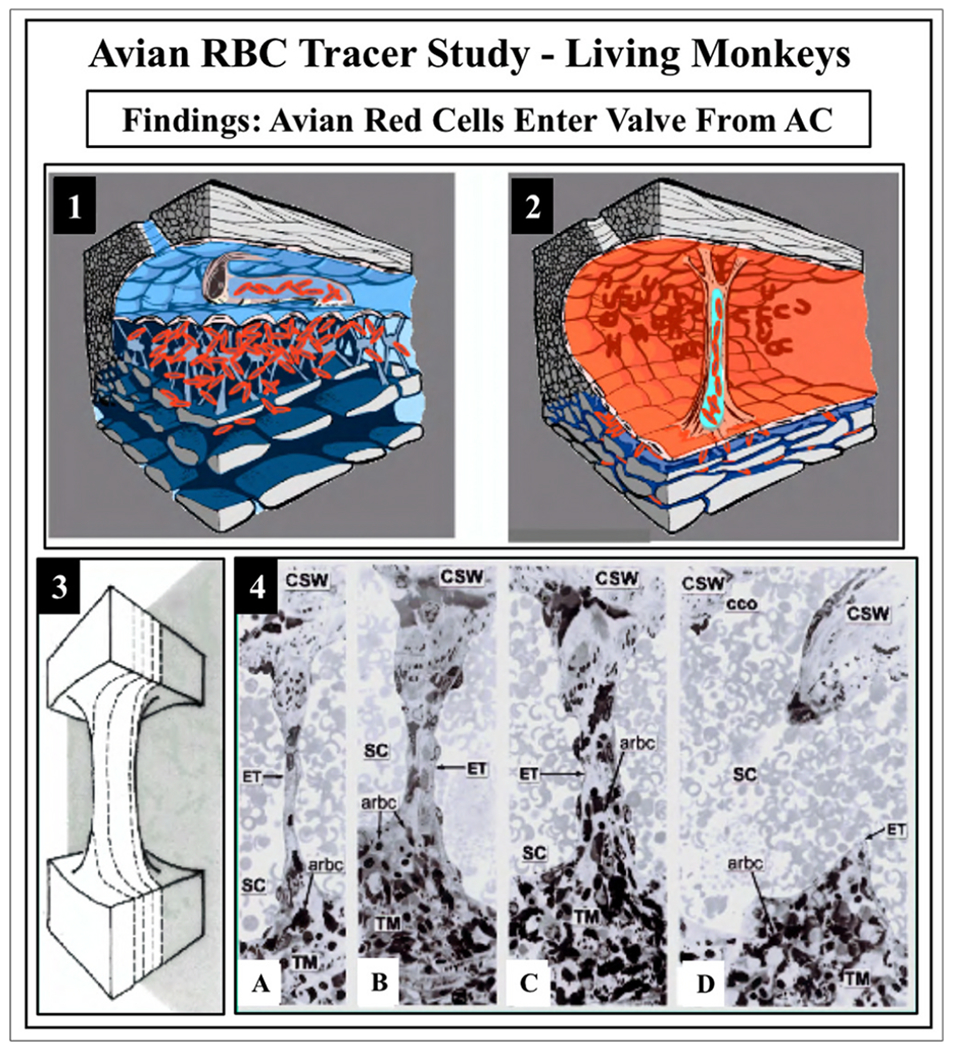
Red blood cell tracers fill the lumen of Schlemm’s canal inlet valves. Avian red blood cells (arbc) introduced into the anterior chamber (AC) as a tracer in living *Macaca mulatto,* monkey eye. **(1)** The arbc enter and fill a Schlemm’s canal (SC) inlet valve. **(2)** Gradual reduction of intraocular pressure (IOP) to 0 mm Hg in vivo causes SC to dilate and blood refluxes into the canal, because IOP is lower than episcleral venous pressure. Dilation of SC causes straightening and stretching of SC valves between SC walls enabling single radial sections to capture their full length. **(3)** Illustration of serial radial sections along a Schlemm’s canal inlet valve length. **(4)** From left to right: **(A–D)** are representative serial histologic sections encompassing the entire width of a Schlemm’s canal inlet valve depicted in **(3)**. The endothelial lining (ET) of the SC valve is continuous with SC inner wall endothelium. The SC inlet valve spans across SC to attach to the corneoscleral wall (CSW). Avian red blood cells are present in the trabecular meshwork, and the juxtacanalicular space. In a central section through a Schlemm’s canal inlet valve, red cells fill the length of the lumen, as shown in C. In 4B and 4C, note the two collagenous supporting structures at the SC valve distal end with a narrow space where they meet. From Johnstone M, The aqueous outflow system as a mechanical pump, J Glaucoma 13, 421–438, 2004.

Figs. 2,11 and 12 use different imaging modalities to demonstrate SC inlet valve appearance, relationships, and openings at their distal end that communicate with SC lumen. Figs. 8 and 13 illustrate the changing configuration as pressure increases, and the TM comes into apposition to SC external wall. Fig. 14 illustrates the findings with red cell tracer studies (Fig. 14). The SC inlet valves occur with a frequency of about two per millimeter around the circumference of SC (Smit and Johnstone, 2002) (Jamil et al., 2010; Martin et al., 2013)abs.

#### 3.5.1. Multiple modalities document SC inlet valve structure and functional behavior

This section focuses on the evidence for the presence and function of aqueous inlet valves. First, we examine clinical evidence, then correlate the evidence with seemingly unrelated laboratory studies by recognizing that we are examining the same structures by different means.

Clinical studies include the unroofing of SC in the operating room, demonstrating the SC inlet valves’ remarkable elasticity. Aqueous gushes from the SC inlet valves’ lumen when they are stretched beyond their breaking point, illustrating their role as a conduit carrying aqueous (Johnstone, 2004) (Vid 2). Operating room gonioscopy provides direct observation of aqueous flow into SC through the aqueous inlet valves synchrony with the ocular pulse (Johnstone, 2004) (Vid 2). Fig. 5 illustrates the propagating wave of aqueous as it passes through oscillating pathways constrained to a path like that seen in the multiple imaging modalities that characterize the aqueous valves.

Laboratory studies using diverse techniques provide evidence of the aqueous inlet valves that we can correlate with the clinical findings noted above. The structural features, response to ciliary muscle tension, pulse induced motion, and tension on SC outlet valves at CC entrances is apparent from dissecting microscope techniques (Johnstone et al., 2014a) illustrated in Fig. 10 and (Vid 6). Structural feature details become apparent using brightfield microscopy that demonstrates the funnel entrance from the TM, the conduit region, and attachment to the hinged flaps at SC external wall (Johnstone and Grant, 1973a; Van Buskirk, 1982) (Johnstone and Mills, 2004)abs. The same structural features present with brightfield microscopy receive confirmatory evidence from phase-contrast (Jamil et al., 2010; Curtiss et al., 2011)abs and differential interference microscopy studies (Jamil et al., 2010; Curtiss et al., 2011)abs.

Fine structure of the walls and features within the lumen of the SC inlet valves are made manifest through transmission electron microscopy studies (Johnstone, 2004). Geometric relationships of the SC inlet valve to the TM and SC external wall and the presence of a lumen are visible in SEM images in Figs. 11 and 12, and are available in additional references (Smit and Johnstone, 2000)abs (Smit and Johnstone, 2002; Johnstone, 2004, 2016; Hariri et al., 2014; Xin et al., 2017). Tissue composition and 3D relationships become apparent with confocal fluorescence microscopy and immunohistochemistry studies (Fig. 11) (Jamil et al., 2010; Johnstone et al., 2011d; Martin et al., 2013)abs. Structure, geometric relationships, and motion are evident from OCT imaging (Hariri et al., 2014; Johnstone, 2016; Xin et al., 2016, 2017).

Aqueous passage through the SC inlet valves is the subject of tracer studies in living primates; if larger tracers such as red cells can pass through the SC inlet valve lumen, we can reasonably conclude that aqueous can do so as well. Direct communication between the AC, the juxtacanalicular space, and the SC inlet valves’ lumen is apparent. Studies demonstrate that avian red blood cells (RBC) introduced into the AC enter the funnel-shaped region of the SC inlet valves and pass to the distal end (Fig. 14) (Johnstone, 2004). Direct communication of the distal end of the SC inlet valve lumen and the lumen of SC is apparent from in vivo fixation at positive pressure followed by in vivo reflux of blood. Primate red cell reflux from SC into the distal end of the SC valve lumen, demonstrating the distal communication with SC (Johnstone, 2004). Immunohistochemistry studies demonstrate that the SC inlet valves exhibit staining identical to that of the SC inner wall providing evidence of their constituent properties (Fig. 11).

Communication with the juxtacanalicular space and patency of the SC inlet lumen are exhibited by fluorescent microsphere introduction into the AC with subsequent passage through the entire length of the lumen (Fig. 11) (Martin et al., 2013)abs. Together, the constellation of modalities documents the structure and function of the SC inlet valves providing evidence that they are endothelial lined conduits permitting direct passage of aqueous from the TM into SC.

SC inlet valves are very frequently present in histologic sections, and reports in the literature document their regular presence and properties. However, the literature concerning pores in SC endothelium does not mention the presence of the inlet valves. An explanation may be the lack of precision in terminology. Some histology studies call all structural elements in SC a septum. When assigned a term such as a septum that suggests a structure with limited relevance to flow, investigators can easily overlook anatomic features with crucial functional importance.

A septum represents a partition between two chambers or compartments, like the septa between the chambers of the heart (O’Toole, 2013). An example in the outflow system is the presence of septa at SC external wall, where they create a partition between SC and a circumferential deep scleral plexus (CDSP). Outflow system septa consist of collagen bundles with a composition like the adjacent sclera of SC external wall. The composition is apparent in unfixed tissue where dense white collagen of the sclera and septa appear to have an identical composition (Fig. 10) (§4.2.2).

The SC inlet valves do not partition SC lumen into compartments and have no resemblance to the collagenous septa along SC external wall. However, the SC valves are oriented obliquely within the circumference of SC and can present with several different configurations depending on pressure gradients, preparation technique, orientation, and the plane of sectioning (Figs. 8, 10–13, 13, 14). Careful observation, knowledge of the marked differences in location, geometry, constituent properties, and use of serial sections should reduce the likelihood of inadvertently overlooking their presence or confusing the SC valves with septa.

#### 3.5.2. SC inlet valve motion and dynamic links to outlet valves - microscopy

To assess SC inlet and outlet valve relationships in fresh, unfixed tissue, we developed a videomicroscopy system that permits direct observation of the pulse-dependent motion of the SC inlet and outlet valves (Johnstone et al., 2011d)abs (Vid 5) (§4.2.2). The SC inlet valves are diaphanous and semitransparent under normal light conditions, making them difficult to see without careful observation. Visualization is aided by (1) placing a black or blue background behind the tissue, (2) oblique illumination. Gently pulling on the ciliary muscle in these radial limbal segments causes SC to open, enabling easy visualization of the aqueous valves and their connections using a dissecting microscope (Fig. 10).

An intermittent stream of balanced salt solution directed perpendicular to the TM causes it to distend into SC and then recoil (Johnstone et al., 2014a)abs (Vid 4). Gentle suction on the TM pulls it away from SC external wall causing SC valves to elongate as they stretch between the walls of the canal (Vid 4). The release of suction causes immediate recoil of the SC valves and the TM.

SC regularly dilates when a cannula positioned over the sclera gently ejects a bolus of BSS toward the SC inner wall. The dilation reveals connections of SC inlet valves to the hinged flaps of the SC outlet valves. The attached SC inlet valves elongate and pull the white collagenous SC outlet valves open. Immediately following the bolus, the TM recoils, eliminating tension on the outlet valves, which then close. The inlet valves are cylindrical, semitransparent, and span between the walls of SC. In contrast, the outlet valves are white, like the collagen of the sclera. (Vid 4).

#### 3.5.3. SC inlet valves – appearance, connections, real-time motion using OCT

**Fig. 15.**
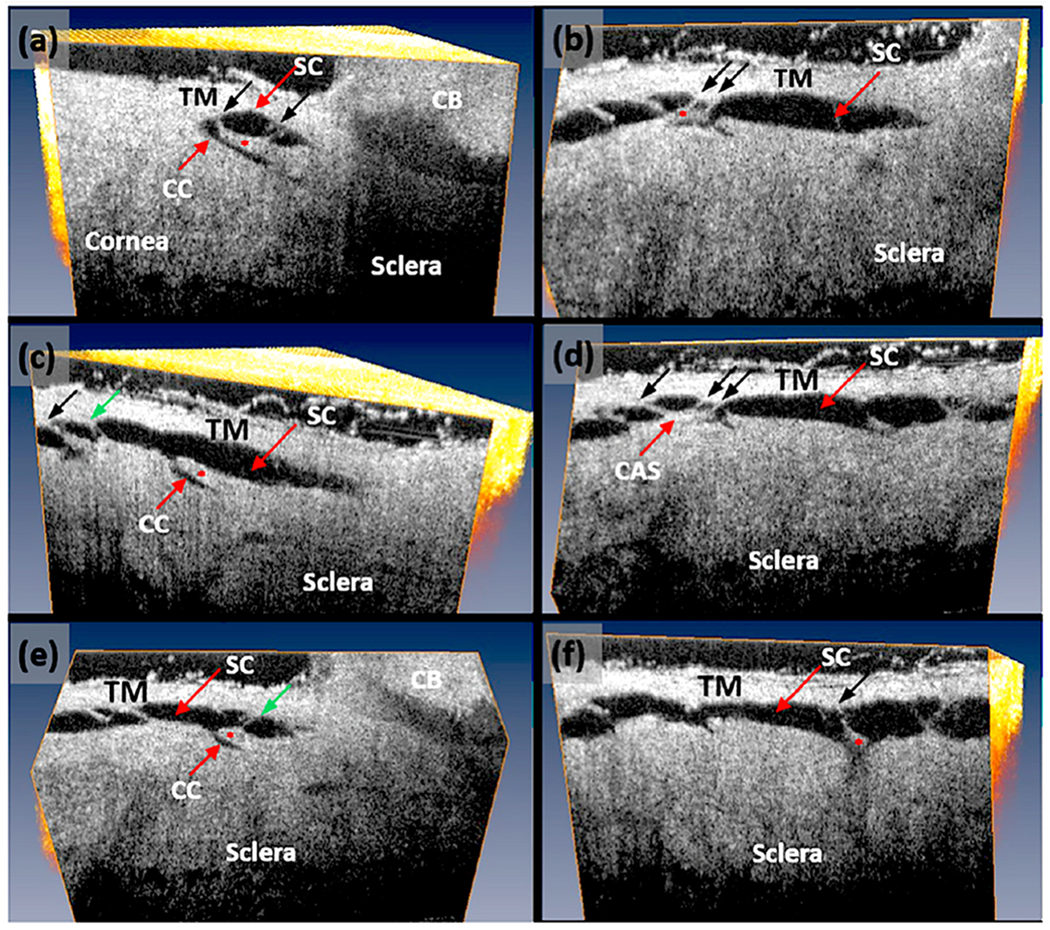
Schlemm’scanal outlet valve hinged flaps and trabecular meshwork connections. **(a–f)** A cannula inserted into the Schlemm’s canal (SC) lumen attaches at its other end to a reservoir controlling hydrostatic pressure within the lumen. A laboratory-developed high-resolution SD-OCT system permits a detailed examination of the TM, SC, and collector channels (CC). Images result from orienting the tissue volume to optimally view the hinged collagen flaps (red asterisks) at the CC entrances. A reservoir maintains a height providing SC lumen with a steady-state pressure of 50 mm Hg. The hinged collagen flaps provide mobile valved leaflets at the collector channels entrances. The hinged flaps attach to the TM by the SC inlet valve appearing as thin cylindrical attachment structures (CAS) (black arrows) spanning SC. Note that despite SC dilation, the oblique orientation of the SC inlet valves persists (black arrows). Some sections through the CAS reveal the Schlemm’s canal inlet valve lumen (green arrows) consistent with the dual role as conduits and connections between the TM and the SC outlet valves. Tissue source: Primate, *Macaca nemestrina.* From Xin C, Mechanical Properties of the TM and Collector Channels, PLoS One 11, e0162048, 2016.

**Fig. 16.**
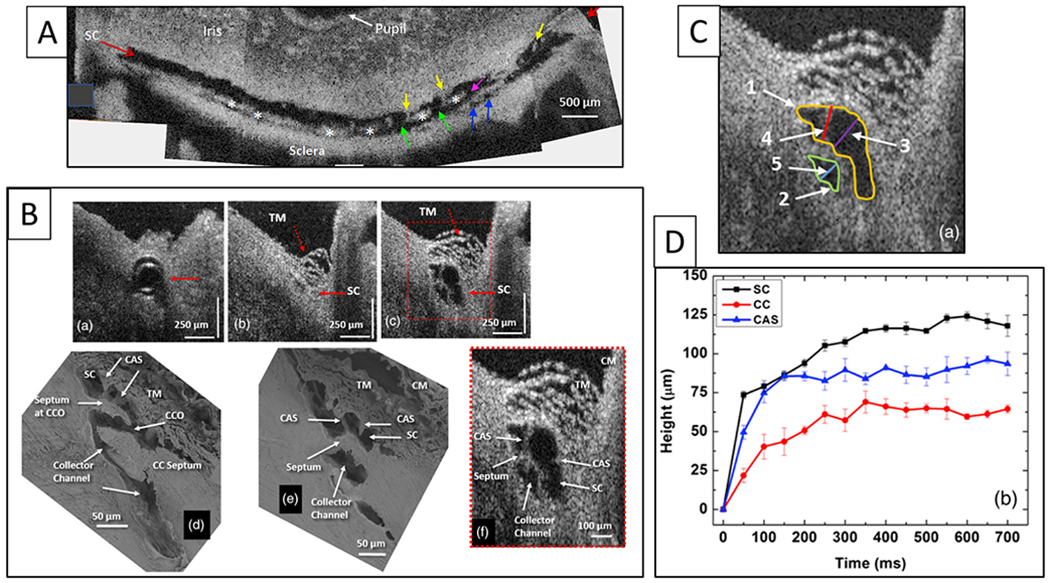
Synchronous lumen dimension changes of Schlemm’s canal and collector channels. **(A)** Representative 3-D reconstruction of the entire length of a limbal Schlemm’s canal (SC) region obtained from stitching five tangential sections together. The image plane is perpendicular to the canal circumference. The SC inlet valves appear as structures spanning between the walls of SC (yellow arrows). Purple and green arrows show collector channel ostia (CCO). Blue arrows show entrances of circumferentially oriented deep scleral plexus. Asterisks mark septa that divide SC from the circumferentially oriented deep scleral plexus (CDSP) parallel to SC. The tilted section shows different levels in the height of SC, thus exhibiting differing relationships. **(B)** Representative two-dimensional (2-D) structural OCT and scanning electron microscopy (SEM) images from the limbal region of an eye. **(a)** OCT image with the cannula inside SC (arrow). **(b)** At a location, ~150 μm away from the cannula tip before infusion of perfusate to raise pressure. Arrows identify SC as a potential space with no lumen, **(c)** shows the maximally dilated appearance at the same location resulting from a bolus of aqueous. Images **(d)** and **(e)** are representative SEM images from the limbal region. The SEM and OCT images mirror each other in illustrating the structural features of the outflow system. Original SEM images: 337× magnification. The image in **(d)** shows a collector channel entrance or ostia (CCO). A septum present at the CCO is attached to the TM by a Schlemm’s canal inlet valve, which is labeled here as a cylindrical crossing structure (CAS). Image **(e)** is the adjacent section from the same segment showing the transition from the region of a CCO in **(d)** to the circumferentially oriented deep scleral plexus (CDSP). The CDSP is labeled as a collector channel in **(d)**, **(e)**, **(f)** because publication of these figures was before recognition of CDSP as unique entities. The image in (f) is a 2× enlargement of **(c)**. CM, ciliary muscle. **(C)** Enlarged view of **(f)** in image **(B)** identifying the location of measurements in D. **(D)** Progressive increase in the height of SC, CC, and CAS with time during SC filling with a bolus of aqueous from a reservoir. SC (black curve), CC (red curve), Schlemm’s canal inlet valves (here labeled as CAS) (blue curve). Tissue source: Primates, *Macaca nemestrina.* From Hariri S, Pressure dependent TM motion with high-resolution OCT, J Biomed Opt 19,106013 1–10601311, 2014. Video - Linked TM and CC Motion 1-s2.0-S1350946220300896-mmc5.mp4.

OCT imaging technologies are discussed in §4.2.6. The imaging permits examination of experimentally controlled pressure-dependent steady-state configurations. In the same tissue, the technology permits examination of 3D volumes from any perspective Fig. 15 (Fig. 15) and real-time motion characterization Fig. 16 (Fig. 16). The combination of the ability to control and change SC dimensions in real-time while simultaneously capturing the motion with OCT provides insights into morphology and physiology previously unattainable (Hariri et al., 2014; Xin et al., 2016).

A meridional section in Fig. 16 shows cylindrical attachments between the walls of SC that represent the SC inlet valves. The inlet valves arise from the TM and course across SC to the hinged flaps or leaflets of SC outlet valves. A lumen is visible in the valves, providing a conduit for flow. High-resolution OCT illustrates the ability to watch the aqueous valves’ motion in real-time and quantitate configuration changes. As SC pressure increases, the SC inlet valves undergo marked elongation, thinning, and cause tension on the SC outlet valve entrances pulling them open. Vid 5 1-s2.0-S1350946220300896-mmc5.mp4.

#### 3.5.4. SC inlet valve functional implications

The SC valves provide conduits for flow into SC and prevent the backflow of blood. The large lumen of the SC valves provides a direct conduit connecting the juxtacanalicular space with SC and CC entrances. Since microspheres and even RBCs pass into the SC inlet valve lumen from the AC (Fi 14), aqueous can flow through them as well. The large lumen of the SC inlet valves easily explains the high hydraulic conductivity across SC endothelium that is 100X greater than expected if there was no direct conduit to SC. The SC inlet valves also have properties that cause them to collapse when pressure gradients reverse, allowing them to satisfactorily maintain the blood-aqueous barrier (Figs. 13 and 14) (Johnstone, 2004).

Contractile properties of SC inlet valves permit flow control. Brightfield, interference contrast, differential interference contrast, confocal fluorescence, scanning, and TEM studies demonstrate that the SC valves’ walls are continuous with and have properties like those of SC inner wall endothelium (Figs. 11 and 12) (Johnstone, 1974, 2004). SC inner wall endothelial cells undergo a rather remarkable elongation but then can recoil or contract (Johnstone and Grant, 1973a; Johnstone, 1974, 1979,1984, 2004; Grierson and Lee, 1975a), as discussed in detail in §3.3 and 5.2.4. Shortening of the cytoplasm and rounding of the nuclei that develop deep notches and folds is present both in SC endothelium and in the walls of SC valves in response to pressure reversal (Johnstone and Grant, 1973a; Johnstone, 1974). The behavior is like that of the endothelial walls of other vessels known to contract in response to pharmacologic agents (Majno et al., 1969).

Marked increases in length of the SC inlet valves are documented in vivo in humans (Vid 2), ex vivo with the operating room microscope, at the dissecting microscope (Johnstone et al., 2014a) (Johnstone, 2016) abs (Vid 5), and by OCT (Hariri et al., 2014) (Zhou et al., 2015)abs. The SC inlet valves recoil from their elongated configuration when pressure falls. The valve walls are composed of endothelium like that of SC inner wall. The recoil provides evidence of the ability of the valve endothelial walls to store and release energy in response to changing pressure gradients in SC. The inlet valves also change diameter in response to pressure gradient changes (Johnstone and Grant, 1973b; Johnstone, 2004; Zhou et al., 2015). SC inlet valve lumen dimensions are determined by intrinsic contractile properties, providing them with a crucial role in IOP control.

Pressure differences between the valve lumen and the surrounding SC pressure provide the SC valves with a Starling resistor’s properties. Starling resistors are collapsible tubes that enter and exit a surrounding reservoir that experiences changing pressure gradients (Knowlton and Starling, 1912; Fung, 1996). The downstream pressure beyond the reservoir determines the pressure drop. The tube’s distal end collapses if downstream pressure is lower than the pressure in the reservoir, so fluid does not flow. Physiologic systems widely employ the Starling resistor design principle; for example, the mechanism regulates the flow rate of fluid in the venous and lymphatic circulatory loops above the heart (Shapiro, 1977a, 1977b).

It is attractive to place control of aqueous outflow in the realm of design principles used to regulate flow and pressure in other fluid compartments in the eye (Morgan et al., 2016). The aqueous inlet valves have classic features of a Starling resistor system. They are attached to SC external wall, have collapsible walls, and are within a compartment with continuously changing pressures. Between the AC and the episcleral veins, a distance of one mm, the system maintains an eight mm pressure drop, a design challenge ideally suited to a Starling resistor mechanism.

#### 3.5.5. SC inlet valves provide a TM link to leaflets controlling CC dimensions

SC inlet valves provide a link between the baroreceptor-like TM and hinged flaps or leaflets of the SC outlet valves. The relationships are apparent with the dissecting microscope (Fig. 10) (Johnstone, 2016)abs, brightfield microscopy (Fig. 14) (Johnstone, 1974, 2004; Van Buskirk, 1982) TEM (Johnstone, 2004) and SEM (Smit and Johnstone, 2002; Hariri et al., 2014) (Martin et al., 2013)abs and OCT (Hariri et al., 2014) (Zhou et al., 2015; Johnstone et al., 2018)abs. The attachments ensure that when the TM moves, the SC outlet valve leaflets move. Microscope evaluation during BSS infusion or vacuum causes the meshwork to move away from the SC external wall. The separation of SC walls causes the SC inlet valves to stretch and then recoil when pressure is released (Vid2 and 5). Ciliary muscle tension results in similar evidence of elongation of the SC inlet valves, and resulting tension along their length is indicated by the deformation of a septum wall, causing enlargement of a CDSP lumen (Fig. 10) (Johnstone, 2016).

OCT studies (Fig. 16) demonstrate the ability to quantitate synchronous relationships between the SC and CC lumen dimensions and changes in the length of SC inlet valves (Hariri et al., 2014). The inlet valve length changes are concurrent with changes in SC and CC dimensions. OCT videos document the pressure-dependent elongation in real-time (Xin et al., 2016) (Vid 5).

### 3.6. Schlemm’s canal outlet valves – linkage to TM – role in IOP control

#### 3.6.1. SC outlet valves at CC: histology, SEM, immunohistochemistry

Rohen’s research describes SC outlet valves as short oblique septa forming lip-like thickenings of the SC external wall (Rohen et al., 1981). His perceptive observations note that the CC entrance structures’ organization should cause them to close because the pressure in the SC lumen will be higher than in the more distal channels. The study identifies structures arising from the TM that attach to the septa at CC; Rohen proposes that TM tension on the structures spanning between the TM and oblique septa at CC entrances is necessary to hold the entrances open.

**Fig. 17.**
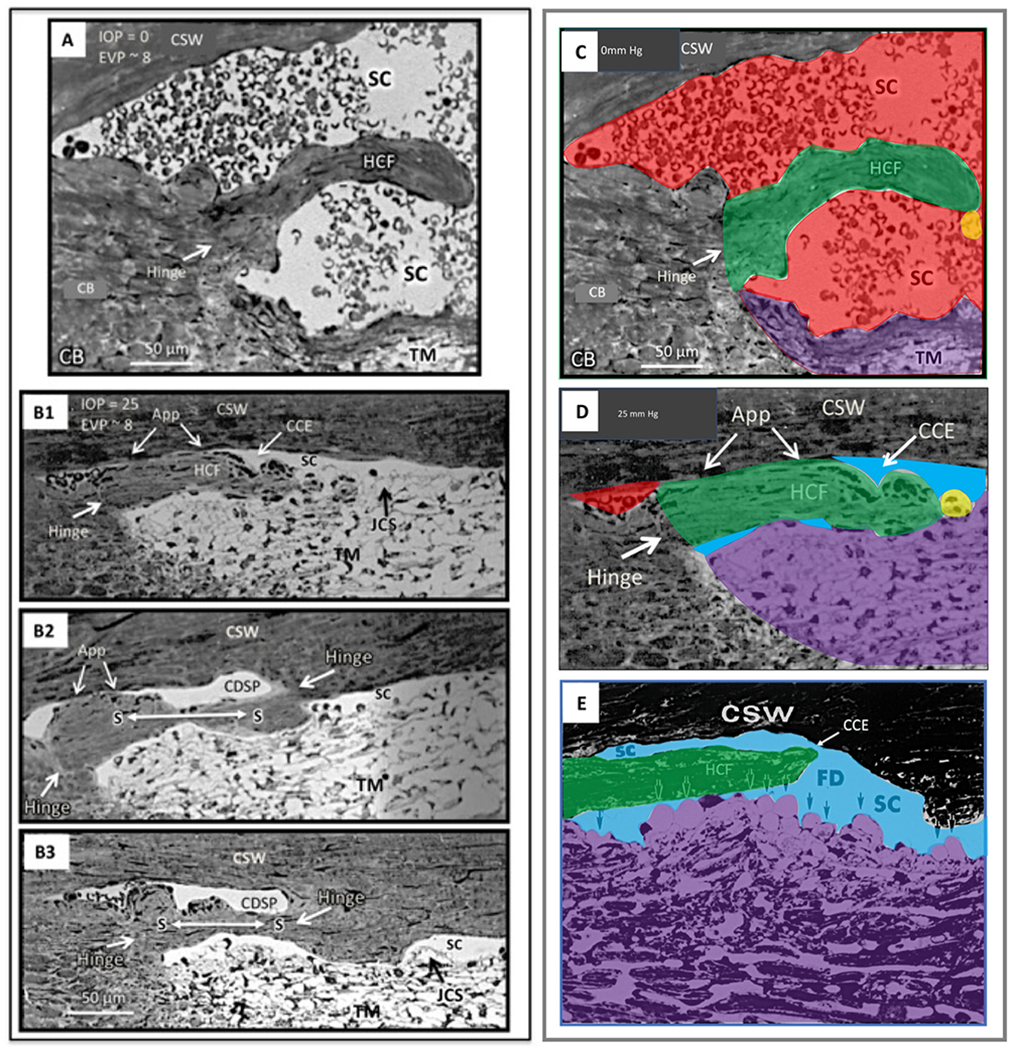
Schlemm’s canal outlet valves: Pressure-dependent appearance. In vivo fixation, while maintaining an IOP in **(A)** of 0 and **(B1–B3)** of 25 mm Hg. Images in serial sections of **(B1–B3)** illustrate the transition from a collector channel entrance to a circumferentially oriented deep scleral plexus (CDSP). Hinged collagen flaps (HCF) at collector entrances (CCE) are free at one end. The HFC can move freely in response to TM movement induced by pressure changes, thus permitting them to have a pressure-dependent valve-like function at CCE. In **(B2 and B3)**, a long septum (S) separates SC from a circumferentially oriented deep scleral plexus (CDSP). In (A and C), a HCF is far from the corneoscleral wall (CSW) of SC. The next serial section, not shown but depicted in C, contains a transcanalicular attachment extending from the TM to the tip of the HCF. In image **(B1)**, the hinged collagen flap at the entrance to a CCE is in apposition (App) to the CSW. Collagen fibers at the base of the hinge in **(B1–B3)** are circular running orthogonal to the plane of section, but are parallel in the hinged septum, providing a pivot point for motion. Septa dividing SC from CDSP are collagenous structures and differ from the aqueous valves that are transcanalicular endothelial-lined conduits spanning SC. Features of images **(C and D)**, derived from **(A)** and **(B)**, are color-coded as follows: Red, blood, blue, aqueous; green, hinged collagen flap; yellow, cylindrical attachments connecting to TM. Note blood in **(B1)** and **(D)** distal to the HCF. The apposition of the HCF to SC external wall prevents blood from entering the canal. **(E)** Ex vivo fixation of human eye at IOP of 50 mm Hg with TM distending or herniating into SC at a CC entrance at a fusiform dilation (FD) of SC external wall. A long HCF is present at the CC entrance. Tissue source: Primate, *Macaca mulatto.* From Johnstone, Howe Laboratory of Ophthalmology, Harvard Medical School 1972. Video - CC Open and Close 1-s2.0-S1350946220300896-mmc7.mp4.

The initial study of pressure-dependent outflow system motion in living non-human primates (Johnstone and Grant, 1973a) identified the pressure-dependent motion of the TM lamellae; the related studies also identified the aqueous valves spanning across SC to attach to specialized collagen flaps at CC entrances (Johnstone, 1974) (Figs. 14 and 17). Pressure gradient changes caused the TM to move and resulted in substantial alterations in the collagen flap configuration at CC entrances. The hinged collagen flaps that act as valves at CC entrances attach to the sclera at only one end. The hinged arrangement permits the flap to open and close the CC entrances. Studies of 3D relationships provide an explanatory mechanism for the SC outlet valve leaflets’ ability to respond to pressure changes (Figs. 16 and 17).

Dissecting microscope manipulation of the outflow system tissues provides direct observational evidence of the presence and the high level of mobility of the SC outlet valve leaflets (Jamil et al., 2010)abs (Fig. 10). Ciliary muscle tension causes the TM to move inward and posteriorly. The walls of SC then separate, generating increased tension on connections between the TM and the SC outlet valves, causing the valves to open (Jamil et al., 2010; Curtiss et al., 2011)abs. Brightfield microscopy studies with serial sections demonstrate the appearance of the SC outlet valves (Fig. 17) (Johnstone et al., 2011b)abs (Van Buskirk, 1982; Bentley et al., 2016; Johnstone et al., 2016), as does confocal and fluorescence microscopy (Martin et al., 2012)abs (Fig. 11).

Separation of SC walls for an independent examination of either the inner or outer wall tears away the SC inlet valves and their connections to SC external wall. The separation prevents the interpretation of SC valve relationships that are otherwise apparent. Laboratory techniques that use viscoelastics to dilate SC avoid disruption of the walls and illustrate the SC outlet valve configuration, as well as their connections to SC inlet valves and the TM (Smit and Johnstone, 2000)abs; (Smit and Johnstone, 2002; Johnstone, 2004, 2016; Hariri et al., 2014).

A recent study, separating the walls of SC for SEM, was correlated with light microscopy and was able to report flap-like structures at CC entrances as well as septa and tube-like structures. The authors concluded that these structural entities in the distal pathways might play a regulatory role in aqueous humor dynamics (Bentley et al., 2016).

**Fig. 18.**
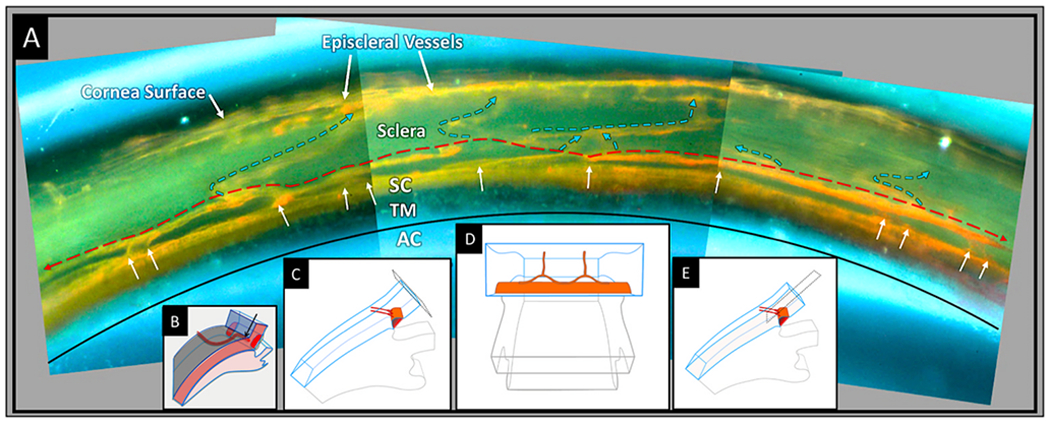
Circumferentially oriented deep scleral plexus visualization. A microvascular cast of the outflow system. **(A)**. The trabecular meshwork (TM) is between the black curved line and the orange Schlemm’s canal (SC) cast. White arrows indicate the location of collector channels (CC) arising from SC and connecting to a circumferentially oriented deep scleral plexus (CDSP), as is indicated by a thin dashed red line. The CDSP forms a relatively continuous communicating ring adjacent and parallel to SC. Intraseleral vessels exit the CDSP and pass through the sclera (blue arrows) to the surface of the eye where episcleral and aqueous veins are visible. Between SC and CDSP are long, thin layers of collagenous tissue that appear as septa in histologic and OCT sections. OCT and direct observation at the dissecting microscope reveal that the thin septa move in response to ciliary body tension and pressure gradient changes within SC. Septa movement causes the lumen of the CDSP to open and close, thus functioning as a pressure-dependent compressible chamber. **(B)** Schematic illustration from the microscope view through the cut corneal surface that reveals the uniform angle of exit of the CC from SC. A view of casts from the surface of the corneoscleral interface does not provide a view perpendicular to the sites where CC exit SC. The view looking along the axis of the exit sites inadvertently gives the impression that the collector channels course directly from SC to the episcleral veins. By looking through the cut corneal surface **(C)**, the view is perpendicular to the CC exit sites as in **(D)**, which is the orientation shown in the microvascular cast in **(A)**. The orthogonal view **(E)** makes the typical CC connections with the CDSP apparent. Tissue Source 78-year-old Caucasian male. From Johnstone Glaucoma Laboratory, University of Washington. Review Video in Fig. 16 – CDSP Open and Close 1-s2.0-S1350946220300896-mmc5.mp4.

Meridional sections through SC provide SEM images of the entire length of SC in the segments; they reveal round CC openings, septa, CDSP, and relationships that create a hinged flap where they join (Hariri et al., 2014; Carreon et al., 2017; Xin et al., 2017) (Martin et al., 2014, 2015)abs. The CC, septa, CDSP, and related hinged flap relationships are also evident when viewing microvascular casts (Fig. 18) (Carreon et al., 2017).

Removal of the TM permits looking directly at CC entrances with SEM, but the relationship of the CDSP that creates the hinged flaps is hidden from view; the septa overlying them in an axial plane preclude visualization. The SC inlet valves also have delicate attachments to the edges of the CC orifices that are easy to disrupt, leaving only “flap anchors,” thickened rims, or evidence of denuded epithelial connections (Bentley et al., 2016).

An SEM study, looking at SC external wall, has led to a description of multiple CC orifices with no hinged flaps and no SC inlet connection to the TM (Johnstone et al., 2014b). However, such techniques do not permit assessing whether hinged flaps are present, whether they open and close or whether they have connections to the TM. The problem with SEM studies that use en face imaging is that the septum edge adjacent to CC that constitutes the hinged collagen flap (HCF) moves in an axial plane relative to the en face images. Such SEM-based en face images cannot capture the HCF position changes that enable the structures to act as valves.

Studies with multiple modalities that avoid SC disruption lead us to the provisional premise that hinged flaps and SC inlet valves are regularly present at CC entrances when there is no disruption of SC walls. However, we cannot rule out some CC entrances not having the relationship, and further studies are necessary to clarify the issue. Another question, however, is whether all SC inlet valves lead to CC entrances. OCT studies of 3D volumes discussed below show that some of the SC inlet valves attach to septa overlying CDSP rather than to the SC outlet valves.

#### 3.6.2. SC outlet valves at CC– high-resolution OCT and 3D Micro-CT insights

High-resolution OCT is singularly valuable because it generates a 3D volume of the SC outlet valves. Surveying the volume permits finding an orientation that best captures the collagen flaps’ hinged nature and their relationships to CC entrances. The 3D volume also characterizes connections between the TM, SC inlet valves, and the SC outlet valves’ hinged flaps (Xin et al., 2017) (Hariri et al., 2014; Xin et al., 2016, 2017). Fig. 15 illustrates the hinged flap arrangement uniformly present at multiple CC entrances after optimizing the 3D volume orientation (Xin et al., 2016) (Vid 2). Real-time motion assessment demonstrates rapid configuration changes in response to a pulse wave, as seen in Fig. 16. Video OCT of CC Motion – (Vid 7) 1-s2.0-S1350946220300896-mmc7.mp4. Disruption of the SC inlet valve attachment to the outlet valve flap can cause a loss of motion of the SC outlet valve (Zhou et al., 2015) abs.

Studies with 3D micro-CT provide further evidence of hinged flaps at CC entrances that can change shape, allowing them to function as SC outlet valves. In immersion fixed eyes, a mean orifice size of 27.5 ± 5 μm was found, but eyes fixed at 10 mm Hg IOP had a mean orifice size of 40.5 ± 13 μm (Hann et al., 2011). Another 3D micro-CT study found a 3.7-fold increase in total occlusions of CC entrances in glaucoma compared to normal eyes. Visualization of CC entrances increased 24% in normal and 21% in glaucoma eyes at 20 mm Hg compared with 10 mm Hg. The SC volume, CC area, and diameter were decreased in glaucoma compared to normal eyes (Hann et al., 2014). The findings again demonstrate tissue geometry that permits CC entrance motion and different CC motion responses in glaucoma compared with normal eyes.

### 3.7. A second compressible chamber – the circumferential deep scleral plexus

#### 3.7.1. The circumferential deep scleral plexus (CDSP)

**Fig. 19.**
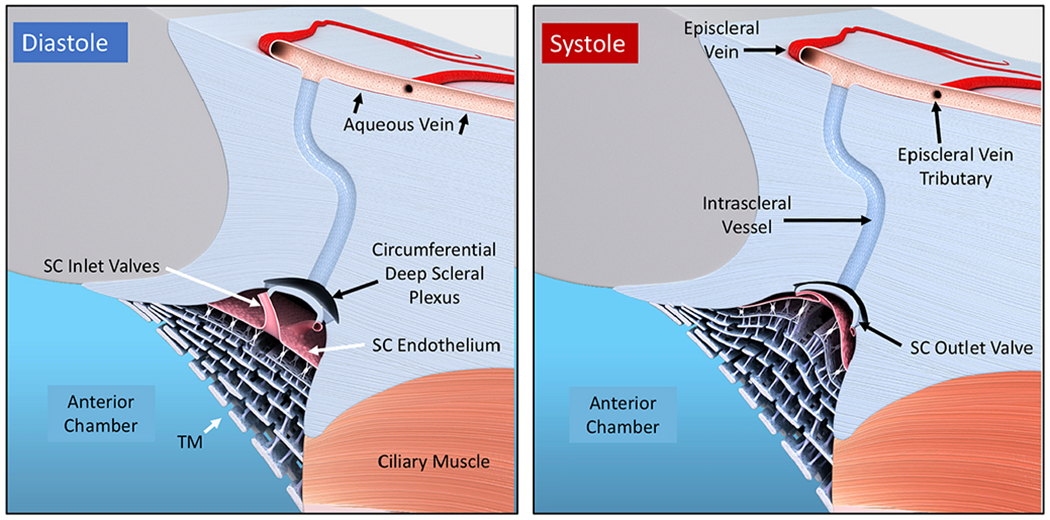
Aqueous pump model incorporating valves and compressible chambers. From the resting state in diastole, systole induces an intraocular pressure (IOP) rise, causing an ocular pulse wave in the anterior chamber (AC). The trabecular meshwork (TM) distends as IOP rises. TM distention causes Schlemm’s canal (SC) to narrow, reducing its volume, and forcing fluid through the collector channel (CC) entrances into the circumferentially oriented deep scleral plexus (CDSP). The SC outlet valve entrances close, followed by septa movement outward forcing aqueous out of the CDSP. During TM recoil in the next diastole, aqueous flows into the intertrabecular spaces, into SC through the conduits of the SC inlet valves and into CDSP. The cycle then repeats. The proposed anatomic relationships and pressure-dependent sequences are provisional and warrant further study. From Johnstone Glaucoma Laboratory, University of Washington.

The presence of a second compressible chamber (CDSP) adjacent to SC has come into focus as a result of recently developed technologies (§4.2.4) (Figs. 18 and 19). Experimental manipulations of fresh tissue at the dissecting microscope provided initial awareness of a generalizable pattern of structure, geometric relationships, and compressibility of the CDSP lumen (Fig. 10). Additional assessments then followed with histology (Fig. 17), phase contrast, differential interference contrast, and fluorescence microscopy (Fig. 11) (Jamil et al., 2010; Curtiss et al., 2011; Johnstone et al., 2011a)abs. To further explore relationships, we developed a technique of clarification to use with microvascular casting (Fig. 18) (§4.2.4) (Martin et al., 2012)abs (Carreon et al., 2017).

Studies using the dissecting microscope, serial histologic sections, SEM, and microvascular casting reveal that the CC lumen does not regularly connect directly to radial intrascleral channels leading to the episcleral veins but is slightly offset. The long thin septa between SC and CDSP are highly mobile, opening and closing the CDSP lumen with pressure changes. When the CDSP lumen is widely open, oblique sections may capture a communication that appears to connect the CC and distal channels.

However, close inspection reveals the offset, so when the CDSP lumen is closed, aqueous is prevented from entering the offset more distal lumen (Carreon et al., 2017). When the septum closes the CDSP lumen, it can act as a hinged flap valve at the CC entrances, but also as a valve that controls access to multiple, more radially oriented distal channels. The capability to act as a valved system is apparent from microvascular casts showing the continuity of CDSP lumen with the CC’s lumen and the entrances to the radially oriented distal channels (Figs. 18 and 19) (Carreon et al., 2017).

During experimentally controlled IOP changes, OCT’s image acquisition captures pressure-dependent distension and collapse of the CDSP (Fig. 16) (Vid 5 and 7). The change in CDSP height occurs at the same millisecond-range speeds as SC dimensions (Hariri et al., 2014). Full-thickness ribbon-scanning confocal microscopy (Waxman et al., 2018a) confirms the presence of CDSP. Studies with 3D micro CT also identify short narrow CCs that connect to channels running parallel to the limbus (Hann et al., 2011, 2014).

The distal valved pathways appear to be a prestressed, tensionally balanced system able to respond to oscillatory pressures and transients. Because pressures distal to CDSP are lower than in SC, pressure gradients force the septa into the lumen of the CDSP favoring closure. The septa at CDSP connect to the TM through the SC inlet valves. Elastance properties of the TM provide counterbalancing tension on the septa that favors retention of an open CDSP lumen. The distal valved system is then poised to respond to cyclic pressure changes, allowing it to act as a second circumferentially arranged valved chamber providing aqueous access to both CC and radially oriented distal intrascleral channels. Although we present peer-reviewed evidence to support the above proposal, it remains a premise that requires further study.

#### 3.7.2. Septa as outlet valves controlling flow into CDSP and distal channels

The septa’s unique morphology that permits its motion enables the CDSP lumen to act as a compressible chamber and a valving system. The long, thin, collagenous septa span the distance between adjacent CC entrances. The septa can move outward, closing CC entrances that lead to CDSP and, at the same time, close radial scleral channels leading to episcleral veins (Fig. 18). Our evidence demonstrates that septa respond to pressure gradient changes within the physiologic range, just as the TM does (Xin et al., 2016) (Fig. 16). The SC inlet valves that span SC create tension on the septa, causing the CDSP to open as is apparent from inducing ciliary muscle tension (Fig. 10) or reversing pressure gradients within SC (Fig. 11).

In addition to pressure-induced motion, perivascular smooth muscle components are present in SC, CC, and the CDSP (Gonzalez et al., 2012, 2013, 2016, 2017, 2014). The smooth muscle in the area may have enough contractile capability to provide additional modulation of aqueous outflow. Recent evidence demonstrates these regions are not encased in a rigid collagen tunnel but can move freely (Hann and Fautsch, 2009; Harm et al., 2011, 2014; Hariri et al., 2014; Xin et al., 2016) providing additional reasons to propose contractile behavior in this region is a factor in the regulation of aqueous flow.

The regular presence, organization, and behavior of the septa and CDSP are consistent with a functional role in controlling aqueous outflow. The SC lumen undergoes abrupt, substantial volume changes in response to pressures from blinking and especially forced blinking. The septa can serve a function as valves controlling aqueous entry to CC and radial intrascleral channels. The arrangement may explain residual outflow resistance after MIGS (§3.8.5). The premises of the septa functioning as complex valves regulating flow at CC and flow from CDSP into more distal pathways have support from peer-reviewed evidence noted above but need further validation. The CDSP chamber may also function as a Windkessel. Such a mechanism could reduce substantial pressure and flow fluctuations present in SC to a lower level in the distal pathways, thus modulating wall and shear stress. The aorta has a similar function in reducing pressure amplitudes in the distal circulation (Levick, 2010). Other possible functions could be a role as a hydraulic amplifier, a fluidic oscillator, or behavior similar to a microfluidic logic gate.

### 3.8. Laboratory pathophysiology – aqueous pump failure in glaucoma

#### 3.8.1. Studies reveal ~ 27 % of resistance is within the TM at physiologic pressure

**Fig. 20.**
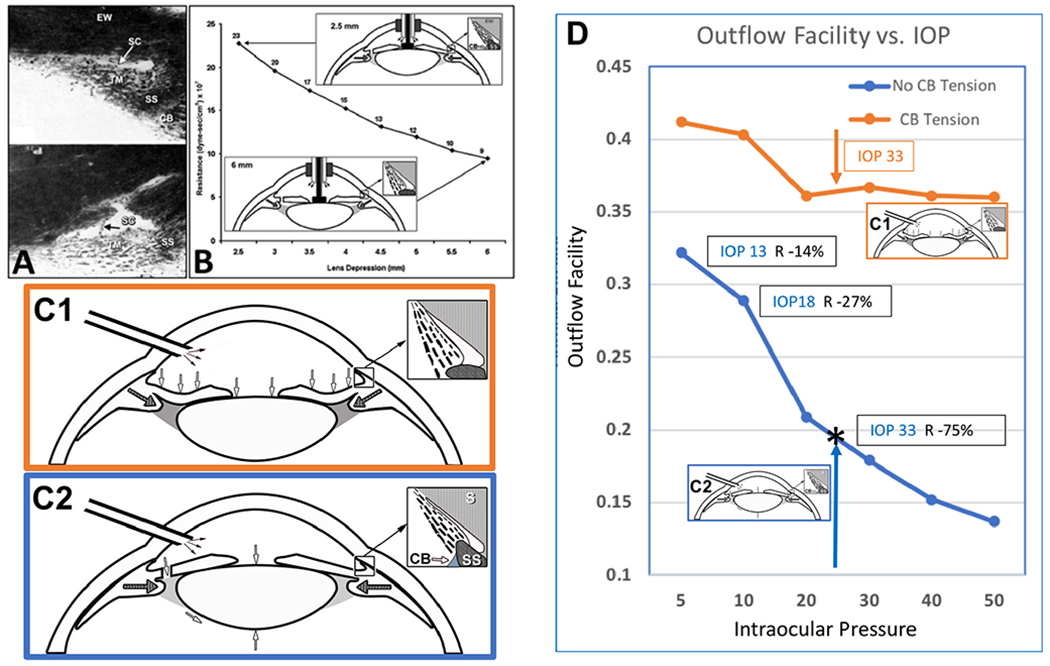
Ciliary muscle tension, intraocular pressure (IOP) and TM removal: Impact on outflow facility. **(A)** Crystalline lens backward movement dilates Schlemm’s canal (SC) and reduces resistance. The upper panel is with no lens depression. The trabecular meshwork (TM) is in extensive apposition to SC external wall (EW), causing the closure of SC lumen (arrow). In the lower panel, with depression of the crystalline lens, the ciliary body (CB) and scleral spur (SS) rotate posteriorly, pulling the TM attachments away from SC external wall. The TM distends, and SC lumen is large. The black arrow demonstrates the SC inlet valve extending from the TM to a hinged flap at a collector channel entrance. **(B)** The corneal perfusion fitting contains a lens-depression device. The CB and SS move backward with lens depression resulting in the opening of SC. As the lens moves backward, causing CB tension, resistance falls by over 50%. **(C1)** No iridectomy. Anterior chamber perfusion forces the lens backward, resulting in a reverse pupillary block phenomenon. Backward movement increases zonular tension that transmits to the ciliary body, scleral spur (SS), and TM tendons. The tension and vector forces cause the scleral spur and TM to move posteriorly and inward. In **(C2)** with an iridectomy, pressure gradients equalize between the anterior and posterior chamber eliminating posterior lens movement, so no tension is exerted on the ciliary body. **(D)** Outflow facility experimentally controlled at a series of steady-state intraocular pressures. The blue curve is outflow facility with no ciliary body tension, as shown in the C2 protocol. The orange curve is outflow facility with ciliary body tension resulting from the protocol, as shown in Cl. Pressures in the abscissa need to be adjusted upward by 8 mm Hg to reflect transtrabecular in vivo pressure gradients because of the lack of episcleral venous pressure in the ex vivo setting. Ellingsen and Grant determined reduction of outflow resistance from TM removal at each of the pressures noted in the blue curve of C2. As indicated by the boxed data, an effective IOP of 13, 18, and 33 mm Hg, trabeculotomy reduces resistance (R) by 14%, 27%, and 75%, respectively. The upward-pointing blue arrow and asterisk indicate the conditions of Grant’s initial 1958 and 1963 studies. With simulated ciliary muscle tension as in condition C-2, the outflow facility is initially higher than under condition (C-1). The facility of outflow remains high despite increasing IOP. The authors conclude that it is an apposition of the TM to SC external wall that causes increased resistance with pressure. They also reach the conclusion that ciliary muscle tension prevents SC wall apposition and a decrease in outflow facility. **(A)** From Van Buskirk M, Anatomic correlates of changing outflow facility. Invest Ophthalmol Vis Sci 22, 625–632, 1982. **(B)** From Johnstone, Pump failure in glaucoma. Essentials in Ophthalmology: Glaucoma II. Springer, Heidelberg, 2006. **C)** From Ellingsen and Grant, IOP and aqueous outflow, Invest Ophthalmol 10: 430–437, 1971.

Twentieth-century studies reveal that the TM accounts for only a modest portion of resistance (Fig. 20); such studies challenge Leber’s 19th-century passive filter hypothesis of IOP control by an area of blockage within the TM. The conclusion that TM is responsible for 75% of normal and elevated resistance in glaucoma is based on Grant’s early studies (Grant, 1958, 1963). The early studies were performed in ex vivo eyes at an IOP outside the physiologic range, in the absence of ciliary muscle contractile tone, and without histologic confirmation. The study’s experimental conditions severely limit the ability to rely on the finding’s conclusions, limitations that Grant’s own later studies acknowledge and correct. The concept entered textbooks and persisted despite Grant’s later work that rejected his initial conclusions. Because many studies continue to rely on the premise that control of resistance is in the TM, it is especially important to look at the underlying evidence.

Use of pressures in the normal range, simulating ciliary muscle tone, looking at histologic effects of the procedure, histologic effects of pressure on TM behavior, or removal of SC canal external wall, led Grant and colleagues to conclude that little resistance is in the TM itself. Instead, the body of evidence from Grant’s studies indicates the resistance is a result of TM motion that leads to SC wall closure and herniation into the entrances of CC (Ellingsen and Grant, 1971a, 1971b, 1972; Johnstone and Grant, 1973a, 1973b; Van Buskirk and Grant, 1973, 1974; Johnstone, 1974, 1979; Van Buskirk, 1976, 1982).

PhS-OCT studies in ex vivo whole eyes show SC closure and rapid reduction of TM motion as IOP increases (Li et al., 2012). The studies cited above correlate well with clinical science studies showing a reduction in pulsatile flow and TM motion in glaucoma. The studies also partially explain why MIGS procedures do not achieve pressure near episcleral venous pressure levels, as expected if the TM was the primary source limiting resistance to flow (Parikh et al., 2016).

#### 3.8.2. SC transient closure – A continuously recurring physiologic event?

It is essential to contrast transient closure of SC lumen by the TM, likely a regularly recurring physiological event, from the chronic, persistent TM distention/herniation into SC in glaucoma. Studies in nonhuman living primates with IOP experimentally controlled at 22- or 25-mm Hg demonstrate areas of extensive SC wall apposition (Johnstone and Grant, 1973a; Grierson and Lee, 1974a). Mean IOP in the human population is ~16 mm Hg, with blinking or eye movement inducing 10 mm Hg transients several times a minute (Coleman and Trokel, 1969). OCT imaging demonstrates that the TM experiences rapid motion, closing, and opening SC in milliseconds in response to pressure changes (Fig. 16).

Experimentally controlled pressures in non-human primate eyes fixed in vivo provided the first demonstration that the TM herniates outward, both coming into apposition with SC external wall and occluding SC entrances. The conditions were under steady-state pressure elevation (25 mm Hg) that is above the normal IOP setpoint (Johnstone and Grant, 1973a). Using the same protocols, the first demonstration of actual herniation into CC entrances in vivo was in 1974 (Grierson and Lee, 1974a), a finding later replicated in ex vivo human eyes (Hann et al., 2014). The evidence suggests that transient SC closure is normal in vivo when transients cause IOP to rise beyond the physiologic setpoint. The evidence also suggests that the TM lamellae elastance quickly results in recoil so that herniation is not persistent in vivo in normal subjects (Figs. 10 and 13).

#### 3.8.3. Persistent SC closure and TM herniation into CC – SC block glaucoma?

Evidence suggests patients with open angles may have a form of “SC block” glaucoma analogous to pupillary block glaucoma in those with shallow anterior chambers. Reports document persistent SC wall apposition, adhesion, and herniation into CC in glaucoma eyes, unlike the transient appositional events that appear to occur in normal eyes. A study in 2792 postmortem human eyes found apposition and adhesion between the TM and SC external wall, herniations of the TM into CC entrances, and involutional changes in distal channels. The findings were highly age-dependent, more frequent, and extensive in glaucoma eyes (Teng et al., 1955).

Another study of histologic sections through the entire circumference of a glaucoma eye identified 80% obstruction of SC lumen with many distal channels obliterated by the same pathologic process. Reactive changes found in the more distal outflow channels included proliferation and swelling of endothelial cells, degeneration of the collagen around the channels, and obliteration of the distal channel lumen (Chi et al., 1957). Similar findings of SC lumen closure and distal pathway occlusion were found in Dvorak Theobald’s study ((Teng et al., 1955; Dvorak-Theobald and Kirk, 1956; Chi et al., 1957).

In summary, clinical and laboratory findings are consistent with the assessment that TM motion initially slows, progressing to complete loss of motion, SC wall apposition, adhesion, and shear stress-related abnormalities in the distal outflow system, as glaucoma progresses.

#### 3.8.4. Combined SC block and distal channel glaucoma: MIGS failure cause?

MIGS, as well as laser procedures and medications, will be ineffective when the distal outflow pathways are no longer patent. Many shear stress related signaling processes are necessary to maintain normal patency of vascular pathways such as those of the distal outflow system (§5.2); such pathways no longer experience normal flow in the presence of persistent SC wall apposition and CC blockage.

In the systemic vasculature, inflammatory changes and remodeling are a known concomitant of aberrant shear stress signals, including those of TGFB and TNF alpha, when there is reduced flow in the systemic vasculature (Humphrey, 2002). Shear stress has long been recognized as integral to the aqueous pump model because of evidence of pulsatile flow synchronous with that of the systemic vasculature (Johnstone, 2004). In the model, shear stress abnormalities are considered a potential cause of IOP elevation in glaucoma (Johnstone, 2004, 2006).

#### 3.8.5. MIGS mystery –distal resistance – A residual intact distal valve system?

MIGS procedures may cleave open the TM, viscodilate SC, or bypass the TM with either a trabecular bypass stent or scaffold. However, these procedures may not address issues at the level of the CDSP (§3.7.2). When removing the TM, we may leave behind an intact distal valve system that is no long stented open by attachments to the TM.

An SEM study that separated SC walls found nineteen CC with simple oval openings with mean diameters of 54.8 ± 4.6 μm with no evidence of any overlying flaps (Bentley et al., 2016). Such large dimension openings should provide an IOP level close to that in the episcleral veins, which is about 7–8 mm Hg (Parikh et al., 2016), but clinical studies find pressure typically in the mid-teens. Why is pressure not lower when we see such large openings? The answer may lie in the organization of the septa and CDSP acting as a highly organized set of pressure-dependent mechanical valves not reliably accessed during SC-directed MIGS procedures §3.7.2.

MIGS procedures that remove the TM also eliminate the tethering or stenting forces that hold the distal valved system open. The persistence of a distal valved system after MIGS procedures may help to explain residual distal resistance. However, during the TM’s removal, some septa at CC entrances can also be torn away, variably exposing some radial channels distal to the CDSP (Johnstone and Grant, 1973b). Variable exposure of the radially oriented channels distal to the CDSP may partially explain variability in MIGS procedure success.

#### 3.8.6. Stroke volume - measure of pump efficiency or failure

In the systemic vasculature, stroke volume provides a measure of pump efficiency (Levick, 2010). Similarly, the amount of aqueous entering an episcleral vein with each pulse wave is the stroke volume delivered from SC to the vein by the aqueous outflow pump. Stroke volume decreases and is eventually undetectable as glaucoma progresses (Ascher, 1961). The cardiovascular literature typically discusses principles related to stroke volume efficiency from the heart and arterial circulation perspective.

However, pulsatile flow optimization in the return loops of the circulation involving veins and lymphatics are subject to the same principles and functional analysis (Levick, 2010). Parameters that determine stroke volume provide a framework for placing structural and functional abnormalities in the eyes of patients with glaucoma. Stroke volume determinates include choroidal vascular resistance, scleral wall compliance, scleral spur length, ciliary muscle tension, TM elastance, and EVP (Johnstone, 2006; Johnstone et al., 2010, 2011c). An additional parameter subject to pharmacologic manipulation is the elastance of the cytoskeletal elements of the endothelial cells that compose the walls of SC inlet valves (Hariri et al., 2014; Xin et al., 2016, 2017) (Jamil et al., 2010; Martin et al., 2012)abs.

Video imaging at defined frame rates and calibration by a micrometer provides time and dimension information to calculate stroke volume (Stepanik, 1954). Stroke volume depends on the efficiency of the pump (Hariri et al., 2014; Xin et al., 2016, 2017). In the outflow system, it is the aqueous outflow pump that must drive pulsatile flow. Changes in TM lamellae motion govern changes in stroke volume, which then regulates increases or decreases in pulsatile aqueous flow. PhS-OCT offers a new ability to measure TM motion that is the tissue machinery driving the pump. PhS-OCT can measure TM motion in living subjects (Li et al., 2013) and can quantitate the amplitude and velocity (Xin et al., 2018), suggesting that the technology holds considerable promise as a tool to manage glaucoma. (§2.3).

## 4. An aqueous outflow pump – defining technologies span three centuries

### 4.1. Nineteenth and twentieth century

#### 4.1.1. Slitlamp – crucial technology identified pulsatile flow into the aqueous veins

Histologic studies in the 19th Century resulted in the proposal that IOP control was at an area of blockage in the region of SC endothelium (Leber, 1873). Slitlamp biomicroscopy in human subjects is a crucial 20th-century technological advance permitting clinician-scientists to discover and study flow into the aqueous veins at high magnification in real-time. The findings challenge Leber’s passive filter hypothesis but instead point to a pump-like model. The clinician-scientists’ many studies demonstrate that pulsatility is a salient feature of aqueous outflow and becomes abnormal in glaucoma. Since the observable behavior is grounded in physical reality, it provides a foundation for interpreting functional behavior within the outflow system.

To dispel any doubts, 21st-century digital videography and the internet make direct observation and verification of pulsatile aqueous outflow available to everyone. Reports in the early 1970s provided laboratory evidence of a pump mechanism within the outflow system consisting of a movable wall, compressible chamber, and valved structures (Johnstone and Grant, 1973a; Johnstone, 1974). Recognition of the connection between the internal pump components and pulsatile flow in the aqueous veins was slow and awaited a report thirty years later (Johnstone, 2004).

#### 4.1.2. Gonioscopy – technology identifying pulsatile flow into SC and CC

Stegmann’s technology uses videomicroscopy, an 80-power microscope, and a large goniolens that can manually create episcleral vein compression (Johnstone, 2004). Under direct visual control, just enough pressure is placed on the goniolens to cause blood to enter SC, but at the same time, manual pressure adjustment with visual feedback creates an equilibrium that allows entry of aqueous to the canal. The technique effectively changes the location of oscillatory blood and aqueous mixing from the episcleral veins to the SC level. The pulse-induced ballistic head movement that is obvious at the high magnification identifies the cardiac pulse timing. Aqueous is the only clear fluid in the outflow system available to induce these phenomena, ensuring an accurate interpretation of the aqueous contribution to the findings (Figs. 4 and 5). The same technique permits direct observation of oscillatory flow in the CC that is synchronous with the ocular pulse.

#### 4.1.3. Manipulation of SC valves in the operating room

Stegmann developed a technique using his 80-power microscope in the operating room to create a scleral flap and elevate the outer wall of SC. The elevation of the scleral flap to expose SC causes the aqueous valves to stretch between the inner and outer walls of the canal. He identifies the valves as semitransparent tubules with a lumen. He describes properties very different from collagen because of their ability to stretch and recoil. He elevates the SC inlet valves using a delicate instrument, stretching them until they finally burst and discharge aqueous from their lumen.

#### 4.1.4. Dilation of SC: key to a previously hidden world of structure and function

In the 20th Century, it became apparent that SC dilation is essential to study outflow system structure and function. In studies in ex vivo eyes without ciliary muscle tone, the canal is little more than a potential space obscuring crucial structural features and relationships. *In vivo* fixation of eyes, under conditions of reversed pressure gradients, opened a window to a previously unseen world of structures within SC (Johnstone and Grant, 1973a; Johnstone, 1974). Techniques of SC dilation were used to study relations documented in Figs. 8–12 and 14–17 and 22. Reports of Grierson and Lee confirmed the advantages of living non-human primates to reveal SC relationships by SC dilation (Grierson and Lee, 1975b). However, such studies in living primates are unduly complex, and fixation results in the canal always being full of blood, preventing a direct view of structural features. Appreciation of structures and relationships required numerous serial sections oriented in multiple planes. The combination of challenges discouraged ongoing studies.

In 2000, viscoelastic agents became available that reliably dilate SC while undergoing fixation. We were pleasantly surprised to find that the viscoelastic then washes out of the canal after fixation. The discovery allowed us to study topographic relationships by SEM (Smit and Johnstone, 2000, 2002). Further, SEM studies revealed the SC inlet, outlet valves, CC entrances, and the compressible ancillary chamber represented by the CDSP (§3.5–3.7). However, the ability to study motion was still not within our grasp.

### 4.2. Twenty-first century

#### 4.2.1. OCT study of dynamic motion of outflow structures

We recently developed a platform to examine and image the outflow tissues’ pressure-dependent motion using high-resolution OCT (Hariri et al., 2014). We control SC lumen dimensions by cannulating the canal and attaching the cannula to reservoirs. OCT imaging, coupled with the ability to change pressure gradients, finally permits varying SC dimensions to study dynamics of the tissue machinery controlling aqueous flow. OCT is of unique value because it provides unprecedented details of structures, relationships, and motion that provide an evolving picture of functional behavior. The techniques also add new, unexpected findings. Such findings include the ability to observe SC outlet valve leaflet movement controlled by attachments to the TM and septa movement that controls the CDSP lumen dimensions.

#### 4.2.2. Direct observation of outflow system behavior in unfixed tissue

Using a similar phenomenological approach to clinical studies, we use the dissecting microscope to perform manipulations on outflow tissues immediately following death. The technique provides the means to assess motion in the same sample, both during distention and recoil. Real-time videography provides a permanent record of structural images (Fig. 4) and motion (Vid4). It also provides a quantitative mechanism to study tissue structure and motion using a micrometer scale and time calculations based on frame rates (Fig. 5) (§5.3) (Johnstone et al., 2014a)abs.

Direct observation and manipulation of unfixed tissue eliminate the need for sophisticated, expensive laboratory techniques that are the province of only a small number of investigators. Direct observation also circumvents the many assumptions associated with indirect measurements. SC inlet valves can be particularly challenging to identify because they are oriented circumferentially in SC and are transparent when viewed against a bright background under a dissecting microscope. Their presence is not evident during dissections and in routine histology studies that do not involve SC dilation. A technique described below permits untrained observers to identify and study the SC inlet valves at the dissecting microscope without difficulty.

#### 4.2.3. Microscope observation of SC inlet valves, outlet valves, and their motion

The technique involves preparing radial sections of the limbus ~250 μm in thickness, taking care to avoid separation of the ciliary muscle from the scleral spur. Blue or black tape pinned to a paraffin layer in a Petri dish provides a contrasting background for viewing. Pins, passing through the cornea and sclera, fix the tissue to the paraffin underlying the background tape. Oblique light then directed at the cornea acts as a light pipe, making all outflow structures, including semitransparent ones, visible (Fig. 10) (Vid4) (Johnstone, 2016) (Curtiss et al., 2011)abs (Johnstone et al., 2019)abs.

Forceps place backward tension on the ciliary muscle that transmits to the scleral spur and the TM lamellae via the trabecular tendons. The TM lamellae attached to both the scleral spur and ciliary muscle-TM tendons move posteriorly. Tension on the lamellae pulls them posteriorly and toward the center of the eye causing SC to dilate. The resultant tension dilating SC creates tension on the SC inlet valves that, in turn, transmits to the SC outlet valves and septa associated with the CDSP.

Motion, connections, and changing lumen dimensions are easy to visualize in real-time (Vid5). Pulse waves of fluid from a cannula directed at the TM causes it to distend and recoil. Gentle suction from a syringe attached to a cannula placed against the TM permits traction that causes controlled dilation of SC, revealing SC inlet valves, outlet valves, and their relationships.

#### 4.2.4. Through-corneal viewing of CDSP – clarification, microvascular casting

The initial recognition of CDSP and their relationships resulted from manipulations of fresh tissue at the microscope and by reviewing SEM preparations after the viscoelastic dilation of SC. Each of those techniques used radial or meridional limbal segments. To better explore the issue in intact limbal segments, we developed a clarification technique new to ophthalmology involving benzyl alcohol/benzyl benzoate (BABB) (Martin et al., 2012)abs, and a microvascular casting technique involving SC injections. We also developed a technique to examine the CC relationship to CDSP using a view through the cornea’s cut surface (Fig. 18).

The relationship of SC, CC, and CDSP is not evident in microvascular casts viewed from the corneoscleral surface. The CC connections to the CDSP are in an axial plane lying directly over the much larger SC cast that is the same color. The axial view does not permit examining sections at an angle perpendicular to the CC exit from SC. We discovered that images through the cut cornea provide a view perpendicular to the CC connections to CDSP.

With through-cornea viewing, we found that the CC exit SC in a very consistent plane relative to the limbal circumference and enter the CDSP at a consistent distance from SC lumen. We also found that CC regularly enter the CDSP rather than passing directly to the surface.

The path length along the CDSP is at times short before entering the radial channels. However, the CC entry site to the CDSP and the distal channels originating from the CDSP are not directly opposite each other; instead, they are slightly offset. When the septum closes the CC entrance to the CDSP, the septa can behave in a valve-like fashion preventing direct flow from the CDSP to the distal pathways even though a direct connection appears visible when the CDSP lumen is open (Fig. 18).

A single oblique histologic section or one dilated by microvascular casting material may capture the CC, a slight deviation along the CDSP path, and then the more radially oriented vessels coursing to the surface. Without a technique able to document their 3D appearance perpendicular to the exit site, such oblique sections may lead one to think that aqueous does not first pass circumferentially along CDSP before entering the more distal radial pathways. A small angulation that results in wall apposition with CDSP lumen collapse is all that is necessary to prevent distal flow.

#### 4.2.5. PhS-OCT TM motion studies in ex vivo non-human primates

PhS-OCT represents a 21st-century technological advance that can measure TM motion at the nanometer level in whole eyes. The technique differs fundamentally from SD-OCT because it measures motion instead of microstructure. The technique uses sensitive phase information within the OCT signals (Wang et al., 2006, 2007; Wang and Nuttall, 2010). Techniques of SD-OCT do not offer the ability to measure the TM movement from the sclera’s surface because the movement is often ≤ four μm, a resolution the techniques cannot achieve.

In ex vivo eyes, we mimicked the in vivo ocular pulse using an anterior segment perfusion system that provides sinusoidal IOP oscillations. Using the PhS-OCT approach developed in Dr. Wang’s laboratory, we captured real-time video images of dynamic TM movement in situ. The technique permits correlating IOP with structural motion, TM velocity, displacement, and strain rate with ~20 nm sensitivity. Simultaneously, the OCT system images the SC structure (Li et al., 2012).

In the ex vivo eyes, we studied whether physiologic pulse amplitudes can induce TM motion by mimicking amplitudes (3 mm Hg) and frequencies (60 cycles per minute) found in vivo under changing pressure conditions. The detected TM movement was highly synchronous with the experimentally induced ocular pulse and induced TM displacement amplitudes of 3–4 μm. Calculations indicated that the amplitude and frequency of pulse-induced TM excursions into the canal result in enough volume changes to account for the total volume of aqueous outflow (Li et al., 2012). This study was the first to identify and characterize the TM’s dynamic real-time pressure-dependent motion.

By using IOP as the independent variable while maintaining a constant pulse amplitude of 3 mm Hg, we identified an exponential decrease in the amplitude of pulsatile TM motion as IOP increased. Images from the same tissues overlaid with the corresponding SD-OCT images demonstrated a marked reduction in SC lumen size with progressive apposition between SC walls (Li et al., 2012).

The findings suggest that as pressure rises, a vicious cycle of decreasing SC size, increasing IOP, and reduced ability to discharge aqueous by pulsatile mechanisms can develop. Clinician scientists have articulated recognition of and evidence for such an undesirable feedback loop (Schirmer, 1969, 1971; Suson and Schultz, 1969). However, the earlier papers do not address the more complex metric of compromised pulse-dependent TM motion.

#### 4.2.6. PhS-OCT TM motion studies in humans

For in vivo use in humans in a clinical setting, we developed another novel purpose-built PhS-based OCT system (Li et al., 2013). The captured images contain both structural and phase information. The simultaneous real-time capture permits displaying color-coded instantaneous velocity and displacement data overlaid on structural images for better visualization and interpretation (Xin et al., 2018) (Fig. 6). The resolution of motion is high enough to identify differences in motion between the TM’s internal and external regions with good reproducibility.

#### 4.2.7. Aqueous angiography – noninvasive and invasive techniques

Noninvasive aqueous angiography uses the hemoglobin absorption spectrum to increase the contrast between red blood cells and the surrounding plasma (Khatib et al., 2019). The technique is easily adapted for use at the slit lamp in a clinical environment and can provide real-time high-resolution videos, identify the pulsatile aqueous flow, and quantitate flow rates.

Invasive aqueous angiography in the operating room is another evolving technology. The first report described dyes introduced into the outflow system in ex vivo bovine (Huang et al., 2016a) and human eyes (Saraswathy et al., 2016). The studies provide evidence of the pulsatile behavior of aqueous flow in vivo in non-human primates (Huang et al., 2017b) and humans (Huang et al., 2017a).

#### 4.2.8. 21st century – shear stress and nitric oxide in the aqueous outflow system

Biomechanical coupling through pressure and flow-mediated mechanisms has a long history of acceptance in the vascular system (Levick, 2000). However, the prevailing paradigm in the aqueous outflow system literature posits a sufficiently rigid syncytium of ECM material in the juxtacanalicular space to sustain a steady passive resistance that regulates both pressure and flow. Aqueous passage through a filter in a geometrically stable resistance-inducing region, then wide distribution through low resistance pores does not lend itself easily to an explanation of outflow system shear stress, especially in the distal outflow system.

The aqueous outflow pump model report in 2004 provided evidence of pulsatile flow speeds in the outflow system in human eyes like those in the systemic vasculature. The clinical evidence led to the conclusion that shear stress controlled outflow system lumen dimensions by mechanisms like those in the systemic vasculature, including recognition of the role of nitric oxide and endothelin (Johnstone, 2004).

## 5. Generalizable concepts of homeostasis related to the aqueous outflow system

### 5.1. IOP control – immediate biomechanical responses to an IOP change

The aqueous outflow pump behavior illustrates multiple hierarchal but integrated levels of pressure control that are well-studied in vascular physiology and biomechanics. The control mechanisms considered in this section posit that aqueous outflow tissue pathways rapidly deform in response to pressure changes, that the deformation provides sensory input, and that TM tissue motion is under the control of mechanotransduction mechanisms.

#### 5.1.1. Trabecular tissues exhibit properties of a pressure sensing system

The inner wall endothelium of SC and the tethered lamellae experience constant pressure-dependent stress as well as cyclic and transient stresses at physiologic pressures (Coleman and Trokel, 1969; Phillips et al., 1992). The TM tissues do not retain a static configuration while experiencing IOP-induced stress (Figs. 13–15). The SC inner wall endothelial sheet and the attached TM lamellae respond by moving, demonstrating deformation or strain. The numerous cytoplasmic processes tether the lamellae to each other, to juxtacanalicular cells, and to SC endothelium. The tethering creates the tensionally integrated system, as is indicated by stresses on all the cellular connections. The elastic fiber system is also able to provide synergistic tensioning.

The tensional integration ensures the distribution of the pressure-dependent stresses from the SC inner wall endothelium to all the TM’s cellular elements. The connections provide a tensionally-integrated, preconditioned, prestressed communicating system. The TM acquires properties like those of a baroreceptor through the close coupling of pressure and configuration (Johnstone and Grant, 1973a) (Fig. 8).

#### 5.1.2. The pump model – four synergistic short term IOP control mechanisms

##### 1) Pathway Dimensions Change in Response to Pressure-Sensing Mechanisms

First, the outflow pathway configuration, tightly linked to the IOP because of its tensionally integrated and prestressed properties, responds to small IOP changes with substantial configuration changes. A pressure increase or decrease of as little as 5 mm Hg from the neutral position seen in hypotony causes SC endothelium to undergo marked distension or recoil (Johnstone and Grant, 1973a; Johnstone et al., 1980). Enlargement of intertrabecular and juxtacanalicular spaces increases, providing larger spaces for aqueous flow. Schlemm’s canal, the SC inlet valves, outlet valves, and CDSP all change pathway configuration to accommodate increased flow. TM distention may also activate receptors in the scleral spur region, invoking ciliary muscle configuration changes (Tamm et al., 1994, 1995a, 1995b).

##### 2) A Rise in IOP Increases Pulse Amplitude, TM Motion, and Pulsatile Flow

A second regulatory mechanism is related to the linkage between IOP, pulse amplitude, and TM motion that determines stroke volume. As IOP rises, pulse amplitude increases linearly.

An increase in pulse amplitude increases TM motion, increases stroke volume (Phillips et al., 1992), increases aqueous flow, and decreases IOP. A reduction in pressure results in reduced stroke volume and increased IOP. The linkage provides an intrinsic mechanism of error detection that can serve to identify deviations and restore optimal pressure.

##### 3) A Rise in IOP Increases TM Distention and Can Open SC Outlet Valves

**Fig. 21.**
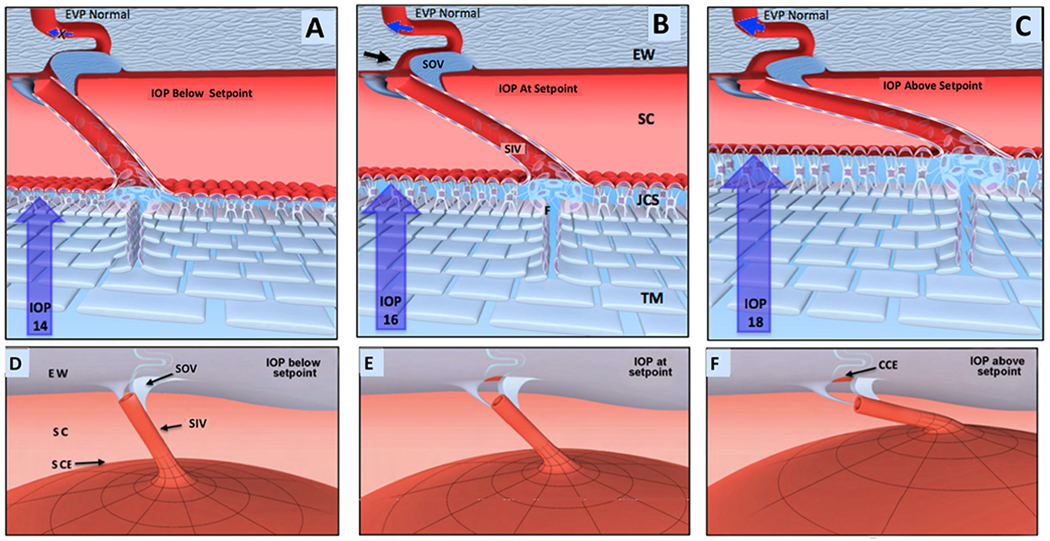
Provisional 2D outflow model for regulation of homeostasis. **(A), (B),** and **(C)** depict cross-sections through the outflow system while **(D), (E)** and **(F)** show global motion in three dimensions (3-D). Aqueous passes through the TM to the juxtacanalicular space (JCS). From the JCS, aqueous flows through the Schlemm’s canal (SC) inlet valves into SC. SC outlet valves (SOV), consisting of hinged collagen flaps, control collector channel entrance (CCE) dimensions. As Intraocular pressure (IOP) increases from low in **(A-D)** through the setpoint **(B-E)** to high in **(C-F),** the intertrabecular spaces and JCS enlarge, and SC narrows. In a 3-D view, the SIV, attached both to the TM and SOV, are oriented circumferentially in SC. Outward movement thus pulls the SOV open. **(B-E)** envisions an IOP of 16 mm Hg as an ideal homeostatic setpoint configuration. As IOP increases in **(C-F),** the TM moves outward. The SIV experience increased tension, resulting in increased stress on the SOV, causing it to open the CCE further. The CCE enlargement causes increased aqueous flow; IOP then falls with an associated inward movement of the TM, restoring it to the setpoint of **(B-E).** At a low IOP as in **(A-D),** the CCE is closed, reducing flow. Reduced flow increases IOP causing the TM to distend, thereby returning tension to the setpoint in **(B-E).** IOP generates forces causing TM tissues to deform and distend into SC. TM elastance properties balance the distending or loading force of IOP. TM tissues at the homeostatic setpoint are in an IOP-induced, prestressed, equilibrium state of deformation. Under homeostatic conditions of IOP > SCP > EVP, gradients favor closure of the HCF because SCP is higher than EVP. TM tension on the SOV, causes them to enlarge and optimizes CCE dimensions. Blue arrows in CCE depict changes in the rate of flow. Black arrow points to CCE. The proposed model is offered for consideration but is unproven, and its premises are subject to modification or rejection as new evidence emerges. From Johnstone M, IOP control through linked trabecular meshwork and collector channel motion. Glaucoma Research and Clinical Advances: 2016 to 2018. Kugler Publications, Amsterdam.

A third regulatory mechanism results from the connections coupling the TM and SC outlet valves; the connections are obliquely or circumferentially oriented within SC (Figs. 15, 16, 19 and 21) (Hariri et al., 2014). As a result of the oblique connections, the TM’s pressure-dependent outward movement can induce progressive opening of the SC outlet valves as pressure increases, providing another simple intrinsic mechanical control mechanism to reduce IOP (Johnstone, 2016) (Fig. 21).

##### 4) Pump Model – Integration of Shear Stress, Nitric Oxide, and Endothelin Mechanisms

A fourth short-term IOP control mechanism is the shear stress signaling system identified in the aqueous outflow pathways. When the flow is too fast, vessel lumen size increases; when the flow is too slow, vessel lumen size decreases; both responses result in a return of flow to the homeostatic setpoint. Clinical studies provide extensive evidence of pulsatile flow into SC, CC, and the aqueous veins from SC; the pulsations in the outflow and systemic vasculature are synchronous, providing a linkage to shear stress in the systemic vasculature (Johnstone, 2004; Johnstone et al., 2010; Khatib et al., 2019).

Rodbard’s report was the first to recognize shear stress mechanisms in the systemic vasculature (Rodbard, 1975). By 2002, textbooks ranging from introductory cardiovascular physiology to cardiovascular biomechanics contained explanations and illustrations of shear stress mechanisms (Levick, 2000; Humphrey, 2002). No comparable unifying framework was present in the outflow literature until the 2004 report describing an aqueous outflow pump (Johnstone, 2004).

The presence of aqueous flow synchronous with that of the systemic vasculature permitted the 2004 aqueous outflow pump report to conclude that the outflow system must have shear stress mechanisms like those in the systemic vasculature, including nitric oxide and endothelin signaling. The shear stress implications and lumen dimension control were incorporated as an integral feature of the aqueous pump model of homeostasis and reiterated in subsequent reports (Johnstone, 2006, 2009, 2014).

Another 2004 report describes the preferential alignment of SC endothelium cells that correlates with an increase in marginal F-actin in response to shear stress. The authors conclude through modeling that flow rates like those in the arterial system might be achievable in SC in the absence of pulsatile outflow mechanisms (Ethier et al., 2004). Recent laboratory reports support the premise of shear stress dependent nitric oxide signaling in living murine eyes and ex vivo in the distal pathways of porcine and human eyes (Stamer et al., 2011; Mcdonnell et al., 2018; Waxman et al., 2018b; Mcdonnell et al., 2020).

###### 5.1.2.1. Conduit-like behavior.

Close to the optimal IOP setpoint, the pump system can exhibit conduit-like behavior with limited oscillations (Fig. 3) (Vid 3). A similar dual, pump-conduit behavior is present in the lymphatics (Quick et al., 2007, 2009). Aqueous veins can exhibit oscillations at the homeostatic set point that involve minimal pulsatile flow. A progressive increase in IOP elicits larger oscillations in the pulse amplitudes with an increase in the pulsatile flow until the IOP returns to the previous homoeostatic setpoint, at which time minimal oscillations are again present (Fig. 3) (Johnstone et al., 2010) (Vid 3). Medications also induce an increase in stroke volume that persists until IOP decreases to a new lower medication-dependent setpoint.

### 5.2. Homeostasis – elastic energy optimization in cells and tissues

#### 5.2.1. Elastance

Elastance is a useful parameter in the eye because the eye is a hollow organ. Elastance is the ability of a hollow organ to recoil from a distending force; it reflects the stored 3-D strain energy of the tissues. In contrast, Young’s Modulus describes an object’s tendency to deform along an axis, its tensile elasticity. Elastance measurements play an essential role in understanding cardiovascular, pulmonary, and lymphatic physiology (Brown and Ditchey, 1988; Gattinoni et al., 2004; Quick et al., 2008). Elastance, also known as stiffness, plays a central role in the aqueous outflow pump model.

Elastance (E) describes the deformation of the walls of a chamber expressed as a change in pressure (P) per unit change in volume (V); E = P/V. In an unpressurized hypotonous eye, aqueous can be infused into the AC until it fills with aqueous, but there is no increase in pressure on the coats of the eye until that point. The aqueous volume infused before the eye fills and the coats begin to distend is the unstressed volume V0. Any further volume added begins to cause distending forces stretching the globe’s walls with a linked rise in IOP. The volume above V0 is the stressed volume (V–V0). SC endothelium behaves as the outer boundary of such a distensible container, distending, and recoiling in response to IOP changes (Johnstone and Grant, 1973a). Because the TM lamellae are tethered to the SC endothelium, they distend and recoil in unison with the endothelium (Hariri et al., 2014).

**Fig. 22.**
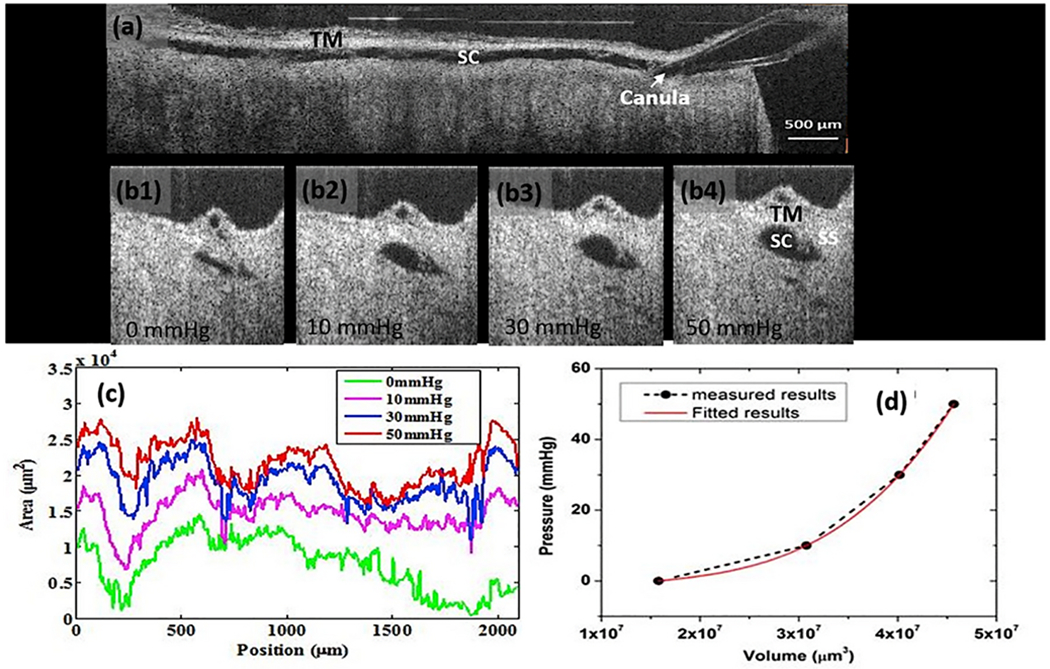
Trabecular meshwork elastance curve. OCT imaging quantifies the relationship of Schlemm’s canal (SC) pressure and volume over a range from 0 of 50 mmHg. **(a)** A composite cross-sectional image permits visualization of ~8 mm of a limbal segment while maintaining a perfusion pressure of 50 mm Hg. The perfusion pressure dilates SC along its entire length. A cannula is visible in SC at the right edge of the image. The trabecular meshwork (TM) is superior to the canal. OCT images **(b1-b4)** are radial cross-sections through SC. Images demonstrate progressive dilation of the canal as pressure increases from 0 to 50 mm Hg. **(c)** Curves represent SC instantaneous volume and pressure measured at 10 μm intervals along the 2 mm limbal segment with pressures as indicated, **(d)** Plotting the pressure increase against the SC volume generates an elastance curve. The curve captures the elastic energy increase resulting from an increase in SC volume. The increase in potential energy distributes between further TM tissue deformation and the rise in pressure. The elastance curve provides a means of assessing the ability of the TM tissues to store elastic energy. Elastance and stiffness are synonymous terms. Tissue source: Human Eye. From Xin C, Mechanical Properties of the TM and Collector Channels, PLoS One 11, e0162048, 2016.

The volume infused above V0 increases the volume of the elastic container defined by the TM tissues. Within the TM, the TM lamellae’s elastance primarily determines how far the tissues will distend into SC in response to a pressure increase. Clinical studies demonstrate that elastance-dependent TM motion is abnormal in glaucoma (§2.2.2). Elastance has been successfully measured in TM tissues by OCT in an ex vivo environment (Fig. 22) (Xin et al., 2016). Recent PhS-OCT advances permit TM motion measurements in human subjects, a reflection of elastance, suggesting elastance measurement may be useful in managing glaucoma.

#### 5.2.2. The equilibrium energy (information) state specifies IOP homeostasis

**Fig. 23.**
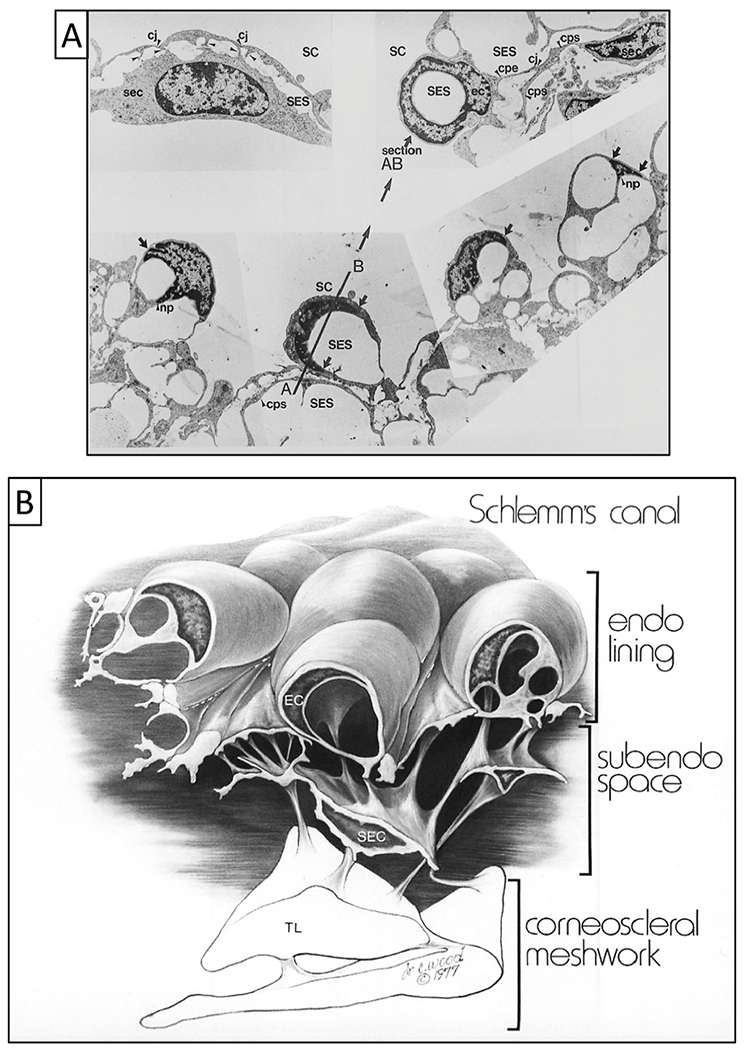
Synchronous pressure-dependent cytoplasm and nucleus deformation enabled by tethering. **(A)** Images are from transmission electron microscopy following in vivo fixation at an intraocular pressure (IOP) of 25 mm Hg with normal episcleral venous pressure (EVP). Schlemm’s canal (SC) endothelial cell cytoplasmic projections (arrowheads) join cytoplasmic processes of subendothelial cells (sec) bodies in the subendothelial space (SES). The terms subendothelial cell and juxtacanalicular cell are synonyms. The terms subendothelial space and juxtacanalicular space are also synonyms. Nuclei and cytoplasm of the SC endothelial cells distend into the SC lumen. The periphery of these distended, flattened nuclei taper (arrows). Nuclei frequently deform into a hollow-hemisphere shape and are tilted into many planes, resulting in frontal sections as illustrated by the plane through AB in the lower-left panel. A corresponding section (AB) in the upper right panel is a cylindrical nuclear profile surrounding the subendothelial space. The hollow circular nuclear profile is analogous to the appearance in the areas of cytoplasm that are referred to as “giant vacuoles.” Cytoplasmic processes originating beneath the nucleus of the endothelial cells join cone-shaped nuclear projections that appear as an inverted triangle in cross-sections (np). A cytoplasmic process from an endothelial cell (cpe) traverses the subendothelial space to join a cytoplasmic process (cps) of a subendothelial cell. f. 4650.) Tissue source: Primate, *Macaca mulatto.* (B) SC inner wall endothelial lining mirrors the appearance seen in the lower panel of (A). It illustrates the cytoplasmic processes that tether SC endothelium to the trabecular lamellae (TL) through cytoplasmic process connections of subendothelial cells. (A) from Johnstone M, Pressure-dependent changes in Nuclei, Ophthalmol. & Vis. Sci. 18, 44–51, 1979. Illustration from Johnstone Glaucoma Lab, University of Washington.

An increase in the stressed aqueous volume results in increasing distention of the entire SC endothelial sheet into SC (Johnstone and Grant, 1973a; Grierson and Lee, 1974a; Lee and Grierson, 1974; Grierson et al., 1978; Johnstone, 1979). Distention of the sheet results in the deformation of individual SC endothelial cells and the tethered TM lamellae complex (Johnstone, 1979). Tissue and cellular deformation are a manifestation of the cell’s storage of elastic energy, a form of potential energy. At physiologic pressures, the tissues and cells maintain a pressure-dependent steady-state deformed configuration. When the tissues and cells experience increased deformation in response to an increase in compartment volume, they accrue added elastic energy. Tensional integration permits the distending SC endothelium to activate the TM lamellae’s cytoskeletal linked signaling pathways (Fig. 23); wall stresses activate integrin-dependent pathways involving cadherins, focal adhesions, and stress-sensitive ion channels. The stress-induced cellular deformation also causes rearrangement of the cytoskeletal elements, including the intermediate filaments attached to the internal surface of the nuclear envelope (Sims et al., 1992).

**Fig. 24.**
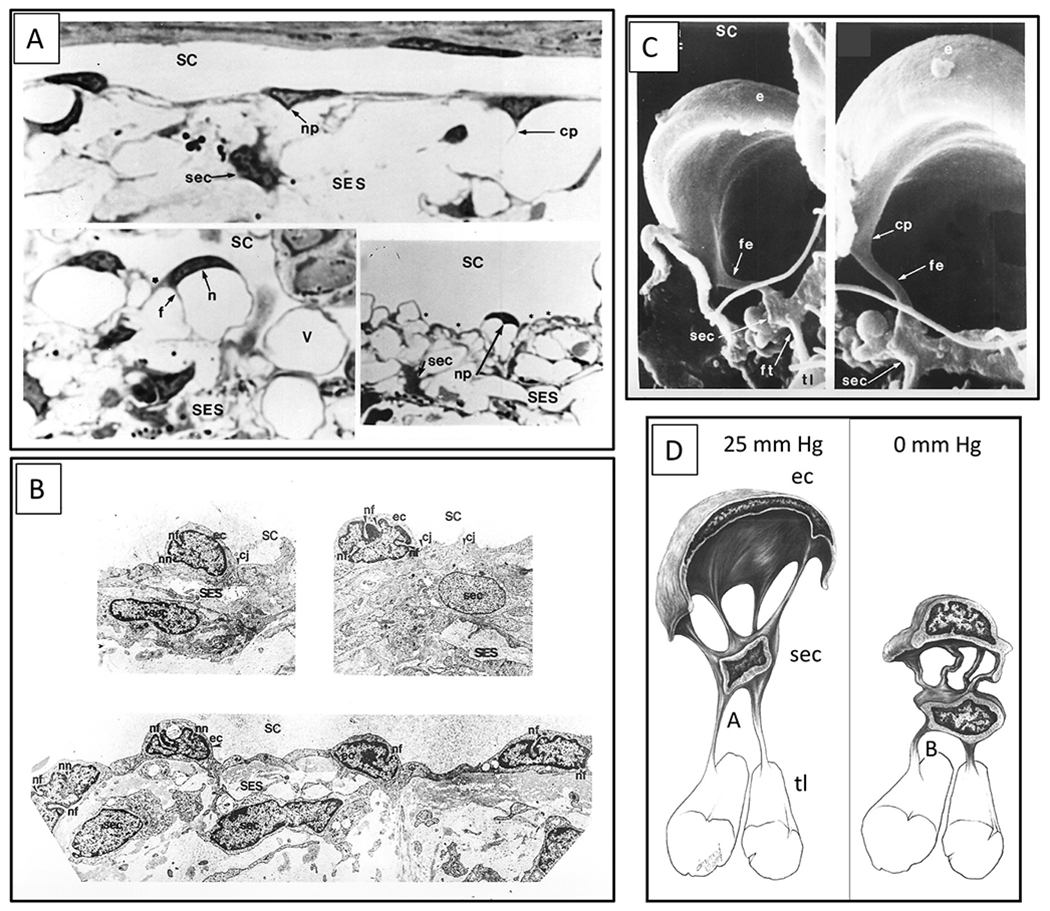
Tethering of Schlemm’s canal endothelium to the meshwork permits cell deformation. Images from non-human primate eyes following in vivo fixation. Image **(A)** and **(C)** represent the configuration with a positive IOP of 2525 mm Hg. Image group in **(B)** is the configuration with zero IOP but normal episcleral venous pressure. The reversed transtrabecular pressure gradient of 8 mm Hg results in TM collapse, Schlemm’s canal (SC) dilation, and altered cellular configuration. **(A)** Light microscopy. Upper image. Nuclei of Schlemm’s canal (SC) inner wall endothelium are flattened and elongated, tapering peripherally toward cytoplasmic elongations. At the origins of endothelial cell cytoplasmic processes (cp), the cytoplasm and nucleus (np) of the lining deform toward the subendothelial space (SES), creating a triangular projection in the two-dimensional image. The SES and juxtacanalicular space are synonyms. In the lower-left and right images of **(A)**, elongated nuclei (n) have the same shape as the cytoplasm, forming a partial circular profile. Distal ends of the nuclei taper, resulting in crescent-shaped structures in section. Sections through portions of the distended cells that do not include the nucleus result in cytoplasmic vacuole-like (V) structures. Triangular-shaped cytoplasmic processes of SC inner wall endothelial cells extend into the subendothelial space (SES). Depressions are present on the cytoplastic surface facing Schlemm’s canal (asterisk) opposite the origin of cytoplasmic processes consistent with tension exerted on the cell walls at cytoplasmic process attachment sites. A subendothelial cell (sec) is also known as a juxtacanalicular cell, (scale bar = 5 μm; ah ×2000.) In image **(C)**, left panel, scanning electron microscopy demonstrates an endothelial cell (e), forming a hemispherical profile. A cytoplasmic process (fe) originates from the endothelial cell and attaches to a subendothelial cell. A second process (ft) arises from another surface of the same subendothelial cell and joins a trabecular lamella (tl). In image **(C)** right panel, a cone-shaped area (cp) of the undersurface of the endothelial cell becomes a cylindrical cytoplasmic process (fe) as it extends to the subendothelial cell, (scale bar = 0.5 μm; left panel (×10,000); right panel, (×12,000). **(B)** Reversed transtrabecular pressure gradient of ~8 mm Hg. Transmission electron microscopy. Nuclei of the endothelial cells (ec) are rounded and bulge prominently into Schlemm’s canal lumen. Subendothelial cells are generally round. The nuclei have many deep nuclear folds (nf) and notches (nn); Endothelial cell junction (cj). (scale bar = 2 μm; ×4650.) **(D)** Schematic illustration of an endothelial cell (ec) lining Schlemm’s canal. The SC endothelial cell with its cytoplasmic processes attaches to a subendothelial cell (sec), which in turn attaches to a trabecular lamella (tl) via cytoplasmic processes. From Invest. From Johnstone M, Pressure-dependent changes in Nuclei, Ophthalmol. & Vis. Sci. 18, 44–51, 1979.

When IOP increases during pressure oscillations, the TM tissues deform and accrue increased pressure energy (Fig. 24). As the tissues recoil in response to lower pressure, the tissues and cells release the accrued elastic energy. The TM lamellae and the sheet of SC inner wall endothelial are prestressed oscillating around a mean homeostatic physical position at every instant in the pulse cycle. The tissues simultaneously maintain a vast information registry, the strain energy in the molecular bonds associated with the elastic energy-equilibrium setpoint. The setpoint becomes defined in the course of cellular differentiation by the evolutionarily determined signaling pathways that specify and optimize stresses within the cell membrane, cytoskeleton, nuclear envelope, and the chromatin (Kauffman, 1969a; Mazumder et al., 2008; Mazumder and Shivashankar, 2010).

#### 5.2.3. Instantaneous genomic signaling and responses to IOP changes

The aqueous outflow system behavior illustrates generalizable behavior present throughout the vascular system. A hierarchy of tissue, cellular, and chromatin level tensional integration govern homeostasis in outflow system tissues and cells (Maniotis et al., 1997; Alenghat and Ingber, 2002; Ingber, 2003a). The nucleus behaves as a load-bearing organelle able to transmit mechanical clues to the nuclear chromatin by changing its configuration (Dahl et al., 2008; Snedeker, 2014).

**Fig. 25.**
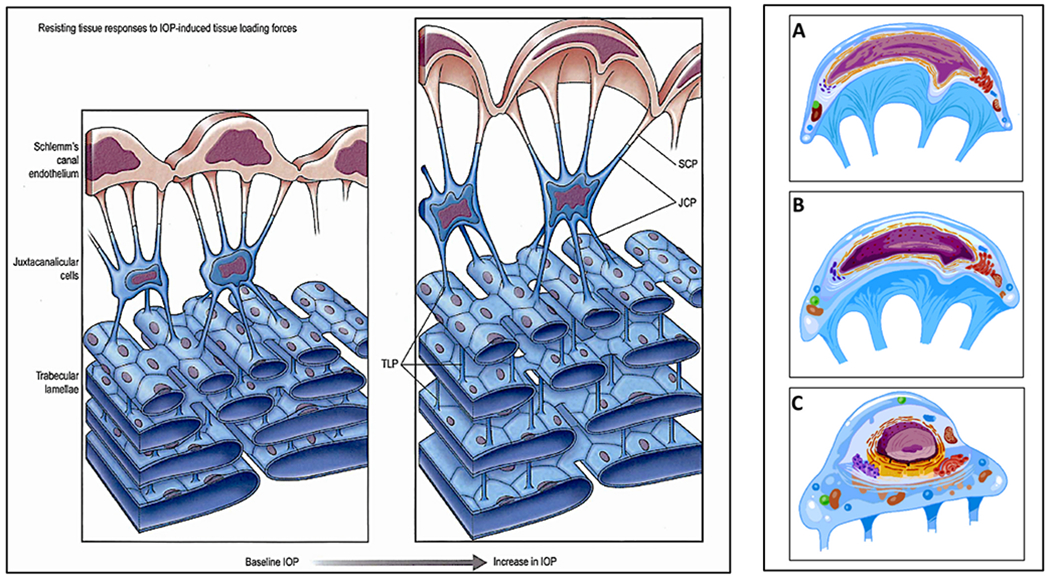
Tethering enables sensory signaling through cell deformation. Cellular connections link the structural elements of the trabecular meshwork (TM). The cytoplasmic process tethering function permits Schlemm’s canal endothelial cell deformation. **Left Panel** Schlemm’s canal endothelial cells (SCE) have processes (SCP) that project into the juxtacanalicular space and attach to juxtacanalicular cell processes (JCP). JCP attach to trabecular lamellae endothelial cell processes (TIP), linking SCE to the trabecular lamellae. Trabecular lamellae endothelial cell cytoplasmic processes connect to adjacent trabecular lamellae cell processes. SC endothelial cell bodies, nuclei, and cytoplasmic processes undergo progressive deformation in response to progressive lOP-induced loading forces. The tethered trabecular lamellae limit SC inner wall endothelium distention by countering lOP-induced SCE loading forces. Spaces between the resisting trabecular tissues progressively increase as lOP increases. At physiologic pressures (baseline lOP), tensional integration is present throughout the trabecular tissues. IOP loading forces presented to SCE distribute throughout the TM lamellae as a result of the tethering cytoplasmic processes. The tensioned network allows finely graded responses to transient increases in lOP. Such force-dependent mechanotransduction mechanisms are like those elsewhere in the vasculature. **Right Panel** Detail of SC inner wall endothelium shown in panel one. The images in **(A)**, **(B)**, **(C)** depict alterations in cell surface membranes, organelles, nuclear envelope, nuclear intermediate filaments, and chromatin resulting from physiologic changes in IOP. The prestressed, tensionally-integrated chromatin permits instantaneous sensing of IOP changes at the genomic level. Pressure-dependent deformation of the cytoplasm, nucleus, and chromatin thus provides mechanotransduction signals to restore homeostatic setpoints (Section 5). The left panel is from “Aqueous Outflow Overview, Diagnosis and Therapy of the Glaucomas. Mosby, St. Louis, 22–46, 2009”. Right panel from Johnstone Glaucoma Lab, University of Washington.

Nuclear intermediate filaments connect the nuclear envelope’s internal surface, the nuclear lamina, to the nuclear chromatin (Maniotis et al., 1997). The linkage causes the physical rearrangement of the nuclear chromatin, which it must do when the nuclear envelope deforms (Dahl et al., 2008), as illustrated by SC endothelium configuration changes (Fig. 25). The connections provide a mechanism to govern genomic regulatory machinery (Sims et al., 1992).

The nuclear chromatin experiences continuous prestress, providing a mechanism for instantaneous responses to force-induced changes in cellular configuration. Upon experiencing new deforming forces on the nuclear membrane, the nuclear scaffold repositions the chromatin, thereby affecting nuclear prestress resulting in gene activation within milliseconds. Thus, pressure changes provide an instantaneous contemporaneous hard-wired signal to the cytoplasm and nucleus of cells throughout the aqueous outflow system. No iterative signaling mechanism is required. In contrast, chemical signaling caused by motor-based translocation either along cytoskeletal filaments or by diffusion of activated regulatory factors takes a few seconds (Hu et al., 2005; Mazumder et al., 2008; Mazumder and Shivashankar, 2010; Snedeker, 2014).

**Fig. 26.**
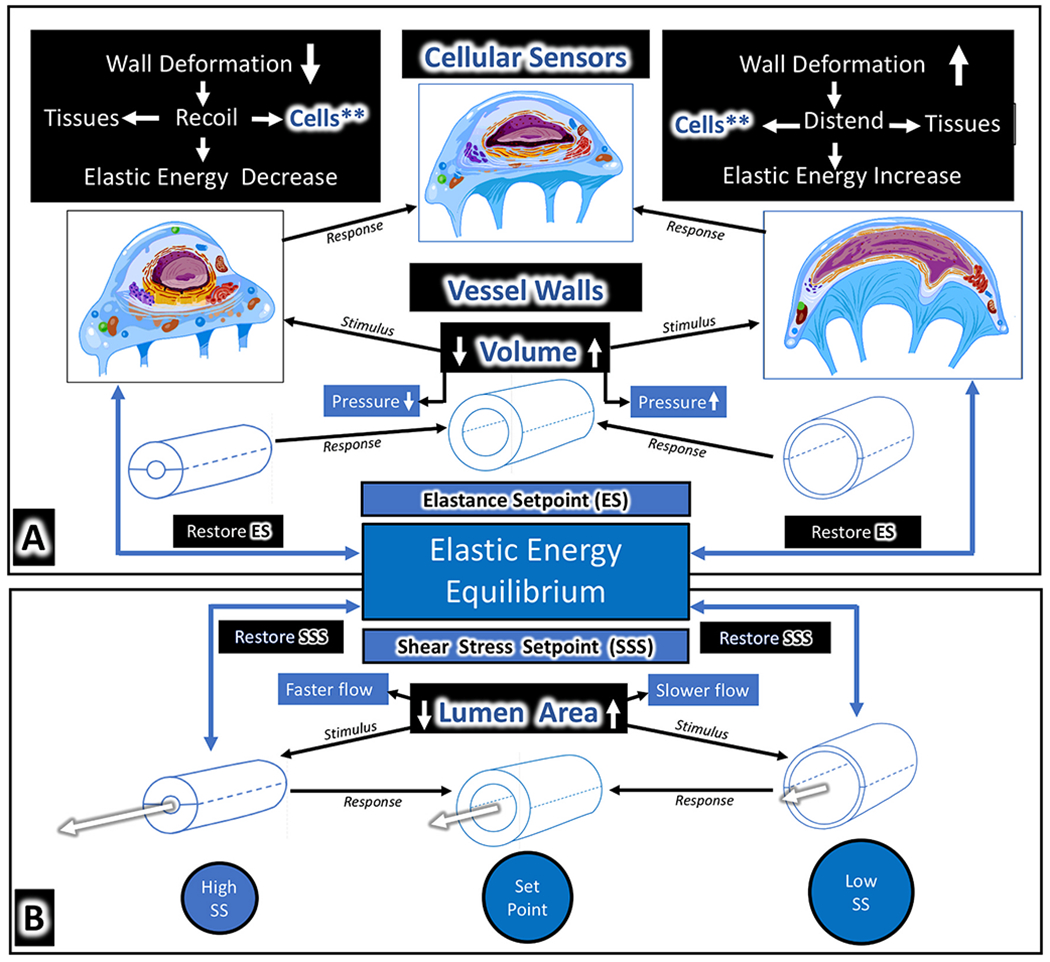
Elastic energy equilibrium: Focal point for vascular homeostasis. During differentiation, each cell type establishes unique, evolutionally optimized cellular stresses that determine internal structure and responses to external stimuli. The stresses establish and define an optimized elastic energy setpoint or equilibrium (Section 5.). The elastic energy setpoint provides a governing framework for the way the twin sensory stimuli of cellular deformation and shear stress interact, thus coordinating mechanotransduction events that maintain homeostasis. **(A)** Vascular endothelial cells sense changes in their lumen volume by force-induced deformation of their shape; **(B)** the same cells sense flow changes by monitoring shear stress on their walls. The aqueous outflow system behavior illustrates how cell deformation and shear stress provide feedback loops in the vasculature that maintain volume and flow homeostasis. Increased pressure induces instantaneous increases in cellular deformation that act as a sensory signal. Cellular deformation or strain causes the cell membrane, cytoskeletal elements, organelles, nuclear membrane, nuclear intermediate filaments, and attached chromatin to all alter their configuration. Cell and tissue constituents respond to the signal by adjusting the tissue and cellular elastance. The altered elastance restores the elastic energy equilibrium that ensures appropriate tissue distension and recoil. Shear stress detects flow. With a constant volume flowing through a vessel, narrowing of the lumen increases flow, resulting in higher shear stress; the increase initiates responses in cells, contiguous extracellular matrix, and muscle in the vessel walls that lead to enlargement of the lumen. Lumen enlargement restores the lumen dimensions and linked shear stress to an equilibrium. Volume changes that induce cell wall deformation and shear responses work in unison to achieve the same homeostatic cell and vessel wall endpoint. From Johnstone Glaucoma Lab, University of Washington, Adapted from Johnstone M, The aqueous outflow system as a mechanical pump: J Glaucoma 13, 421–438.

OCT studies demonstrate that changes in the entire TM-SC endothelium tethered complex’s configuration occur in milliseconds (Li et al., 2012; Hariri et al., 2014; Xin et al., 2016, 2018). Profound deformation of both the cytoplasm and nucleus of endothelial cells of the aqueous outflow system occurs in response to such pressure change (Figs. 23 and 24) (Johnstone and Grant, 1973a; Grierson and Lee, 1975a; Johnstone, 1979). Since cellular systems elsewhere provide instant, contemporaneous, tissue-wide recognition and response to cellular deformation, we may reasonably expect the same responses in the aqueous outflow system, which may be an ideal model for such studies (Fig. 26).

#### 5.2.4. Mechanostat setpoints, boolean networks, boolean attractors, and IOP

Tissue-wide coordination of motion-dependent responses achieves the complex task of maintaining orientation, geometry, and composition in three-dimensional space (Ingber, 2003b). In the outflow system, cellular-based connections, tensional integration, and prestress set the stage for using regulatory mechanisms like those in other tissues (Ingber, 1993; Wang et al., 1993). Cells differentiate to establish an intrinsic cellular tension, a mechanostat setpoint (Snedeker, 2014). However, the loss of homeostatic strain in tendon-like tissue such as the TM lamellae alters the mechanostat setpoint (Arnoczky et al., 2008). For example, age-related changes in ciliary muscle geometry and contractile properties can alter the stresses experienced by the TM lamellae (Lütjen-Drecoll et al., 1988; Strenk et al., 2010).

Boolean networks are widely used models for the description of gene regulatory networks (Kauffman, 1969b). Evolutionary optimized genomic relationships govern setpoints (Kauffman, 1993). Boolean attractors are basins of attraction that converge to a self-stabilizing state space representing an optimized condition (Samuelsson and Troein, 2003; Hopfensitz et al., 2013). A hierarchy of attractors starts with those that maintain the basal vegetative functions of the cells that connect to higher-dimension trajectories responding to the environment, eventually converging on an optimized final state directing the overall behavior of the cell (Huang et al., 2005).

In the outflow system, a combination of wall and shear stress signals can serve as high-dimensional attractor states (Fig. 26). Elastic energy is a form of potential energy representing the ability to induce motion (Fung, 1993). All cells maintain internal stresses that together define the cell’s elastic energy equilibrium. The stresses together define an intrinsic, massively parallel, massively nested information and communication network within each cell (Ingber, 1993, 2003a; Wang et al., 1993; Maniotis et al., 1997); such internal stresses are apparent in SC endothelial cells (Fig. 26) (Johnstone, 1979).

Cells also experience external stresses involving tension resulting from interaction with their substrate or other cells (Fung and Liu, 1993; Fung, 1993). External stresses are apparent in cytoplasmic and nuclear deformation at cytoplasmic processes origins of SC endothelial and juxtacanalicular cells (Figs. 23 and 24) (Johnstone, 1979). When external stresses cause the elastic energy state to change in a tensionally integrated cellular network, every cell’s prestressed genome instantaneously updates (Kauffman, 1993; Maniotis et al., 1997; Pomerance et al., 2009). Genomic mechanisms specify cellular elaboration of ECM material that permits outside-in signaling between cell adhesions molecules and cytoskeletal networks (Wang et al., 1993; Geiger et al., 2001; Ingber, 2002, 2003b). The ECM interaction with the cytoskeleton feeds back to the nuclear membrane and adjusts chromatin prestress and genomic regulation, providing an iterative mechanism to maintain the tensional integration of cellular and ECM components.

The elastic energy state space can serve as a Boolean attractor organizing high-level control of vascular behavior. In contrast to linear electrical and chemical means of communication provided by neural connections, mechanical communication of stress-related information is instantaneous, massively parallel, continuously updating, and specifies a tissue-wide information state. Maintenance of IOP in a narrow range may provide an example of how a genomically controlled elastic energy state acts as an organizing framework for homeostasis. Changes in elastic energy are measurable by assessing elastance curves, which high-resolution OCT can now generate in the aqueous outflow system (Xin et al., 2016).

## 6. Future directions and summary

The study of the integrated machinery of the aqueous outflow pump requires a multidisciplinary approach assembling expertise in tissue morphology, physiology, systems and cell biology, pharmacology, as well as tissue and cellular biomechanics. Signaling pathways linking sensory and motor behavior are essential to a deeper understanding of regulation. The conceptual model we propose places the aqueous outflow pump within the systemic vascular framework, where it is subject to known considerations of cardiovascular and lymphatic physiology.

How does the aqueous outflow pump malfunction in glaucoma? New techniques that can identify and monitor abnormalities of aqueous outflow pump motion in glaucoma can be expected to yield worthwhile insights. Identification of previously unknown IOP control mechanisms and new therapeutic targets offers the possibility of new medications and laser interventions. Surgical innovations can shift from removing or bypassing the TM to restoring pump function without damaging the eye’s natural machinery for regulating aqueous outflow.

The ciliary muscle is a crucial regulator of resistance to aqueous outflow. Studies in eyes retaining intact TM-ciliary muscle relationships may reveal how medications affect synchronous behavior beyond what is known from studying the tissues in isolation. In vivo observations in human subjects in the context of a functioning cardiovascular system in healthy and glaucomatous eyes will be valuable next steps. Such studies are likely to yield insights into physiology, pathology, and medication effects that may be missed or misinterpreted by studying the tissues in isolation.

Nocturnal IOP elevation may result from reduced stroke volume due to reduced nighttime blood pressure and the absence of transients such as blinking and eye movements. Episcleral venous pressure, as well as ciliary body and choroidal volume differences, may affect the pulse amplitude and effective stroke volume in the supine position. PhS-OCT studies may be useful to study differences in pulsatile motion in the supine and upright positions.

A world of anatomy, physiology, and biomechanical properties is unveiled by techniques that dilate SC revealing the SC inlet and outlet valves and their synchronous motion. SEM following viscoelastic dilation of the canal provides unparalleled views of the aqueous outflow pump apparatus, allowing direct observation of the effects of surgical manipulations and medications. SC cannulation with experimentally controlled changes in SC pressure can be done while simultaneously imaging with PhS-OCT. The approach permits real-time investigation of the aqueous drainage system’s synchronous motion under the influence of medications or following surgical manipulations of the TM and ciliary muscle.

We suspect that the septa overlying the CDSP subserve a valve-like function. Their position is controlled by a balance between SC pressure gradients and their attachments to the TM. When pressure gradients favor compression of the CDSP lumen by the septa, aqueous cannot pass through the CC to the CDSP or leave the CDSP via the more distal radial channels. However, their role warrants further study. The role of the septa and CDSP can be elucidated by microscopy in fresh tissue, SEM after SC dilation by viscoelastic, and microvascular casting with clarification. Real-time studies of the distal system can be done in the laboratory with high-resolution SD and in the clinic with PhS-OCT.

PhS-OCT captures the TM lamellae’s motion, a biomechanical property we propose is responsible for maintaining IOP homeostasis. Cross-sectional clinical studies are needed to determine how effectively PhS-OCT can differentiate normal from glaucomatous TM motion. Longitudinal studies are also needed to see how effectively OCT can track progressive deterioration of motion that may indicate a need for therapy escalation. The ability of PhS-OCT to assess medication effectiveness warrants study, especially since several outflow medications restore pulsatile flow within an hour. The rapid pharmacologic response suggests that same-day test-retest results with PhS-OCT may identify medication-induced TM motion changes indicating a meaningful pharmacologic response.

Cataract surgery lowers IOP while altering TM-ciliary body geometric relationships and vector forces, as revealed by high-resolution MRI. This effect on the ciliary muscle is like that of pilocarpine, a drug that alters ciliary muscle geometric relationships and tension; at the same time, the drug restores pulsatile flow and lowers IOP within the duration of its action. These observations offer clues into ways to stent and modulate tension on the capsular bag/zonular system to increase ciliary muscle tension and improve outflow. A novel surgical approach could take the form of spring-loaded split ring capsular implants designed to adhere to the peripheral capsular bag. Once the rings were adherent, heat shrinkage of the spring-loaded region with a laser could adjust zonular tension and vector forces on the ciliary muscle. Scleral expansion approaches and new surgical techniques to tension the ciliary muscle are also possible.

Micropulse laser cyclophotocoagulation can alter TM-ciliary muscle tension. Video of Micropulse Effects (Vid8) 1-s2.0-S1350946220300896-mmc8.mp4 It would be a welcome addition to the therapeutic armamentarium if the transcleral laser procedure could achieve modest but persistent tissue shrinkage without pilocarpine-like side effects. Systematic clinical and laboratory studies are needed to optimize delivery parameters involving the general approach (e.g., sweeping vs. static applications) and device design (e.g., probe tip focusing properties, duration, wavelength, duty cycle, and the relationship between power and duration). Controlled laboratory studies may be valuable for establishing useful clinical parameters.

In summary, this article reviews evidence that the aqueous outflow system functions as a pump-conduit system to regulate aqueous outflow by mechanisms like those in the cardiovascular and lymphatic systems. Clinical evidence includes direct observation of pulsatile flow into SC, collector channels, and aqueous veins. Laboratory evidence includes pressure-dependent TM motion that changes the dimensions of SC, and direct conduits from the TM into SC that functionally behave as SC inlet valves. Hinged flaps or leaflets at CC entrances connected to the pressure-responsive TM can act as outlet valves and respond to IOP changes.

In the proposed pump model, a hierarchy of structural elements acts synchronously, providing an exquisite mechanism to control IOP. This model is different from the proposal of regulation of flow by passage through a filtering system. The concept of an aqueous outflow pump is not new, however. Instead, aspects of the model have been in the scientific literature for decades. This perspective aims to revisit the idea in the light of more recent data that extends the model’s framework. We trust that our proposed general construct will provide fresh perspectives and new realms of exploration pertinent to our shared quest to understand, diagnose, and treat glaucoma.

## Supplementary Material

1

2

3

4

5

6

7

8

## Abbreviations in text and legends

§: Section
(xx)abs: Reference Is Abstract
AC: Anterior Chamber
AVP: Aqueous Vein Pressure
CAS: Cylindrical Attachment Structures (a)
CB: Ciliary Body
CC: Collector Channel
CCE: Collector Channel Entrance (b)
CCO: Collector Channel Ostia (b)
CD31: Vascular Endothelial Cell Label
CDSP: Circumferentially Oriented Deep Scleral Plexus
CLP: Conduit-like Pathway (a)
DAPI: DNA label
ECM: Extracellular Matrix
ESV: Episcleral Vein
EVP: Episcleral Venous Pressure
FEM: Finite Element Method
HCF: Hinged Collagen Flap (c)
IOP: Intraocular Pressure
JCS: Juxtacanalicular Space (d)
JCC: Juxtacanalicular Cell (e)
JCP: Juxtacanalicular Cell Processes
MIGS: Minimally Invasive Glaucoma Surgery
OCT: Optical Coherence Tomography
PhS-OCT: Phase-based OCT
SD-OCT: Spectral Domain OCT
SCE: Schlemm’s Canal Endothelium
SEM: Scanning Electron Microscopy
SC: Schlemm’s Canal
SCP: SC Pressure
SEC: Subendothelial Cell (e)
SES: Subendothelial Space (d)
SIV: Schlemm’s Canal Inlet Valve (a)
SOV: Schlemm’s Canal Outlet Valve (c)
SS: Scleral Spur
TEM: Transmission Electron Microscopy
TLP: TM Lamellae Cytoplasmic Processes
TM: Trabecular Meshwork

(a), (b), (c), (d), (e) Synonyms used in different publications during evolution of concepts.

## References

[R1] Acott TS, Vranka JA, Keller KE, Raghunathan V, Kelley MJ, 2020. Normal and glaucomatous outflow regulation. Prog. Retin. Eye Res 100897. 10.1016/j.preteyeres.2020.100897 [Epub ahead of print] Review. NIHMSID:NIHMS1627339. 32795516 PMC7876168

[R2] Alberts B, 2002. The cytoskeleton. In: Alberts B, Johnson A, Lewis J, Raff M, Roberts K, Walter P (Eds.), Molecular Biology of the Cell. Garland Science, New York.

[R3] Alenghat FJ, Ingber DE, 2002. Mechanotransduction: all signals point to cytoskeleton, matrix, and integrins. Sci. STKE, 2002, pe6. 11842240 10.1126/stke.2002.119.pe6

[R4] Allingham RR, Damji KF, Freedman SF, Moroi SE, Rhee DJ, Shields MB, 2012. Shields Textbook of Glaucoma. Lippincott Williams & Wilkins.

[R5] Arnoczky SP, Lavagnino M, Egerbacher M, Caballero O, Gardner K, Shender MA, 2008. Loss of homeostatic strain alters mechanostat “set point” of tendon cells in vitro. Clin. Orthop. Relat. Res 466, 1583–1591. 18459031 10.1007/s11999-008-0264-x PMC2505257

[R6] Ascher KW, 1949. Aqueous veins and their significance for pathogenesis of glaucoma. Arch. Ophthalmol 42, 66. 10.1001/archopht.1949.00900050069006 18146430

[R7] Ascher KW, 1961. The Aqueous Veins: Biomicroscopic Study of the Aqueous Humor Elimination. Thomas Charles C., Springfield.

[R8] Ascher KW, 1942a. Glaucoma and the aqueous veins. Am. J. Ophthalmol 25, 1309–1315.

[R9] Ascher KW, 1942b. Physiologic importance of the visible elimination of intraocular fluid. Am. J. Ophthalmol 25, 1174–1209. 10.1016/j.ajo.2018.05.025 30055813

[R10] Ascher KW, 1944. Backflow phenomena in aqueous veins. Am. J. Ophthalmol 27, 1074.

[R11] Ascher KW, Spurgeon WM, 1949b. Compression tests on aqueous veins of glaucomatous eyes; application of hydrodynamic principles to the problem of intraocular fluid elimination. Am. J. Ophthalmol 32, 239. 18146427

[R12] Aspelund A, Tammela T, Antila S, Nurmi H, Leppanen VM, Zarkada G, Stanczuk L, Francois M, Makinen T, Saharinen P, Immonen I, Alitalo K, 2014. The schlemm’s canal is a vegf-c/vegfr-3-responsive lymphatic-like vessel. J. Clin. Invest 124, 3975–3986. 25061878 10.1172/JCI75395 PMC4153703

[R13] Bentley MD, Hann CR, Fautsch MP, 2016. Anatomical variation of human collector channel orifices. Invest. Ophthalmol. Vis. Sci 57, 1153–1159. 26975026 10.1167/iovs.15-17753 PMC4794087

[R14] Bill A, 1970. Scanning electron microscopy studies of the canal of schlemm. Exp. Eye Res 10, 214–218. 4991873 10.1016/s0014-4835(70)80030-6

[R15] Bill A, Svedbergh B, 1972. Scanning electron microscopic studies of the trabecular meshwork and the canal of schlemm–an attempt to localize the main resistance to outflow of aqueous humor in man. Acta Ophthalmol (Copenh) 50, 295–320. 4678226 10.1111/j.1755-3768.1972.tb05954.x

[R16] Bloom G, Friberg U, 1956. Shrinkage during fixation and embedding of histological specimens. Acta Morphol. Neerl-Scand 1, 12. 13424175

[R17] Brown KA, Ditchey, 1988. Human right ventricular end-systolic pressure-volume relation defined by maximal elastance. Circulation 78, 81–91. 3383413 10.1161/01.cir.78.1.81

[R18] Cambiaggi A, 1958. Effeto della jaluronidasi sulla pressone intraocular e sull’asetto della vene dell’accqueo. Boll. Soc. di Biologia Sperimentale 34, 1–7. 13607797

[R19] Carreon T, Van Der Merwe E, Fellman RL, Johnstone M, Bhattacharya SK, 2017. Aqueous outflow - a continuum from trabecular meshwork to episcleral veins. Prog. Retin. Eye Res 57, 108–133. 28028002 10.1016/j.preteyeres.2016.12.004 PMC5350024

[R20] Chi HH, Katzin HM, Teng CC, 1957. Primary degeneration in the vicinity of the chamber angle; as an etiologic factor in wide-angle glaucoma. Ii. Am. J. Ophthalmol 43, 193–203. 13394666 10.1016/0002-9394(57)92910-0

[R21] Coleman DJ, Trokel S, 1969. Direct-recorded intraocular pressure variations in a human subject. Arch. Ophthalmol 82, 637–640. 5357713 10.1001/archopht.1969.00990020633011

[R22] Coupland SE, Penfold PL, Billson FA, 1993. Histochemical survey of the anterior segment of the normal human foetal and adult eye. Graefes Arch. Clin. Exp. Ophthalmol 231, 533–540. 8224958 10.1007/BF00921119

[R23] Curtiss K, Hann C, Possin D, Wang B, Martin E, Slabaugh M, Johnstone M, 2011. New insights into schlemm’s canal (sc) structural relationships using multiple imaging modalities and 3d reconstructions. Invest. Ophthalmol. Vis. Sci 52, 4667.

[R24] Dahl KN, Ribeiro AJ, Lammerding J, 2008. Nuclear shape, mechanics, and mechanotransduction. Circ. Res 102, 1307–1318. 18535268 10.1161/CIRCRESAHA.108.173989 PMC2717705

[R25] De Kater AW, Shahsafaei A, Epstein DL, 1992. Localization of smooth muscle and nonmuscle actin isoforms in the human aqueous outflow pathway. Invest. Ophthalmol. Vis. Sci 33, 424–429. 1740375

[R26] De Vries S, 1947. De Zichtbare Afvoer Van Het Kamerwater. Drukkerij Kinsbergen, Amsterdam.

[R27] Desai BV, Harmon RM, Green KJ, 2009. Desmosomes at a glance. J. Cell Sci 122, 4401–4407. 19955337 10.1242/jcs.037457 PMC2787455

[R28] Dvorak-Theobald G, Kirk HQ, 1956. Aqueous pathways in some cases of glaucoma. Am. J. Ophthalmol 41, 11–21. 13275540

[R29] Ellingsen BA, Grant WM, 1971a. Influence of intraocular pressure and trabeculotomy on aqueous outflow in enucleated monkey eyes. Invest. Ophthalmol 10, 705–709. 4999351

[R30] Ellingsen BA, Grant WM, 1971b. The relationship of pressure and aqueous outflow in enucleated human eyes. Invest. Ophthalmol 10, 430–437. 5578207

[R31] Ellingsen BA, Grant WM, 1972. Trabeculotomy and sinusotomy in enucleated human eyes. Invest. Ophthalmol 11, 21–28. 5006959

[R32] Ethier CR, 2002. The inner wall of schlemm’s canal. Exp. Eye Res 74, 161–172. 11950226 10.1006/exer.2002.1144

[R33] Ethier CR, Read AT, Chan D, 2004. Biomechanics of schlemm’s canal endothelial cells: influence on f-actin architecture. Biophys. J 87, 2828–2837. 15454474 10.1529/biophysj.103.038133 PMC1304701

[R34] Fellman RL, Grover DS, 2014. Episcleral Venous Fluid Wave: Intraoperative Evidence for Patency of the Conventional Outflow System, pp. 347–350. 10.1097/IJG.0b013e31827a06d8 23282859

[R35] Filla MS, Dimeo KD, Tong T, Peters DM, 2017. Disruption of fibronectin matrix affects type iv collagen, fibrillin and laminin deposition into extracellular matrix of human trabecular meshwork (htm) cells. Exp. Eye Res 165, 7–19. 28860021 10.1016/j.exer.2017.08.017 PMC5705399

[R36] Flocks M, 1957. The anatomy of the trabecular meshwork as seen in tangential section. Arch. Ophthalmol 56, 708–718. 10.1001/archopht.1956.00930040716010 13361636

[R37] Flügel-Koch C, Neuhuber WL, Kaufman PL, Lütjen-Drecoll E, 2009. Morphologic indication for proprioception in the human ciliary muscle. Invest. Ophthalmol. Vis. Sci 50, 5529–5536. 19578020 10.1167/iovs.09-3783 PMC2810743

[R38] Flügel C, Bárány EH, Lütjen-Drecoll E, 1990a. Histochemical differences within the ciliary muscle and its function in accommodation. Exp. Eye Res 50, 219–226. 2138092 10.1016/0014-4835(90)90234-l

[R39] Flügel C, Lütjen-Drecoll E, Bárány E, 1990b. [structural differences in the structure of the ciliary muscles in eyes of primates. A histochemical and morphological study]. Fortschr. Ophthalmol 87, 384–387. 2210568

[R40] Freddo TF, Townes-Anderson E, Raviola G, 1980. Rod-shaped bodies and crystalloid inclusions in ocular vascular endothelia of adult and developing macaca mulatta. Anat. Embryol 158, 121–131. 10.1007/BF00315900 6243890

[R41] Fuchshofer R, Welge-Lüssen U, Lütjen-Drecoll E, Birke M, 2006. Biochemical and morphological analysis of basement membrane component expression in corneoscleral and cribriform human trabecular meshwork cells. Invest. Ophthalmol. Vis. Sci 47, 794–801. 16505009 10.1167/iovs.05-0292

[R42] Fung YC, 1993. Biomechanics Mechanical Structure of Living Cells. Springer, New York.

[R43] Fung YC, Liu SQ, 1993. Elementary mechanics of the endothelium of blood vessels. J. Biomech. Eng 115, 1–12. 8445886 10.1115/1.2895465

[R44] Fung YC, 1996. Biomechanics: Circulation. Springer-Verlag, New York.

[R45] Gabelt BT, Kaufman PL, 2005. Changes in aqueous humor dynamics with age and glaucoma. Prog. Retin. Eye Res 24, 612–637. 15919228 10.1016/j.preteyeres.2004.10.003

[R46] Gao K, Song S, Johnstone MA, Wang RK, Wen JC, 2019. Trabecular meshwork motion in normal compared with glaucoma eyes. Invest. Ophthalmol. Vis. Sci 60, 4824.

[R47] Garron L, Feeney M.l., Hogn MJ, McEwen w.k., 1958. Electron microscopic studies of the human eye. Am. J. Ophthalmol 46, 27–35. 10.1016/0002-9394(58)90031-x 13545326

[R48] Gattinoni L, Chiumello D, Carlesso E, Valenza F, 2004. Bench-to-bedside review: chest wall elastance in acute lung injury/acute respiratory distress syndrome patients. Crit. Care 8, 350. 15469597 10.1186/cc2854 PMC1065004

[R49] Geiger B, Bershadsky A, Pankov R, Yamada KM, 2001. Transmembrane crosstalk between the extracellular matrix–cytoskeleton crosstalk. Nat. Rev. Mol. Cell Biol 2, 793–805. 11715046 10.1038/35099066

[R50] Goldmann H, 1946a. Abfluss des kammerwassers beim menschen. Ophthalmologica 111, 146–152. 20275796 10.1159/000300317

[R51] Goldmann H, 1946b. Weitere mitteilung liber den abfluss des kammerwassers beim menschen. Ophthalmologica 112, 344–346. 20293720 10.1159/000300402

[R52] Goldmann H, 1948. Uber abflussdruck und glasstab-phanomen. Pathogenese des einfachen glaukoms. Ophthalmologica 116, 193. 10.1159/000300593 18100962

[R53] Gonzalez JM Jr., Hsu HY, Tan JCH, 2014. Observing live actin in the human trabecular meshwork. Clin. Exp. Ophthalmol 42, 502–504. 24304516 10.1111/ceo.12255 PMC4004729

[R54] Gonzalez JM, Heur M, Tan JC, 2012. Two-photon immunofluorescence characterization of the trabecular meshwork in situ. Invest. Ophthalmol. Vis. Sci 53, 3395–3404. 22531697 10.1167/iovs.11-8570 PMC3374625

[R55] Gonzalez JM, Hamm-Alvarez S, Tan JCH, 2013. Analyzing live cellularity in the human trabecular meshwork. Invest. Ophthalmol. Vis. Sci 54, 1039–1047. 23249706 10.1167/iovs.12-10479 PMC3565993

[R56] Gonzalez JM, Ko MK, Hong Y-K, Weigert R, Tan JCH, 2017. Deep tissue analysis of distal aqueous drainage structures and contractile features. Sci. Rep 7, 17071. 29213129 10.1038/s41598-017-16897-y PMC5719038

[R57] Gonzalez JM, Ko MK, Pouw A, Tan JCH, 2016. Tissue-based multiphoton analysis of actomyosin and structural responses in human trabecular meshwork. Sci. Rep 6, 21315. 26883567 10.1038/srep21315 PMC4756353

[R58] Grant WM, 1958. Further studies on facility of flow through the trabecular meshwork. Arch. Ophthalmol 60, 523–533. 10.1001/archopht.1958.00940080541001 13582305

[R59] Grant WM, 1963. Experimental aqueous perfusion in enucleated human eyes. Arch. Ophthalmol 69, 783–801. 13949877 10.1001/archopht.1963.00960040789022

[R60] Grierson I, Lee WR, 1974a. Changes in the monkey outflow apparatus at graded levels of intraocular pressure: a qualitative analysis by light microscopy and scanning electron microscopy. Exp. Eye Res 19, 21–33. 4412389 10.1016/0014-4835(74)90068-2

[R61] Grierson I, Lee WR, 1974b. Junctions between the cells of the trabecular meshwork. Albrecht Von Graefes Arch. Klin. Exp. Ophthalmol 192, 89–104. 4140699 10.1007/BF00410696

[R62] Grierson I, Lee WR, 1975a. The fine structure of the trabecular meshwork at graded levels of intraocular pressure. (1) pressure effects within the near-physiological range (8-30 mmhg). Exp. Eye Res 20, 505–521. 1149832 10.1016/0014-4835(75)90218-3

[R63] Grierson I, Lee WR, 1975b. The fine structure of the trabecular meshwork at graded levels of intraocular pressure. (2) pressures outside the physiological range (0 and 50 mmhg). Exp. Eye Res 20, 523–530. 168092 10.1016/0014-4835(75)90219-5

[R64] Grierson I, Lee WR, Abraham S, Howes RC, 1978. Associations between the cells of the walls of schlemm’s canal. Albrecht Von Graefes Arch. Klin. Exp. Ophthalmol 208, 33–47. 103456 10.1007/BF00406980

[R65] Grieshaber MC, Peckar C, Pienaar A, Koerber N, Stegmann R, 2015. Long-term results of up to 12 years of over 700 cases of viscocanalostomy for open-angle glaucoma. Acta Ophthalmol 93, 362–367. 25270165 10.1111/aos.12513

[R66] Grieshaber MC, Pienaar A, Olivier J, Stegmann R, 2010. Canaloplasty for primary open-angle glaucoma: long-term outcome. Br. J. Ophthalmol 94, 1478–1482. 20962352 10.1136/bjo.2009.163170

[R67] Grover DS, Fellman RL, 2016. Gonioscopy-assisted transluminal trabeculotomy (gatt): thermal suture modification with a dye-stained rounded tip. J. Glaucoma 25, 501–504. 26398579 10.1097/IJG.0000000000000325

[R68] Hamanaka T, Bill A, Ichinohasama R, Ishida T, 1992. Aspects of the development of schlemm’s canal. Exp. Eye Res 55, 479–488. 1426078 10.1016/0014-4835(92)90121-8

[R69] Hamanaka T, Thornell LE, Bill A, 1997. Cytoskeleton and tissue origin in the anterior cynomolgus monkey eye. Jpn. J. Ophthalmol 41, 138–149. 9243309 10.1016/s0021-5155(97)00031-2

[R70] Hann CR, Fautsch MP, 2009. Preferential fluid flow in the human trabecular meshwork near collector channels. Invest. Ophthalmol. Vis. Sci 50, 1692–1697. 19060275 10.1167/iovs.08-2375 PMC2681099

[R71] Hann CR, Fautsch MP, 2011. The elastin fiber system between and adjacent to collector channels in the human juxtacanalicular tissue. Invest. Ophthalmol. Vis. Sci 52, 45–50. 20720231 10.1167/iovs.10-5620 PMC3053290

[R72] Hann CR, Bentley MD, Vercnocke A, Ritman EL, Fautsch MP, 2011. Imaging the aqueous humor outflow pathway in human eyes by three-dimensional micro-computed tomography (3d micro-ct). Exp. Eye Res 92, 104–111. 21187085 10.1016/j.exer.2010.12.010 PMC3034776

[R73] Hann CR, Vercnocke AJ, Bentley MD, Jorgensen SM, Fautsch MP, 2014. Anatomic changes in schlemm’s canal and collector channels in normal and primary open-angle glaucoma eyes using low and high perfusion pressures. Invest. Opthalmol. Vis. Sci 55, 5834–5841. 10.1167/iovs.14-14128 PMC4165370 25139736

[R74] Hariri S, Johnstone M, Jiang Y, Padilla S, Zhou Z, Reif R, Wang RK, 2014. Platform to investigate aqueous outflow system structure and pressure-dependent motion using high-resolution spectral domain optical coherence tomography. J. Biomed. Optic 19, 106013 1–10601311. 10.1117/1.JBO.19.10.106013 PMC4210620 25349094

[R75] Harvey W, 1970. Exercitatio Anatomica de Motu Cordis et Sanguinis in Animalibus. Thomas Charles C., Springfield. 1090176

[R76] Heijl A, Bengtsson B, Hyman L, Leske MC, Early MGTG, 2009. Natural history of open-angle glaucoma. Ophthalmology 116, 2271–2276. 19854514 10.1016/j.ophtha.2009.06.042

[R77] Hernandez MR, Gong H, 1996. Extracellular matrix of the trabecular meshwork and optic nerve head. In: Ritch R, Shields MB, Krupin T (Eds.), The Glaucomas, second ed. Mosby, St. Louis, pp. 213–249.

[R78] Hodgson TH, Macdonald RK, 1954. Slitlamp studies on the flow of aqueous humor. Br. J. Ophthalmol 38, 266. 13160319 10.1136/bjo.38.5.266 PMC1324326

[R79] Hoffmann F, Dumitrescu L, 1971. Schlemm’s canal under the scanning electron microscope. Ophthalmic Res 2, 37–45.

[R80] Hogan MJ, Alvarado J, Weddell JE, 1971. Histology of the Human Eye, and Atlas and Textbook. Saunders, Philadelphia.

[R81] Holmberg A, 1959. The fine structure of the inner wall of schlemm’s canal. Arch. Ophtalmol 62, 956.

[R82] Holmberg AS, 1965. Schlemm’s canal and the trabecular meshwork. An electron microscopic study of the normal structure in man and monkey (cercopithecus ethiops). Documentia Ophthalmolgoica 29, 339–373.

[R83] Hopfensitz M, Müssel C, Maucher M, Kestler HA, 2013. Attractors in boolean networks: a tutorial. Comput. Stat 28, 19–36.

[R84] Hu S, Chen J, Butler JP, Wang N, 2005. Prestress mediates force propagation into the nucleus. Biochem. Biophys. Res. Commun 329, 423–428. 15737604 10.1016/j.bbrc.2005.02.026

[R85] Huang AS, Camp A, Xu BY, Penteado RC, Weinreb RN, 2017a. Aqueous angiography: aqueous humor outflow imaging in live human subjects. Ophthalmology 124, 1249–1251. 28461013 10.1016/j.ophtha.2017.03.058 PMC5522757

[R86] Huang AS, Li M, Yang D, Wang H, Wang N, Weinreb RN, 2017b. Aqueous angiography in living nonhuman primates shows segmental, pulsatile, and dynamic angiographic aqueous humor outflow. Ophthalmology 124, 793–803. 28237425 10.1016/j.ophtha.2017.01.030 PMC5484000

[R87] Huang AS, Saraswathy S, Dastiridou A, Begian A, Legaspi H, Mohindroo C, Tan JC, Francis BA, Caprioli J, Hinton DR, Weinreb RN, 2016a. Aqueous angiography with fluorescein and indocyanine green in bovine eyes. Transl Vis Sci Technol 5, 5. 10.1167/tvst.5.6.5 PMC5106193 27847692

[R88] Huang AS, Saraswathy S, Dastiridou A, Begian A, Mohindroo C, Tan JC, Francis BA, Hinton DR, Weinreb RN, 2016b. Aqueous angiography-mediated guidance of trabecular bypass improves angiographic outflow in human enucleated eyes. Invest. Ophthalmol. Vis. Sci 57, 4558–4565. 27588614 10.1167/iovs.16-19644 PMC5017267

[R89] Huang S, Eichler G, Bar-Yam Y, Ingber DE, 2005. Cell fates as high-dimensional attractor states of a complex gene regulatory network. Phys. Rev. Lett 94, 128701. 15903968 10.1103/PhysRevLett.94.128701

[R90] Humphrey JD, 2002. Cardiovascular Solid Mechanics: Cells, Tissues, and Organs, first ed. Springer-Verlag, New York.

[R91] Ingber DE, 1993. Cellular tensegrity: defining new rules of biological design that govern the cytoskeleton. J. Cell Sci 104, 613–627. 8314865 10.1242/jcs.104.3.613

[R92] Ingber DE, 2003a. Tensegrity I. Cell structure and hierarchical systems biology. J. Cell Sci 116, 1157–1173. 12615960 10.1242/jcs.00359

[R93] Ingber DE, 2002. Mechanical signaling and the cellular response to extracellular matrix in angiogenesis and cardiovascular physiology. Circ. Res 91, 877–887. 12433832 10.1161/01.res.0000039537.73816.e5

[R94] Ingber DE, 2003b. Mechanosensation through integrins: cells act locally but think globally. Proc. Natl. Acad. Sci. U. S. A 100, 1472–1474. 12578965 10.1073/pnas.0530201100 PMC149854

[R95] Inomata H, Bill A, Smelser GK, 1972. Aqueous humor pathways through the trabecular meshwork and into schlemm’s canal in the cynomolgus monkey (macaca irus). An electron microscopic study. Am. J. Ophthalmol 73, 760–789. 4623937 10.1016/0002-9394(72)90394-7

[R96] Jamil A, Martin E, Curtiss K, Samuelson T, Chen P, Johnstone M, 2010. Transparent cylindrical structures spanning schlemm’s canal: examination by oblique light, phase contrast, differential interference contrast (nomarski) and fluorescence microscopy. Invest. Ophthalmol. Vis. Sci 52, 3214.

[R97] Johnson M, Chan D, Read AT, Christensen C, Sit A, Ethier CR, 2002. The pore density in the inner wall endothelium of schlemm’s canal of glaucomatous eyes. Invest. Ophthalmol. Vis. Sci 43, 2950–2955. 12202514

[R98] Johnstone M, 2016. Intraocular pressure control through linked trabecular meshwork and collector channel motion. In: Samples JR, Knepper PA (Eds.), Glaucoma Research and Clinical Advances: 2016 to 2018. Kugler Publications, Amsterdam.

[R99] Johnstone M, Curtiss K, Possin D, Huang J, Slabaugh M, 2011a. New imaging techniques to study transparent tubules spanning schlemm’s canal, structures subject to disruption by schlemm’s canal surgery. Amer.Gla.Soc, p. 103

[R100] Johnstone M, Hann C, Fautsch M, Curtiss K, Martin E, Slabaugh M, 2011b. Endothelial-lined Aqueous Conduits Span Schlemm’s Canal (SC) to Attach to Collector Channel Ostia: Identification by 3D Reconstruction of Histologic Sections, p. 47.

[R101] Johnstone M, Jamil A, Martin E, 2010. Aqueous veins and open angle glaucoma. In: Schacknow P, Samples J (Eds.), The Glaucoma Book Springer, New York, pp. 65–78.

[R102] Johnstone M, Martin E, Jamil A, 2011c. Pulsatile flow into the aqueous veins: manifestations in normal and glaucomatous eyes. Exp. Eye Res 92, 318–327. 21440541 10.1016/j.exer.2011.03.011 PMC4535453

[R103] Johnstone M, Martin E, Jamil A, 2007. Latanoprost instillation results in a rapid directly measurable increase in conventional aqueous outflow. Invest. Ophthalmol. Vis. Sci 48S, 76.

[R104] Johnstone M, Martin E, Jiang Y, 2014a. Pulse-dependent trabecular meshwork motion: direct microscope observation and measurement in radial limbal segments of non-human primate eyes. Invest. Ophthalmol. Vis. Sci 55, 2169.

[R105] Johnstone M, Mills R, 2004. The slit lamp, the ophthalmoscope and the genome: clinically visible parallels of mechanotransduction in the aqueous outflow and vascular circulatory systems. Am. Glaucoma Soc 13, 56.

[R106] Johnstone M, Possin D, Curtiss K, Martin E, Slabaugh M, 2011d. New noninvasive techniques to characterize schlemm’s canal endothelial cell (see) topography and relationships: adjunct to scanning electron microscopy (sem). Invest. Ophthalmol. Vis. Sci 52, 4639.21498607

[R107] Johnstone M, Stegmann R, Martin E, Jamil A, 2009. Pulsatile circumferential aqueous flow into schlemm’s canal is synchronous with the cardiac pulse. Invest. Ophthalmol. Vis. Sci 50, 28.

[R108] Johnstone M, 2009. Aqueous humor outflow. In: Stamper R, Lieberman MF, Drake MV (Eds.), Diagnosis and Therapy of the Glaucomas. Mosby, St. Louis, pp. 22–46.

[R109] Johnstone MA, 1974. Pressure-dependent changes in configuration of the endothelial tubules of schlemm’s canal. Am. J. Ophthalmol 78, 630–638. 4415190 10.1016/s0002-9394(14)76301-9

[R110] Johnstone MA, 1979. Pressure-dependent changes in nuclei and the process origins of the endothelial cells lining schlemm’s canal. Invest. Ophthalmol. Vis. Sci 18, 44–51. 103860

[R111] Johnstone MA, 2006. A new model describes an aqueous outflow pump and explores causes of pump failure in glaucoma. In: Grehn H, Stamper R (Eds.), Essentials in Ophthalmology: Glaucoma II. Springer, Heidelberg.

[R112] Johnstone MA, 1984. The morphology of the aqueous outflow channels. In: Drance SM (Ed.), Glaucoma: Applied Pharmacology in Medical Treatment. Grune & Stratton, New York, pp. 87–109.

[R113] Johnstone MA, Grant WM, 1973a. Pressure-dependent changes in structure of the aqueous outflow system in human and monkey eyes. Am. J. Ophthalmol 75, 365–383. 4633234 10.1016/0002-9394(73)91145-8

[R114] Johnstone MA, Tanner D, Chau B, Kopecky K, 1980. Concentration-dependent morphologic effects of cytoehalasin b in the aqueous outflow system. Invest. Ophthalmol. Vis. Sci 19, 835–841. 6771222

[R115] Johnstone MA, 2004. The aqueous outflow system as a mechanical pump: evidence from examination of tissue and aqueous movement in human and non-human primates. J. Glaucoma 13, 421–438. 15354083 10.1097/01.ijg.0000131757.63542.24

[R116] Johnstone MA, 2014. Intraocular pressure regulation: findings of pulse-dependent trabecular meshwork motion lead to unifying concepts of intraocular pressure homeostasis. J. Ocul. Pharmacol. Therapeut 30, 88–93. 10.1089/jop.2013.0224 PMC3991971 24359130

[R117] Johnstone MA, Grant WM, 1973b. Microsurgery of schlemm’s canal and the human aqueous outflow system. Am. J. Ophthalmol 76, 906–917. 4759850 10.1016/0002-9394(73)90080-9

[R118] Johnstone MA, Martin E, Jamil A, 2008. Travoprost instillation results in a rapid directly observable increase in conventional aqueous outflow in normal subjects. Invest. Ophthalmol. Vis. Sci 49S.

[R119] Johnstone MA, Saheb H, Ahmed II, Samuelson TW, Schieber AT, Toris CB, 2014b. Effects of a schlemm canal scaffold on collector channel ostia in human anterior segments. Exp. Eye Res 119, 70–76. 24374259 10.1016/j.exer.2013.12.011

[R120] Johnstone MA, Ethier RC, Acott TS, Vranka J, Padilla SM, Wen K, Xin C, Zhang L, Song S, Wang RK, 2018. Collector channel dynamics: oct capture of real-time pressure-dependent changes in lumen area in ex vivo normal and glaucomatous eyes. Invest. Ophthalmol. Vis. Sci 59, 5907.

[R121] Johnstone MA, Jiang Y, Padilla S, Xin C, Martin E, Wang R, 2016. Aqueous outflow pathways that may be specially organized to sense flow and pressure. Invest. Ophthalmol. Vis. Sci 57, 4704.27607416

[R122] Johnstone MA, Song S, Padilla S, Wen K, Xin C, Wen JC, Martin E, Wang RK, 2019. Microscope real-time video high-resolution oct & histopathology to assess how transcleral micropulse laser affects the sclera, ciliary body muscle, secretory epithelium, suprachoroidal space & aqueous outflow system. Invest. Ophthalmol. Vis. Sci 60, 2825.

[R123] Kauffman S, 1969a. Homeostasis and differentiation in random genetic control networks. Nature 224, 177–178. 5343519 10.1038/224177a0

[R124] Kauffman SA, 1969b. Metabolic stability and epigenesis in randomly constructed genetic nets. J. Theor. Biol 22, 437–467. 5803332 10.1016/0022-5193(69)90015-0

[R125] Kauffman SA, 1993. The Origins of Order: Self-Organization and Selection in Evolution. OUP USA.

[R126] Kaufman PL, 2020. Deconstructing aqueous humor outflow-the last 50 years. Exp. Eye Res 108105. 32590004 10.1016/j.exer.2020.108105 PMC7990028

[R127] Kaufman PL, Barany EH, 1976. Loss of acute pilocarpine effect on outflow facility following surgical disinsertion and retrodisplacement of the ciliary muscle from the scleral spur in the cynomolgus monkey. Invest. Ophthalmol 15, 793–807. 824222

[R128] Khatib TZ, Meyer PAR, Lusthaus J, Manyakin I, Mushtaq Y, Martin KR, 2019. Hemoglobin video imaging provides novel in vivo high-resolution imaging and quantification of human aqueous outflow in patients with glaucoma. Ophthalmol. Glaucoma 2, 327–335. 31788668 10.1016/j.ogla.2019.04.001 PMC6876656

[R129] Kizhatil K, Ryan M, Marchant JK, Henrich S, John SWM, 2014. Schlemm’s canal is a unique vessel with a combination of blood vascular and lymphatic phenotypes that forms by a novel developmental process. PLoS Biol 12, el001912. 10.1371/journal.pbio.1001912 PMC4106723 25051267

[R130] Kleinert H, 1951a. Das durch druck auf das auge erzielte ruckflussphanomen in den kammerwasservenen. Klin. Monatsblatter Augenheilkd. 122, 726.

[R131] Kleinert H, 1951b. Der sichtbare abfluss des kammerwassers in den epibulbaren venen. von Graefes Arch. Ophth 152, 278–299. 14933283

[R132] Kleinert H, 1951c. The compensation maximum: a new glaucoma sign in aqueous veins. Arch. Ophthalmol 46, 618–624. 14868091

[R133] Knowlton FP, Starlings EH, 1912. The influence of variations in temperature and blood-pressure on the performance of the isolated mammalian heart. J. Physiol 44, 206–219. 16993122 10.1113/jphysiol.1912.sp001511 PMC1512817

[R134] Krasnov MM, 1964. [sinusotomy in glaucoma]. Vestn. Oftalmol 77, 37–41. 14176823

[R135] Krasnov MM, 1969. Microsurgery of glaucoma: indications and choice of techniques. Am. J. Ophthalmol 67, 857–864. 5785848 10.1016/0002-9394(69)90079-8

[R136] Krohn J, 1999. Expression of factor viii-related antigen in human aqueous drainage channels. Acta Ophthalmol. Scand 77, 9–12. 10071139 10.1034/j.1600-0420.1999.770102.x

[R137] Kronfeld PC, 1949. Further gonioscopic studies on the canal of schlemm. Arch. Ophthalmol 41, 393–405. 10.1001/archopht.1949.00900040403001 18119656

[R138] Kuchtey J, Chang TC, Panagis L, Kuchtey RW, 2013. Marfan syndrome caused by a novel fbn1 mutation with associated pigmentary glaucoma. Am. J. Med. Genet 161A, 880–883. 23444230 10.1002/ajmg.a.35838 PMC3638319

[R139] Leber T, 1873. Studien über den flüssigkeitswechsel im auge. Albr. v. Gr. Arch. Ophthal 19, 87–106.

[R140] Lee WR, Grierson I, 1974. Relationships between intraocular pressure and the morphology of the outflow apparatus. Trans. Ophthalmol. Soc. U. K 94, 430–449. 4219862

[R141] Lee WR, Grierson I, 1975. Pressure effects on the endothelium of the trabecular wall of schlemm’s canal: a study by scanning electron microscopy. Albrecht Von Graefes Arch. Klin. Exp. Ophthalmol 196, 255–265. 813538 10.1007/BF00410037

[R142] Levick JR, 2000. Cardiovascular Physiology, third ed.

[R143] Levick JR, 2010. Introduction to Cardiovascular Physiology. Hodder Education, a Hachette UK Comp., London.

[R144] Li P, Reif R, Zhi Z, Martin E, Shen TT, Johnstone M, Wang RK, 2012. Phase-sensitive optical coherence tomography characterization of pulse-induced trabecular meshwork displacement in ex vivo nonhuman primate eyes. J. Biomed. Optic 17, 076026. 10.1117/1.JBO.17.7.076026 22894509

[R145] Li P, Shen TT, Johnstone M, Wang RK, 2013. Pulsatile motion of the trabecular meshwork in healthy human subjects quantified by phase-sensitive optical coherence tomography. Biomed. Optic Express 4, 2051–2065. 10.1364/BOE.4.002051 PMC3799665 24156063

[R146] Lütjen-Drecoll E, Eichhorn M, 1988. [morphological principles of the aqueous humor secretory system and its changes induced by antiglaucoma drugs]. Fortschr. Ophthalmol 85, 25–32. 3371812

[R147] Lütjen-Drecoll E, Shimizu T, Rohrbach M, Rohen JW, 1986. Quantitative analysis of ‘plaque material’ between ciliary muscle tips in normal- and glaucomatous eyes. Exp. Eye Res 42, 457–465. 3720864 10.1016/0014-4835(86)90005-9

[R148] Lütjen-Drecoll E, Tamm E, Kaufman PL, 1988. Age changes in rhesus monkey ciliary muscle: light and electron microscopy. Exp. Eye Res 47, 885–899. 3215297 10.1016/0014-4835(88)90070-x

[R149] Lütjen-Drecoll E, Rohen JW, 1996. Morphology of aqueous outflow in normal and glaucomatous eyes. In: Ritch R, Krupin T, Shields MB (Eds.), The Glaucomas. Mosby, New York, pp. 89–123.

[R150] Majno G, Shea SM, Leventhal M, 1969. Endothelial contraction induced by histamine-type mediators, an electron microscopic study. J. Cell Biol 42, 647–672. 5801425 10.1083/jcb.42.3.647 PMC2107712

[R151] Maniotis AJ, Chen CS, Ingber DE, 1997. Demonstration of mechanical connections between integrins, cytoskeletal filaments, and nucleoplasm that stabilize nuclear structure. Proc. Natl. Acad. Sci. Unit. States Am 94, 849–854. 10.1073/pnas.94.3.849 PMC19602 9023345

[R152] Mansberger SL, Gordon MO, Jampel H, Bhorade A, Brandt JD, Wilson B, Kass MA, Ocular HTSG, 2012. Reduction in intraocular pressure after cataract extraction: the ocular hypertension treatment study. Ophthalmology 119, 1826–1831. 22608478 10.1016/j.ophtha.2012.02.050 PMC3426647

[R153] Martin E, Jiang Y, Johnstone M, 2014. Schlemm’s canal (SC) and distal aqueous outflow pathways: new scanning em (SEM) preparation technique permits identifying unique structural relationships. Invest Ophthalmol Vis 55 (13), A5683.

[R154] Martin E, Jiang Y, Padilla S, Fellman R, Zhou Wang RZ, Johnstone M, 2015. Scanning electron microscopy to characterize collector channel anatomic relationships in a non-glaucomatous human eye. Invest. Ophthalmol. Vis. Sci 56, 3259.

[R155] Martin E, M, J., Jamil A, 2010. Aqueous outflow increase resulting from transient blink-induced IOP elevation. Invest. Ophthalmol. Vis. Sci 51, 47.19661234

[R156] Martin E, Orkney N, Johnstone M, 2013. Microspheres (ms) perfused into the anterior chamber (ac) enter the lumen of cylindrical structures spanning schlemm’s canal (sc). Invest. Ophthalmol. Vis. Sci 54, 3534.

[R157] Martin E, Curtiss K, Johnstone MA, 2012. Schlemm’s canal (sc) and distal outflow system relationships revealed by immunohistochemistry (ihc) and confocal microscopy (cfm) following clarification. Invest. Ophthalmol. Vis. Sci 53, 3261.

[R158] Masis Solano M, Lin SC, 2018. Cataract, phacoemulsification and intraocular pressure: is the anterior segment anatomy the missing piece of the puzzle. Prog. Retin. Eye Res 64, 77–83. 29374584 10.1016/j.preteyeres.2018.01.003

[R159] Mazumder A, Roopa T, Basu A, Mahadevan L, Shivashankar GV, 2008. Dynamics of chromatin decondensation reveals the structural integrity of a mechanically prestressed nucleus. Biophys. J 95, 3028–3035. 18556763 10.1529/biophysj.108.132274 PMC2527259

[R160] Mazumder A, Shivashankar GV, 2010. Emergence of a prestressed eukaryotic nucleus during cellular differentiation and development. J. R. Soc. Interface 7, S321–S330. 20356876 10.1098/rsif.2010.0039.focus PMC2943878

[R161] Mcdonnell F, Dismuke WM, Overby DR, Stamer WD, 2018. Pharmacological regulation of outflow resistance distal to schlemm’s canal. Am. J. Physiol. Cell Physiol 315, C44–C51. 29631366 10.1152/ajpcell.00024.2018 PMC6087729

[R162] Mcdonnell F, Perkumas KM, Ashpole NE, Kalnitsky J, Sherwood JM, Overby DR, Stamer WD, 2020. Shear stress in schlemm’s canal as a sensor of intraocular pressure. Sci. Rep 10, 5804. 32242066 10.1038/s41598-020-62730-4 PMC7118084

[R163] Morgan TE, Huber GL, 1967. Loss of lipid during fixation for electron microscopy. J. Cell Biol 32, 757–760. 6034489 10.1083/jcb.32.3.757 PMC2107264

[R164] Morgan WH, Hazelton ML, Yu DY, 2016. Retinal venous pulsation: expanding our understanding and use of this enigmatic phenomenon. Prog. Retin. Eye Res 55, 82–107. 27417037 10.1016/j.preteyeres.2016.06.003

[R165] Nesterov AP, 1970. Role of the blockade of schlemm’s canal in pathogenesis of primary open-angle glaucoma. Am. J. Ophthalmol 70, 691–696. 5477157 10.1016/0002-9394(70)90484-8

[R166] O’Toole MT, 2013. Mosby’s Medical Dictionary. Elsevier Health Sciences, St. Louis.

[R167] Papadopoulou M, Papadaki H, Zolota V, Gartaganis SP, 2017. Immunohistochemical profiles of loxl-1, fbn1, tgf-β1, and cox-2 in pseudoexfoliation syndrome. Curr. Eye Res 42, 880–889. 28085506 10.1080/02713683.2016.1257726

[R168] Parikh HA, Bussel II, Schuman JS, Brown EN, Loewen NA, 2016. Coarsened exact matching of phaco-trabectome to trabectome in phakic patients: lack of additional pressure reduction from phacoemulsification. PLoS One 11, e0149384. 26895293 10.1371/journal.pone.0149384 PMC4760733

[R169] Park DY, Lee J, Park I, Choi D, Lee S, Song S, Hwang Y, Hong KY, Nakaoka Y, Makinen T, Kim P, Alitalo K, Hong YK, Koh GY, 2014. Lymphatic regulator prox1 determines schlemm canal integrity and identity. J. Clin. Invest 124, 3960–3974. 25061877 10.1172/JCI75392 PMC4153702

[R170] Patterson SW, Piper H, Starling EH, 1914. The regulation of the heart beat. J. Physiol 48, 465–513. 16993269 10.1113/jphysiol.1914.sp001676 PMC1420509

[R171] Phelps CD, Asseff CF, Weisman RL, Podos SM, Becker B, 1972. Blood reflux into schlemm’s canal. Arch. Ophthalmol 88, 625–631. 5085206 10.1001/archopht.1972.01000030627011

[R172] Phillips, 2004. Oxford world encyclopedia. Phiillip’s Current Online Version.

[R173] Phillips CI, Tsukahara S, Hosaka O, Adams W, 1992. Ocular pulsation correlates with ocular tension: the choroid as piston for an aqueous pump? Ophthalmic Res 24, 338–343. 1287513 10.1159/000267190

[R174] Picciani R, Desai K, Guduric-Fuchs J, Cogliati T, Morton CC, Bhattacharya SK, 2007. Cochlin in the eye: functional implications. Prog. Retin. Eye Res 26, 453–469. 17662637 10.1016/j.preteyeres.2007.06.002 PMC2064858

[R175] Poley BJ, Lindstrom RL, Samuelson TW, Schulze R, 2009. Intraocular pressure reduction after phacoemulsification with intraocular lens implantation in glaucomatous and nonglaucomatous eyes: evaluation of a causal relationship between the natural lens and open-angle glaucoma. J. Cataract Refract. Surg 35, 1946–1955. 19878828 10.1016/j.jcrs.2009.05.061

[R176] Pomerance A, Ott E, Girvan M, Losert W, 2009. The effect of network topology on the stability of discrete state models of genetic control. Proc. Natl. Acad. Sci. Unit. States Am 106, 8209–8214. 10.1073/pnas.0900142106 PMC2688895 19416903

[R177] Quick CM, Ngo BL, Venugopal AM, Stewart RH, 2009. Lymphatic pump-conduit duality: contraction of postnodal lymphatic vessels inhibits passive flow. Am. J. Physiol. Heart Circ. Physiol 296, H662–H668. 19122167 10.1152/ajpheart.00322.2008 PMC2660222

[R178] Quick CM, Venugopal AM, Dongaonkar RM, Laine GA, Stewart RH, 2008. First-order approximation for the pressure-flow relationship of spontaneously contracting lymphangions. Am. J. Physiol. Heart Circ. Physiol 294, H2144–H2149. 18326809 10.1152/ajpheart.00781.2007

[R179] Quick CM, Venugopal AM, Gashev AA, Zawieja DC, Stewart RH, 2007. Intrinsic pump-conduit behavior of lymphangions. Am. J. Physiol. Regul. Integr. Comp. Physiol 292, R1510–R1518. 17122333 10.1152/ajpregu.00258.2006

[R180] Raviola G, Raviola E, 1981. Paracellular route of aqueous outflow in the trabecular meshwork and canal of schlemm. A freeze-fracture study of the endothelial junctions in the sclerocorneal angel of the macaque monkey eye. Invest. Ophthalmol. Vis. Sci 21, 52–72. 7251302

[R181] Ringvold A, 1978. Actin filaments in trabecular endothelial cells in eyes of the vervet monkey, (cercopithecus aethiops). Acta Ophthalmol (Copenh) 56, 217–225. 96655 10.1111/j.1755-3768.1978.tb01347.x

[R182] Rodbard S, 1975. Vascular caliber. Cardiology 60, 4–49. 126799 10.1159/000169701

[R183] Rohen JW, Futa R, Lütjen-Drecoll E, 1981. The fine structure of the cribriform meshwork in normal and glaucomatous eyes as seen in tangential sections. Invest. Ophthalmol. Vis. Sci 21, 574–585. 7287347

[R184] Rohen JW, Lütjen E, Barany E, 1967. The relation between the ciliary muscle and the trabecular meshwork and its importance for the effect of miotics on aqueous outflow resistance. A study in two contrasting monkey species, macaca irus and cercopithecus aethiops. Albrecht Von Graefes Arch. Klin. Exp. Ophthalmol 172, 23–47. 4967268 10.1007/BF00577152

[R185] Samuelsson B, Troein C, 2003. Super polynomial growth in the number of attractors in kauffman networks. Phys. Rev. Lett 90, 098701. 12689263 10.1103/PhysRevLett.90.098701

[R186] Saraswathy S, Tan JC, Yu F, Francis BA, Hinton DR, Weinreb RN, Huang AS, 2016. Aqueous angiography: real-time and physiologic aqueous humor outflow imaging. PLoS One 11, e0147176. 26807586 10.1371/journal.pone.0147176 PMC4725949

[R187] Saunders DR, Wilson J, Cyrus ER, Novack GD, Van Haarlem T, Kopczynski C, 1967. Loss of absorbed lipid during fixation and dehydration of jejunal mucosa. JCB (J. Cell Biol.) 32, 183–187. 10.1083/jcb.37.1.183 PMC2107391 4868948

[R188] Schirmer KE, 1969. Reflux of blood in the canal of schlemm quantitated. Can. J. Ophthalmol 4, 40–44. 5766336

[R189] Schirmer KE, 1971. Gonioscopic assessment of blood in schlemm’s canal. Correlation with glaucoma tests. Arch. Ophthalmol 85, 263–267. 5542862 10.1001/archopht.1971.00990050265001

[R190] Schwalbe G, 1870. Untersuchungen liber die lymphbahnen des auges und ihre begrenzungen. Arch. Mikrosk. Anat 6, 261–362.

[R191] Sequeira V, Van Der Velden J, 2015. Historical perspective on heart function: the frank-starling law. Biophys Rev 7, 421–447. 28510104 10.1007/s12551-015-0184-4 PMC5418489

[R192] Shapiro AH, 1977a. Physiological and medical aspects of flow in collapsible tubes. In: Modi VJ (Ed.), Proc. Can. Congr. Appl. Mech., 6th Canadian Congress of Applied Mechanics CanCom 77, Proceedings Comtes Rendus, vol. 2, pp. 883–906. Vancouver.

[R193] Shapiro AH, 1977b. Steady flow in collapsible tubes. J. Biomech. Eng 99, 126–147.

[R194] Sims JR, Karp S, Ingber DE, 1992. Altering the cellular mechanical force balance results in integrated changes in cell, cytoskeletal and nuclear shape. J. Cell Sci 103, 1215–1222. 1487498 10.1242/jcs.103.4.1215

[R195] Smit B, Johnstone M, 2000. Effects of viscocanalostomy on the histology of schlemm’s canal in primate eyes. Invest. Ophthalmol. Vis. Sci 41, S578.

[R196] Smit BA, Johnstone MA, 2002. Effects of viscoelastic injection into schlemm’s canal in primate and human eyes: potential relevance to viscocanalostomy. Ophthalmology 109, 786–792. 11927441 10.1016/s0161-6420(01)01006-5

[R197] Smith R, 1956. Blood in the canal of schlemm. Br. J. Ophthalmol 40, 358. 13355941 10.1136/bjo.40.6.358 PMC509496

[R198] Snedeker JG, 2014. The nuclear envelope as a mechanostat: a central cog in the machinery of cell and tissue regulation. BoneKEy Rep 3. 10.1038/bonekey.2014.57 PMC4135537 25177488

[R199] Stambaugh J, Fuhs J, Ascher KW, 1954. Study of the compensation-maxi mum test on aqueous veins. A. M. A. Arch. Ophth 51, 24. 10.1001/archopht.1954.00920040026004 13103882

[R200] Stamer WD, Braakman ST, Zhou EH, Ethier CR, Fredberg JJ, Overby DR, Johnson M, 2015. Biomechanics of schlemm’s canal endothelium and intraocular pressure reduction. Prog. Retin. Eye Res 44, 86–98. 25223880 10.1016/j.preteyeres.2014.08.002 PMC4268318

[R201] Stamer WD, Lei Y, Boussommier-Calleja A, Overby DR, Ethier CR, 2011. Enos, a pressure-dependent regulator of intraocular pressure. Invest. Ophthalmol. Vis. Sci 52, 9438–9444. 22039240 10.1167/iovs.11-7839 PMC3293415

[R202] Stepanik J, 1954. Measuring velocity of flow in aqueous veins. Am. J. Ophthalmol 37, 918. 13158490 10.1016/0002-9394(54)91933-9

[R203] Stepanik J, 1957. Der sichtbare kammerwasserabfluss. Eine kinematographische studie. Klin. Monatsblatter Augenheilkd 130, 208. 13439936

[R204] Stepanik J, Kemper RA, 1954b. Outflow of aqueous humor. Biomicroscopic estimation compared with tonographic measurement. A.M.A. Arch. Ophthal 57, 671. 10.1001/archopht.1954.00920040681011 13147620

[R205] Strenk LM, Guo S, Lu K, Tjan B, Werner L, Strenk S, 2018. The force of lens growth on the uvea. Invest. Ophthalmol. Vis. Sci 59, 2211–2211.

[R206] Strenk SA, Strenk LM, Guo S, 2010. Magnetic resonance imaging of the anteroposterior position and thickness of the aging, accommodating, phakic, and pseudophakic ciliary muscle. J. Cataract Refract. Surg 36, 235–241. 20152603 10.1016/j.jcrs.2009.08.029 PMC2826892

[R207] Suki B, Ito S, Stamenovic D, Lutchen KR, Ingenito EP, 2005. Biomechanics of the lung parenchyma: critical roles of collagen and mechanical forces. J. Appl. Physiol 98, 1892–1899, 1985. 15829722 10.1152/japplphysiol.01087.2004

[R208] Susanna R, De Moraes CG, Cioffi GA, Ritch R, 2015. Why do people (still) go blind from glaucoma. Transl Vis Sci Technol 4, 1. 10.1167/tvst.4.2.1 PMC4354096 25767744

[R209] Suson EB, Schultz RO, 1969. Blood in schlemm’s canal in glaucoma suspects. A study of the relationship between blood-filling pattern and outflow facility in ocular hypertension. Arch. Ophthalmol 81, 808–812. 5783752 10.1001/archopht.1969.00990010810010

[R210] Tamm E, Lütjen-Drecoll E, Jungkunz W, Rohen JW, 1991. Posterior attachment of ciliary muscle in young, accommodating old, presbyopic monkeys. Invest. Ophthalmol. Vis. Sci 32, 1678–1692. 2016145

[R211] Tamm ER, Flugel C, Stefani FH, Lütjen-Drecoll E, 1994. Nerve endings with structural characteristics of mechanoreceptors in the human scleral spur. Invest. Ophthalmol. Vis. Sci 35, 1157–1166. 8125727

[R212] Tamm ER, 2002. Myocilin and glaucoma: facts and ideas. Prog. Retin. Eye Res 21, 395–428. 12150989 10.1016/s1350-9462(02)00010-1

[R213] Tamm ER, Flügel-Koch C, Mayer B, Lütjen-Drecoll E, 1995a. Nerve cells in the human ciliary muscle: ultrastructural and immunocytochemical characterization. Invest. Ophthalmol. Vis. Sci 36, 414–426. 7531186

[R214] Tamm ER, Koch TA, Mayer B, Stefani FH, Lütjen-Drecoll E, 1995b. Innervation of myofibroblast-like scleral spur cells in human monkey eyes. Invest. Ophthalmol. Vis. Sci 36, 1633–1644. 7601644

[R215] Tamm ER, Lütjen-Drecoll E, 1997. Nitrergic nerve cells in the primate ciliary muscle are only present in species with a fovea centralis. Ophthalmologica 211, 201–204. 9176902 10.1159/000310789

[R216] Tamura Y, Konomi H, Sawada H, Takashima S, Nakajima A, 1991. Tissue distribution of type viii collagen in human adult and fetal eyes. Invest. Ophthalmol. Vis. Sci 32, 2636–2644. 1869415

[R217] Teng CC, Paton RT, Katim HM, 1955. Primary degeneration in the vicinity of the chamber angle; as an etiologic factor in wide-angle glaucoma. Am. J. Ophthalmol 40, 619–631. 13268529 10.1016/0002-9394(55)91489-6

[R218] Tervo K, Paallysaho T, Virtanen I, Tervo T, 1995. Integrins in human anterior chamber angle. Graefes Arch. Clin. Exp. Ophthalmol 233, 291–295. 7622078 10.1007/BF00177651

[R219] Thomassen TL, Perkins ES, Dobree JH, 1950. Aqueous veins in glaucomatous eyes. Br. J. Ophthalmol 34, 221. 15411485 10.1136/bjo.34.4.221 PMC1323504

[R220] Thomassen TL, 1947a. On aqueous veins. Acta Ophthalmol 25, 369–378.

[R221] Thomassen TL, 1947b. The venous tension of eyes suffering from simple glaucoma. Acta Ophthalmol 25, 221.

[R222] Thomson BR, Heinen S, Jeansson M, Ghosh AK, Fatima A, Sung HK, Onay T, Chen H, Yamaguchi S, Economides AN, Flenniken A, Gale NW, Hong YK, Fawzi A, Liu X, Kume T, Quaggin SE, 2014. A lymphatic defect causes ocular hypertension and glaucoma in mice. J. Clin. Invest 124, 4320–4324. 25202984 10.1172/JCI77162 PMC4191022

[R223] Tomarev SI, Wistow G, Raymond V, Dubois S, Malyukova I, 2003. Gene expression profile of the human trabecular meshwork: Neibank sequence tag analysis. Invest. Ophthalmol. Vis. Sci 44, 2588–2596. 12766061 10.1167/iovs.02-1099

[R224] Troncoso MU, 1925. Gonioscopy and its clinical applications. Am. J. Ophthalmol 8, 433.

[R225] Vahabikashi A, Gelman A, Dong B, Gong L, Cha EDK, Schimmel M, Tamm ER, Perkumas K, Stamer WD, Sun C, Zhang HF, Gong H, Johnson M, 2019.Increased stiffness and flow resistance of the inner wall of schlemm’s canal in glaucomatous human eyes. Proc. Natl. Acad. Sci. U. S. A 116, 26555–26563. 10.1073/pnas.1911837116 PMC6936716 31806762

[R226] Van Buskirk EM, 1976. Changes in facility of aqueous outflow induced by lens depression and intraocular pressure in excised human eyes. Am. J. Ophthalmol 82, 736–730. 998694 10.1016/0002-9394(76)90011-8

[R227] Van Buskirk EM, 1982. Anatomic correlates of changing aqueous outflow facility in excised human eyes. Invest. Ophthalmol. Vis. Sci 22, 625–632. 7076408

[R228] Van Buskirk EM, Grant WM, 1973. Lens depression and aqueous outflow in enucleated primate eyes. Am. J. Ophthalmol 76, 632–640. 4201217 10.1016/0002-9394(73)90555-2

[R229] Van Buskirk EM, Grant WM, 1974. Influence of temperature and the question of involvement of cellular metabolism in aqueous outflow. Am. J. Ophthalmol 77, 565–572. 4819458 10.1016/0002-9394(74)90472-3

[R230] Van Zyl T, Yan W, Mcadams A, Peng YR, Shekhar K, Regev A, Juric D, Sanes JR, 2020. Cell atlas of aqueous humor outflow pathways in eyes of humans and four model species provides insight into glaucoma pathogenesis. Proc. Natl. Acad. Sci. U.S.A 117, 10339–10349. 32341164 10.1073/pnas.2001250117 PMC7229661

[R231] Vranka JA, Kelley MJ, Acott TS, Keller KE, 2015. Extracellular matrix in the trabecular meshwork: intraocular pressure regulation and dysregulation in glaucoma. Exp. Eye Res 133, 112–125. 25819459 10.1016/j.exer.2014.07.014 PMC4379427

[R232] Wang K, Johnstone MA, Xin C, Song S, Padilla S, Vranka JA, Acott TS, Zhou K, Schwaner SA, Wang RK, Sulchek T, Ethier CR, 2017. Estimating human trabecular meshwork stiffness by numerical modeling and advanced oct imaging. Invest. Ophthalmol. Vis. Sci 58, 4809–4817. 28973327 10.1167/iovs.17-22175 PMC5624775

[R233] Wang N, Butler JP, Ingber DE, 1993. Mechanotransduction across the cell surface and through the cytoskeleton. Science 260, 1124–1127. 7684161 10.1126/science.7684161

[R234] Wang RK, Nuttall AL, 2010. Phase-sensitive optical coherence tomography imaging of the tissue motion within the organ of corti at a subnanometer scale: a preliminary study. J. Biomed. Optic 15, 056005. 10.1117/1.3486543 PMC2948044 21054099

[R235] Wang RK, Kirkpatrick SJ, Hinds M, 2007. Phase-sensitive optical coherence elastography for mapping tissue microstrains in real time. Appl. Phys. Lett 90, 164105.

[R236] Wang RK, Ma Z, Kirkpatrick SJ, 2006. Tissue Doppler optical coherence elastography for real time strain rate and strain mapping of soft tissue. Appl. Phys. Lett 89, 144103.

[R237] Waxman S, Loewen RT, Dang Y, Watkins SC, Watson AM, Loewen NA, 2018a. High-resolution, three-dimensional reconstruction of the outflow tract demonstrates segmental differences in cleared eyes. Invest. Ophthalmol. Vis. Sci 59, 2371–2380. 29847643 10.1167/iovs.17-23075 PMC5939687

[R238] Waxman S, Wang C, Dang Y, Hong Y, Esfandiari H, Shah P, Lathrop KL, Loewen RT, Loewen NA, 2018b. Structure-function changes of the porcine distal outflow tract in response to nitric oxide. Invest. Ophthalmol. Vis. Sci 59, 4886–4895. 30347083 10.1167/iovs.18-24943 PMC6181305

[R239] Weinreb RN, Ryder MI, 1990. In situ localization of cytoskeletal elements in the human trabecular meshwork and cornea. Invest. Ophthalmol. Vis. Sci 31, 1839–1847. 2211030

[R240] Wiederholt M, Thieme H, Stumpff F, 2000. The regulation of trabecular meshwork and ciliary muscle contractility. Prog. Retin. Eye Res 19, 271–295. 10749378 10.1016/s1350-9462(99)00015-4

[R241] Xin C, Johnstone M, Wang N, Wang RK, 2016. Oct study of mechanical properties associated with trabecular meshwork and collector channel motion in human eyes. PLoS One 11, e0162048. 27598990 10.1371/journal.pone.0162048 PMC5012558

[R242] Xin C, Song S, Johnstone M, Wang N, Wang RK, 2018. Quantification of pulse-dependent trabecular meshwork motion in normal humans using phase-sensitive oct. Invest. Ophthalmol. Vis. Sci 59, 3675–3681. 30029254 10.1167/iovs.17-23579 PMC6054426

[R243] Xin C, Wang RK, Song S, Shen T, Wen J, Martin E, Jiang Y, Padilla S, Johnstone M, 2017. Aqueous outflow regulation: optical coherence tomography implicates pressure-dependent tissue motion. Exp. Eye Res 158, 171–186. 27302601 10.1016/j.exer.2016.06.007 PMC5272871

[R244] Zhao Z, Zhu X, He W, Jiang C, Lu Y, 2016. Schlemm’s canal expansion after uncomplicated phacoemulsification surgery: an optical coherence tomography study. Invest. Ophthalmol. Vis. Sci 57, 6507–6512. 27918824 10.1167/iovs.16-20583

[R245] Zhou L, Maruyama I, Li Y, Cheng EL, Yue BY, 1999. Expression of integrin receptors in the human trabecular meshwork. Curr. Eye Res 19, 395–402. 10520215 10.1076/ceyr.19.5.395.5297

[R246] Zhou L, Zhang SR, Yue BY, 1996. Adhesion of human trabecular meshwork cells to extracellular matrix proteins. Roles and distribution of integrin receptors. Invest. Ophthalmol. Vis. Sci 37, 104–113. 8550313

[R247] Zhou Z, Johnstone M, Wang R, 2015. Systematic intraluminal disruption of schlemm’s canal structural features alters pressure-dependent changes in sc and collector channel lumen dimensions. Invest. Ophthalmol. Vis. Sci 56, 3259.

